# A Robust and Universal Metaproteomics Workflow for Research Studies and Routine Diagnostics Within 24 h Using Phenol Extraction, FASP Digest, and the MetaProteomeAnalyzer

**DOI:** 10.3389/fmicb.2019.01883

**Published:** 2019-08-16

**Authors:** Robert Heyer, Kay Schallert, Anja Büdel, Roman Zoun, Sebastian Dorl, Alexander Behne, Fabian Kohrs, Sebastian Püttker, Corina Siewert, Thilo Muth, Gunter Saake, Udo Reichl, Dirk Benndorf

**Affiliations:** ^1^Bioprocess Engineering, Otto von Guericke University Magdeburg, Magdeburg, Germany; ^2^Database Research Group, Otto von Guericke University Magdeburg, Magdeburg, Germany; ^3^Bioinformatics Research Group, University of Applied Sciences Upper Austria, Hagenberg, Austria; ^4^glyXera GmbH, Magdeburg, Germany; ^5^Bioprocess Engineering, Max Planck Institute for Dynamics of Complex Technical Systems Magdeburg, Magdeburg, Germany; ^6^Bioinformatics Unit (MF 1), Department for Methods Development and Research Infrastructure, Robert Koch Institute, Berlin, Germany

**Keywords:** bioinformatics, software, sample preparation, environmental proteomics, microbial communities, mass spectrometry, gut microbiome

## Abstract

The investigation of microbial proteins by mass spectrometry (metaproteomics) is a key technology for simultaneously assessing the taxonomic composition and the functionality of microbial communities in medical, environmental, and biotechnological applications. We present an improved metaproteomics workflow using an updated sample preparation and a new version of the MetaProteomeAnalyzer software for data analysis. High resolution by multidimensional separation (GeLC, MudPIT) was sacrificed to aim at fast analysis of a broad range of different samples in less than 24 h. The improved workflow generated at least two times as many protein identifications than our previous workflow, and a drastic increase of taxonomic and functional annotations. Improvements of all aspects of the workflow, particularly the speed, are first steps toward potential routine clinical diagnostics (i.e., fecal samples) and analysis of technical and environmental samples. The MetaProteomeAnalyzer is provided to the scientific community as a central remote server solution at www.mpa.ovgu.de.

## Introduction

The metabolism of microbial communities is determined by the proteome, the total set of proteins of the microbial cells, including enzymes for growth and maintenance. The expression of proteins depends on the environmental conditions, community composition, and the metabolic activity of the individual microorganisms (Wasinger et al., 1995). Metaproteomics, the identification of microbial proteins using MS (Wilmes and Bond, 2006), is crucial to understand microbial communities. Due to the rapid development of MS, the number of conducted metaproteomics studies has increased over the last years. Microbiomes from the human gut (Kolmeder et al., 2012; Xiong et al., 2015; Zhang et al., 2018a), rumen (Deusch et al., 2017), soil (Bastida and Jehmlich, 2016; Keiblinger et al., 2016), or BGPs (Heyer et al., 2016; Hagen et al., 2017) were measured. Metaproteomics aims at deeper insights into microbiomes by analyzing taxonomic and functional composition of complex microbial communities in diverse environments and technical applications. Based on metaproteome data the state of microbial communities can be linked with certain environmental conditions or process parameters. However, metaproteomics also has the potential to serve as a tool for diagnostics in clinical settings or routine process monitoring (Heyer et al., 2017). For example, proteins of the microbial community in the human gut or in a BGP may represent valuable markers for diseases or process disturbances in BGP, respectively. Such routine application of metaproteomics is not common yet, due to two major challenges (i) sample preparation due to high complexity and contamination of samples, and (ii) data analysis due to the required computational effort for large datasets, missing corresponding annotated protein sequence databases, and protein inference causing ambiguity of protein annotation.

The first challenge is the time-consuming sample preparation workflow and its sensitivity to sample impurities (Heyer et al., 2015). Common metaproteomics workflows comprise of protein extraction and purification, tryptic digestion of proteins into peptides, and measurement by LC-MS/MS. The amount of extracted proteins is measured by different assays, and the complexity of protein extracts is often reduced by fractionation using sodium dodecyl sulfate polyacrylamide gel electrophoresis (SDS-PAGE) (Heyer et al., 2015; Wenzel et al., 2018) or two dimensional chromatography (Erickson et al., 2012; Kleiner et al., 2017). In consequence, the total workflow for sample preparation can take up to 1 week, but routine diagnostics should not exceed 24 h for complete analysis. Therefore, we choose to sacrifice fractionation, since monitoring of the main microbial processes and highly abundant marker proteins do not require such a high coverage of the metaproteome. Different protocols exist for protein extraction and protein purification (Keiblinger et al., 2012; Zhang et al., 2018b), depending on the sample type. Samples from microbial communities from fresh water or the ocean are almost free of impurities, and proteins can be extracted easily (Colatriano and Walsh, 2015). In contrast, soil and BGP samples contain high amounts of humic substances (Heyer et al., 2015; Keiblinger et al., 2016), which require specialized extraction methods such as phenol extraction (Heyer et al., 2013) or trichloroacetic acid precipitation (Chourey et al., 2010). Adaptation of the workflow for each sample type is time consuming and not feasible for routine application, therefore, we choose phenol extraction in this study, since it provides robust protein recovery from different sample types (Benndorf et al., 2007, 2009; Keiblinger et al., 2012; Heyer et al., 2013; Püttker et al., 2015).

The second challenge concerns the data analysis. Proteins are commonly identified by comparing experimental peptide spectra against theoretical spectra derived from protein sequence databases (Mann and Wilm, 1994). Subsequently, identified proteins are assigned by taxonomy and function. However, three issues specific to metaproteomics hamper and delay bioinformatics evaluation (Muth et al., 2013). First, the amount of acquired data is huge due to the high complexity of microbial communities, which results in enormous demands regarding computing resources. Modern LC-MS/MS instruments produce tens of thousands high-resolution spectra per hour. This enables in-depth analysis of the metaproteome but increases the computational load significantly. Second, protein identification can be difficult due to the lack of suitable protein or metagenome databases. Third, the interpretation of taxonomic and functional results is difficult due to the problem of protein inference (Nesvizhskii and Aebersold, 2005) from conserved sequences in homologous proteins.

To tackle these issues, the MPA was developed as an intuitive open-source software platform for metaproteomics data analysis and interpretation (Muth et al., 2015a). Among other features, it supports the handling of protein inference by grouping proteins into protein groups (called metaproteins hereafter). The generation of metaproteins is a strategy that was developed specifically for the metaproteomics field. The latest implementation of the MPA (version 3.0.0) also allows for easy comparison of results from different experiments and provides supplementary annotation functions for protein entries from metagenome sequences (regarding taxonomies or protein functions).

In this paper, a complete metaproteomics workflow is described where all processing steps from sample preparation to visualization are performed within 24 h, referred to as “new workflow” hereafter. The objectives of our new protocols were speed, simplicity, high throughput, reproducibility, and robustness to establish metaproteomics as routine application in applied research and diagnostics. This new workflow was applicable to various types of samples and drastically decreased overall processing time from at least 3 days to only 1 day. The aim of the presented workflow was not to provide discovery oriented, in-depth analysis of microbial communities. Instead, it constituted an important milestone toward routine monitoring of biotechnological processes and analysis of clinical samples, since such routine analyses should not exceed a 24 h time period or require complicated adaptations of the laboratory procedures. In order to achieve this goal, phenol extraction was optimized compared to previous studies (Heyer et al., 2013), in-gel digestion was replaced by FASP digestion (new sample preparation)(Wisniewski et al., 2009), and the MPA software (Muth et al., 2015a) was continuously updated (current MPA version 3.0.0).

## Materials and Methods

**FIGURE 1:**
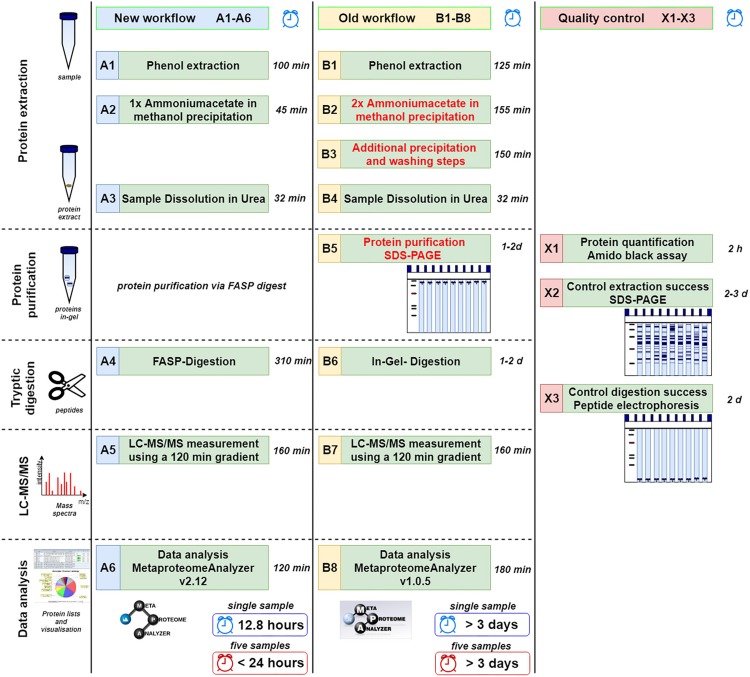
Comparison of new **(A1–A6)** and old workflow **(B1–B8)** for metaproteomics sample preparation and analysis. In addition, methods for quality control are shown **(X1–X3)**. The time shown represents the shortest possible time in which a single sample can be processed. Under reasonable circumstances five samples can be done in less than 24 h (or 15 samples within 48 h) using the new workflow limited by the number of available mass spectrometer. Similarly, at least 3 days are required for multiple samples using the old workflow.

For an overview, refer to the complete workflow steps A1-A6, B1-B8, and X1-X3 in Figure 1. For a more detailed descriptions and chemicals please consider the SOPs listed as Supplementary Data Sheet S1.

### Improvements of the Laboratory Workflow

In order to reduce the time required for the phenol extraction protocol, dispensable washing steps with organic solvents were removed and incubation times were reduced. Protein purification by SDS-PAGE (Kohrs et al., 2014) and subsequent in-gel-digestion into peptides were the most time-consuming steps of old workflows, and were replaced with the FASP protocol (Wisniewski et al., 2009). The FASP protocol replaced these steps, allowing for direct digestion and simultaneous purification of the protein extract on the FASP filter (Wisniewski et al., 2009). In contrast to previous applications of the FASP protocol to environmental samples (Tanca et al., 2014; Brum et al., 2016), several steps of the FASP digestion were optimized. In particular, trypsin incubation time was reduced from the previous 12 h (overnight) to only 2 h (Supplementary Table S1). Furthermore, re-buffering of peptide extracts by time-consuming lyophilisation was omitted. Instead, extracts after FASP digestion were injected directly into the LC-MS/MS system after acidification.

### Improvements of the MetaProteomeAnalyzer Software

**FIGURE 2:**
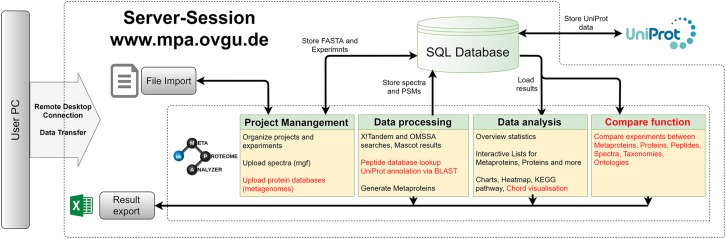
MetaProteomeAnalyzer. Workflow of the MetaProteomeAnalyzer software including improvements and additions to the first MetaProteomeAnalyzer version (Muth et al., 2015a). Improvements were highlighted in red.

**FIGURE 3:**
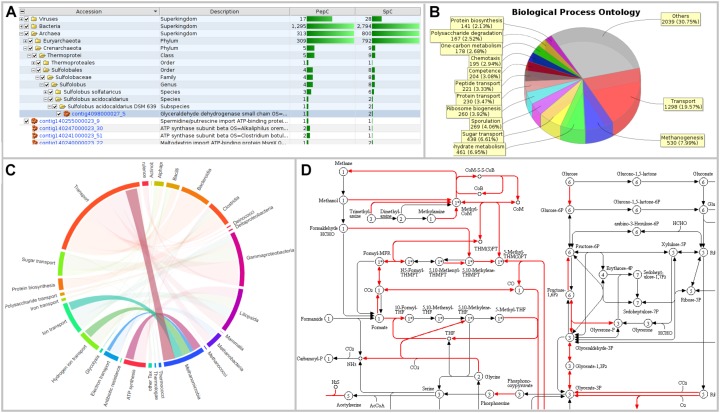
Visualizations of MetaProteomeAnalyzer using data from NewWF_BGP_3_B. **(A)** The taxonomy results view of the protein tables hierarchically orders proteins by taxonomy allowing for easy selection and filtering of specific taxonomies. **(B)** Pie Chart with spectral counts of the biological process ontology of the Phylum *Euryarchaeota* selected through the taxonomy view. **(C)** Interactive chord diagram visualizing the relationship between taxonomy (rank = family) and functional ontology (UniProt keywords for Biological Process) (Zoun et al., 2017). Biological processes for *Methanosarcinaceae*, as an example, are highlighted. **(D)** KEGG pathway map for central carbon metabolisms (KEGG map 01200) highlighting enzymes identified with the MPA.

An updated version of the MPA software was developed (see Figures 2, 3). It not only improved the existing features but also added new functionalities (Muth et al., 2015a). The MPA offered a complete workflow from peak lists exported by the MS-software to protein database searching, and result analysis, visualization and export. A major feature of the MPA was the grouping of proteins into metaproteins based on shared peptides or sequence similarity. The provided manual (Supplementary Table S2) gives an in-depth description of the new version of the MPA software. Video tutorials, the download and other material are available on the MPA website^1^. All analyses for this manuscript were carried out with MPAv2.2.12. Meanwhile the version number was updated to number 3.0.0, which contains only minor changes.

Memory and speed constraints were reduced by improving the existing implementation of the algorithms and the database queries. Metagenome databases can now be uploaded by the user, providing a more user-friendly and efficient access. Further improvements include an update of internal parser routines, and the retrieval of protein meta-information using UniProtJAPI (Patient et al., 2008) for obtaining complete protein databases during upload. Additionally, the database search engines X!Tandem (Craig and Beavis, 2004) and OMSSA (Geer et al., 2004) were supplemented with a peptide database lookup. Furthermore, an integrated protein BLAST allowed the user to link unannotated protein sequences with UniProt metadata. The new MPA version now includes a sample comparison function that allows for a quantitative comparison of metaproteins, taxonomies, and protein ontologies across a multitude of samples. The newly implemented cord diagram tool visualizes taxonomy-function-relationships (Zoun et al., 2017).

### Sampling

For this study, a total of nine samples were taken: three BGP samples (BGP_1–3), three human gut samples (Hgut_1–3), a soil sample, a compost sample and one WWTP sample. Samples were stored directly at −20°C. For subsequent phenol extraction, samples were defrosted and weighed. For processing of the WWTP sample, sludge flakes were centrifuged (10 min, 4°C, 10,000 g) before weighing and discarding of the supernatant.

### Phenol Extraction (A1, B1)

For phenol extraction (Supplementary Data Sheet S1), 2 g sample, 5 g silica beads (0.5 mm), 2 mL 2 M sucrose solution, and 3.5 mL phenol solution (10 g phenol dissolved in 1 mL ultrapure water) were added to a 15 mL reaction tube. Subsequently, the falcon was transferred into a ball mill (FastPrep-96, MP Biomedicals, Eschwege, Germany) and shaken for 5 min (A1) resp. 30 min (B1) at RT and 1,800 rpm. After centrifugation (10 min, RT, 10,000 *g*), the upper phenol phase was collected into a new 15 mL reaction tube and washed with the same volume of 1 M sucrose solution for 10 min at RT and 120 rpm on a shaker/ball mill. Finally, the sample was centrifuged again (10 min, RT, 10,000 g), and proteins were precipitated by ammonium acetate in methanol precipitation.

### Ammonium Acetate in Methanol Precipitation (A2, B2)

Addition of the fourfold volume of ice-cold 100 mM ammonium acetate in methanol for 20 min (A2) resp. 60 min (B2) at −20°C precipitated proteins in the phenol phase. Afterward, the sample was centrifuged (10 min, 4°C, 10,000 g), and the supernatant was discarded. This precipitation step was repeated once.

### Further Washing Steps (B3)

In order to remove remaining impurities, the precipitated protein pellet was washed four times with a threefold volume of ice-cold 80% acetone, 70% ethanol, 80% acetone, and 70% ethanol. Between the washing steps, the sample was incubated at −20°C, centrifuged (10 min, RT, 10,000 *g*) and the supernatant was discarded.

### Dissolution of the Sample in Urea (A3, B4)

Finally, the protein pellet was dried at 60°C for 15 min and dissolved in 1 mL urea buffer (7 M urea, 2 M thiourea, 1% DTT). After 10 min shaking in a ball mill at (RT, 1,800 rpm), non-dissolved particles were removed by centrifugation (10 min, 4°C, 10,000 *g*). Protein extracts were stored at −20°C for later use.

### Protein Quantification Using Amido Black Assay (X1)

For quantification of protein concentration (Supplementary Data Sheet S1) 50 μL of the sample were precipitated with 300 μL amido black staining solution. Afterward, the sample was centrifuged (5 min, RT, 16,400 *g*) and the supernatant was discarded. Two washing steps with 10% acetic acid in methanol and two centrifugation steps (5 min, RT, 16,400 *g*) removed unbound dye. Finally, the pellet was dissolved in 1 mL 0.1 M sodium hydroxide and absorption was measured at wavelength 615 nm using a photometer (Spectrophotometer Genesys 10S UV-Vis, Thermo Scientific, Waltham, United States).

### SDS-PAGE (B5, X2)

For SDS-PAGE (Supplementary Data Sheet S1), 100 μg protein extract was diluted with the same volume of ultrapure water and precipitated by the same volume of ice-cold 100% acetone. After incubation at −20°C overnight, samples were centrifuged (30 min, 4°C, 16,400 *g*), the supernatant was discarded, and the pellet was dried. Subsequently, the protein pellet was dissolved in 20 μL SDS sample buffer, the sample was centrifuged (30 min, 4°C, 16,400 *g*), and the supernatant was loaded on the SDS-PAGE. In parallel to sample preparation, a 1 mm SDS-PAGE gel was prepared using a 12% separation and a 4% stacking gel. Subsequently, SDS-PAGE gels were inserted into the SDS-PAGE chamber (Mini-Protean Tetra System, BioRad, Hercules, United States), and the samples were loaded. Finally, 10 mA current was applied until proteins entered the separation gels, then 20 mA until the end of the gel. For subsequent in-gel digestion, the electrophoresis was stopped after the dye front entered into the separation gel for 5 mm. For visualization, proteins were incubated for 1 h in fixation solution (40% ethanol, 10% acetic acid) and then stained with Coomassie staining solution.

### Peptide Electrophoresis (X3)

Peptide electrophoresis (Supplementary Data Sheet S1) was conducted in a standard electrophoresis chamber (Mini-Protean Tetra System, BioRad, Hercules, United States) (Schägger, 2006). In brief, 90 μg peptides were precipitated with acetone, diluted in 10 μL sample buffer, and incubated in a thermomixer for 60 min at 37°C and 1,400 rpm. Afterward, samples were centrifuged (10 min, 4°C, 16,400 *g*) and the supernatant was loaded on the gel. The gel comprised a 4% stacking gel as well as a 10% and a 16% separation gel. For separation, a voltage of 30 V was applied until the running front entered the 10% separation gel and increased subsequently to 90 V until it reached the end of the gel. Protein staining with Coomassie was carried out analogously to the staining of SDS-PAGEs, but the fixation solution contained methanol instead of ethanol.

### FASP Digestion (A4)

For the FASP digestion (Supplementary Data Sheet S1), 100 μg protein extract in 200 μL urea buffer were loaded onto the FASP filter (Pall Nanosep 10K Omega, MWCO 10 kDa) and centrifuged (10–20 min, RT, 10,000). Note: Soil and human fecal samples required longer centrifugation times until all liquid passed through the FASP filter (about 20 min). Reduction and alkylation of proteins were carried out by addition of 100 μL DTT (20 min, 56°C, 300 rpm) and 100 μL IAA (20 min, RT, 300 rpm, in the dark). After each of these steps the liquid was removed by centrifugation (5 min, RT, 10,000 *g*) and the flow through was discarded. Subsequently, the proteins were washed once for 2 min with 100 μL 8 M urea, three times with 100 μL 50 mM ammonium bicarbonate, and centrifuged afterward (5 min, RT, 10,000 *g*). After removal of the flow through, trypsin was added onto the FASP filter (2 h, 37°C, 300 rpm) in an enzyme to protein ratio of approximately 1–100. Subsequently, the sample was centrifuged (5 min, RT, 10,000 *g*). Remaining peptides were rinsed through the filter by addition of 50 μL 50 mM ammonium bicarbonate and 50 μL ultrapure water (Millipore Q-POD Merck, Darmstadt, Germany) followed by another centrifugation step (5 min, RT, 10,000 *g*). Finally, 30 μL were acidified by addition of 3 μL 0.5% TFA, centrifuged (10 min, 4°C, 10,000 *g*), and transferred into an HPLC vial.

### In-Gel Digestion (B6)

The single protein fraction after early stopping SDS-PAGE was cut into cubes of approx. 1 mm side length and transferred into a 2 mL reaction tube. For removal of the Coomassie dye, the gel cubes were incubated in 900 μL washing solution (50% methanol, 45% ultrapure water, 5% acetic acid) twice, once overnight and once the next day for 1 h in a shaker (RT, 150 rpm). After a further washing step with 900 μL acetonitrile (10 min, RT, 150 rpm), gel cubes were dried in a vacuum centrifuge (Digital Series SpeedVac SPD121P, Thermo Scientific, Waltham, United States). Reduction and alkylation of proteins were carried out by addition of 900 μL DTT (30 min, RT, 150 rpm) and 900 μL IAA (30 min, RT, 150 rpm, in the dark). After each of these steps, gel cubes were incubated in 900 μL acetonitrile (10 min, RT, 150 rpm). Subsequently, the gel cubes were washed with 50 mM ammonium bicarbonate (10 min, RT, 150 rpm) and acetonitrile (10 min, RT, 150 rpm). For tryptic digestion of proteins, 200 μL trypsin buffer (enzyme to substrate ratio: 1:100) was added over night (37°C, 150 rpm). The next day, the supernatant was collected into a new 2 mL reaction tube. Remaining peptides were washed out of the gel by incubation in extraction buffer 1 (90% ultrapure water, 10% formic acid; 30 min, RT, 150 rpm) and extraction buffer 2 (50% ultrapure water, 49% ACN, 1% TFA; 30 min, RT, 150 rpm). Both extracts were collected in a new reaction tube. Finally, the peptide solution was dried in the vacuum centrifuge and stored at −20°C. For LC-MS/MS measurements, dried peptides were dissolved in 300 μl solvent A (98% ultrapure water, 2% acetonitrile, 0.05% TFA), centrifuged (30 min, 4°C, 13,000 *g*) and transferred into a HPLC-vial.

### LC-MS/MS Measurements (A5, B7)

Peptides were analyzed by LC-MS/MS using an UltiMate 3000 RSLCnano splitless liquid chromatography system coupled online to an Orbitrap Elite^TM^ Hybrid Ion Trap-Orbitrap MS/MS (MS) (both from Thermo Fisher Scientific, Bremen, Germany). After injection, peptides were loaded isocratically on a trap column (Dionex Acclaim, nano trap column, 100 μm i.d. × 2 cm, PepMap100 C18, 5 μm, 100 Å, nanoViper) with a flow rate of 7 μL/min chromatographic liquid phase A (98% ultrapure water, 2% acetonitrile, 0.05% TFA) for desalting and concentration.

Chromatographic separation was performed on a Dionex Acclaim PepMap C18 RSLC nano reversed phase column (2 μm particle size, 100 Å pore size, 75 μm inner diameter, and 250 mm length) at 40°C column temperature. A flow rate of 250 nL/min was applied using a binary A/B-solvent gradient (solvent A: 98% ultrapure water, 2% acetonitrile, 0.1% formic acid; solvent B: 80% acetonitrile, 10% ultrapure water, 10% trifluorethanol, 0.1% formic acid). 5 μl sample were injected. Separation started with 4% B for 5 min, continued with a linear increase to 55% B within 120 min, followed by a column wash with 90% B for 5 min, and re-equilibration with 4% B for 25 min. For mass spectrometry acquisition, a data-dependent MS/MS method was chosen. For the conducted measurements the MS was operated in positive ion mode and precursor ions were acquired in the orbital trap of the hybrid MS at a resolution of 30,000 and an *m/z* range of 350–2,000. Subsequently, fragment ion scans were produced in the linear ion trap of the hybrid MS with mass range and a scan rate at “normal” parameter settings for the top 20 most intense precursors selected for collision-induced dissociation.

### Protein Identification Using the MPA (A7)

Orbitrap Elite^TM^ Hybrid Ion Trap-Orbitrap MS/MS measurements raw data files (raw file format) were processed by the Proteome Discoverer Software 1.4 (version 1.4.1.14, Thermo Fisher Scientific, Bremen, Germany), and converted into the Mascot Generic File format (mgf). Subsequently, mgf files were uploaded into the MPA software in the new version 2.12 and the release version 1.0.5 that was published previously (Muth et al., 2015a).

Three different types of software were used for peptide spectral matching: X!Tandem (Craig and Beavis, 2004), OMSSA (Geer et al., 2004) and MASCOT (version 2.5, Matrix Science, London, England) (Perkins et al., 1999). The MASCOT search was managed by the ProteinScape software (Bruker Daltonics, Bremen, Deutschland, (version 4.0.3 315) (Chamrad et al., 2007). All protein database searches used the following parameters: enzyme trypsin, one missed cleavage, monoisotopic mass, carbamidomethylation (cysteine) as fixed modification, oxidation (methionine) as variable modifications, ±10 ppm precursor and ± 0.5 Da MS/MS fragment tolerance, 1^13^C and +2/+3 charged peptide ions. The Mascot search results (dat file format) were uploaded to the MPA software (only version 2.12). The MPA was designed to do the ensemble search (multiple search engines). Results were combined by uniquely identifying spectra and peptides throughout data processing. Therefore, spectra and peptides were not duplicated when multiple search engines reported the same match. In the rare case that two different peptides were found for a single spectrum both results were written into the database. This is not accurate with respect to spectral counting for quantification but kept as much information as possible.

Four protein databases – one for each sample type – were used for protein database searches (Table 1). These databases were created by combining UniProtKB/SwissProt (release November 2017) with an appropriate metagenome. Peptides found by X!Tandem and OMSSA searches were associated with all proteins containing them using a dedicated peptide database generated from the four protein databases prior to searches (peptide database lookup).

**TABLE 1:** Source and size of protein sequence databases.

**Database**	**Protein sequences**	**Source/Reference**	**Used for samples**
Biogas + SwissProt	2,349,714	Schluter et al., 2008; Rademacher et al., 2012; Hanreich et al., 2013; Stolze et al., 2016	BGP
Human Gut + SwissProt	6,159,039	Qin et al., 2010 https://www.ebi.ac.uk/metagenomics/studies/ERP000108	Hgut
Soil + SwissProt	684,487	JGI sequencing project; https://gold.jgi.doe.gov/study?id=Gs0085736	Soil compost
WWTP + SwissProt	2,243,839	Albertsen et al., 2012	WWTP
SwissProt	556,196	SwissProt downloaded in November 2017 www.uniprot.org	

A false discovery rate (FDR) was applied at the PSM level. With the exception of soil and compost samples, an FDR of 1% was applied to all other samples. The old laboratory workflow did not report any proteins for soil and compost if the FDR was set to 1%. Therefore, the FDR of 5% was chosen for soil samples to allow for a fair comparison between the old and new workflows. In MPA version 2.12, identified proteins without taxonomic and functional classification were annotated with UniProtKB metadata by using protein BLAST [NCBI-Blast-version 2.6.0 (Altschul et al., 1990; Camacho et al., 2009)] against the UniProtKB/SwissProt database using an *e*-value cutoff of 10^–4^. Subsequently, all protein BLAST proposals with the best identity were merged and used to annotate a protein.

Proteins were grouped into metaproteins using the shared peptide rule. The shared peptide rule adds a protein to the metaprotein if it has at least one distinct peptide in common with any other protein that belongs to this metaprotein. This did not require that all proteins of a metaprotein shared the same peptide. Metaproteins generated in this way were given a merged annotation. The taxonomy and UniRef Cluster of the metaprotein is set as the common ancestor of its proteins, while functional keywords and KEGG orthologies are compiled into non-redundant lists.

Several statistics for each sample were collected using the MPA software (Supplementary Table S3) and the metaproteins as well as metaprotein taxonomies were exported as comma separated value files (version 2.12 and version 1.0.5) (Supplementary Table S4). The sample comparison feature of MPA version 2.12 was used to generate metaproteins among all 54 samples and the resulting table was exported for later analysis. For quantification the spectral counts were taken. Finally, all MS data were submitted to PRIDE (Vizcaino et al., 2016) with the accession number PXD010550.

### Biostatistics Evaluation

The data collected through the MPA software (Supplementary Table S4) were used to calculate the average number of identified spectra, peptides, proteins, and metaproteins. Metaproteins were split into known and unknown proteins depending on the existence of metadata beyond the protein sequence (i.e., taxonomy). The taxonomy distribution was calculated by counting the occurrence of specific taxonomies at all taxonomic ranks (Supplementary Table S5). The results of the comparison function were exported as a single csv file (Supplementary Table S6), and principle coordinate analysis (PCoA) was carried out using PAST3 (version 3.20).

## Results

The evaluation of the new workflow was divided into two steps: (i) improvements of the laboratory workflow and (ii) improvements of the bioinformatic workflow.

### Improvements of the Laboratory Workflow

#### Validation of Protein Extraction

Phenol extraction from 2 g sample material resulted in between 0.55 and 10.94 mg protein per sample (Supplementary Table S7). To obtain sufficient protein for soil samples, pooling of seven extracts was required. Protein concentrations of previous and new sample preparations were similar (see Supplementary Table S7). Observed variation in protein amounts between sample types indicated that protein quantification of new samples should be performed to guarantee equal protein loading for FASP digestion and MS. For samples with limited availability, less raw material could be extracted because for protein quantification, FASP digestion and mass spectrometry, about 100 μg protein are required.

**FIGURE 4:**
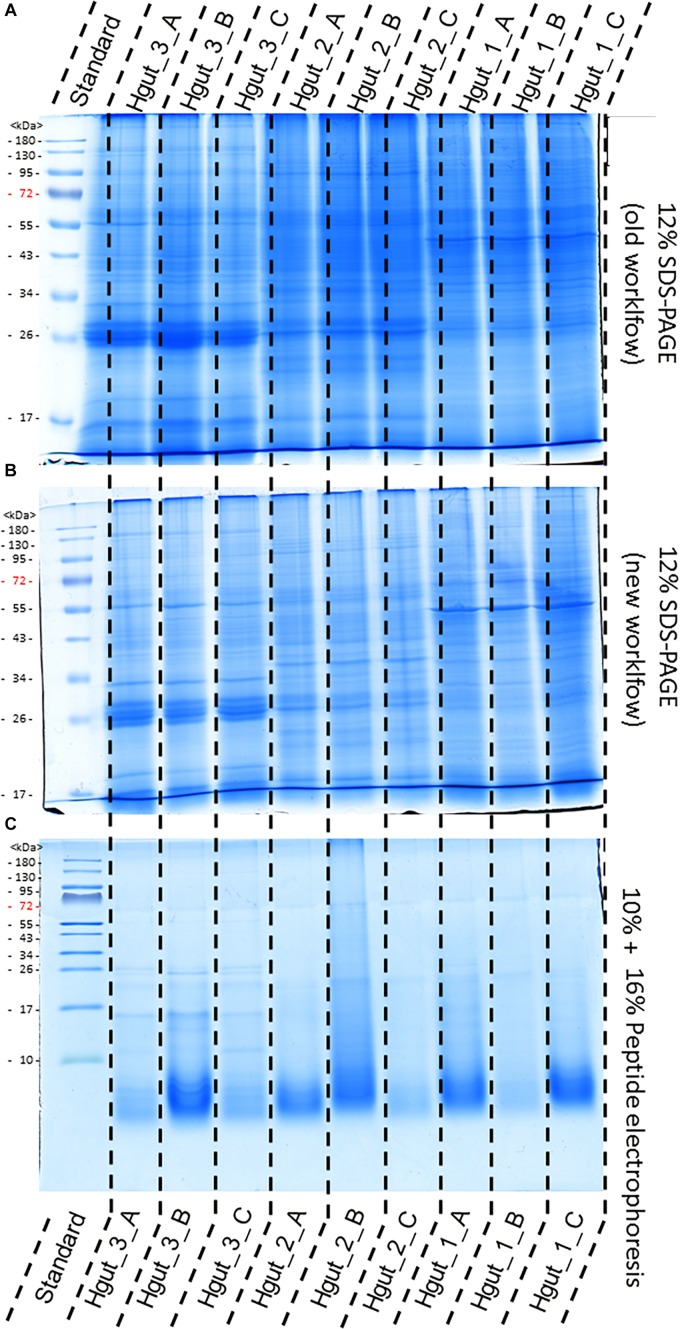
Comparison of protein extraction of human gut samples of new and old workflow. For protein separation a 12% SDS-PAGE with 1 mm gel thickness was carried out and stained with colloidal coomassie. Proteins were extract by the old workflow **(A)** and new workflow **(B)**. Peptide electrophoresis **(C)** was carried out after FASP digest according to Schägger (2006) using a 10 and a 16% acrylamide gel. (STD) molecular weight standard; (Hgut 1–3) 100 μg of human fecal sample 1–3 resp. 90 μg for peptide electrophoresis Quality and purity of protein extracts was examined by SDS-PAGE (Supplementary Presentation S1).

The old and the new sample preparation protocols resulted in a similar band pattern for every given sample, suggesting successful protein extraction in all cases (Figure 4). However, different intensities of the lanes indicated differences in the purity and quantity of the protein extracts. Protein extracts from human feces, WWTP and soil showed higher intensities than protein extracts from the BGP and compost (Supplementary Presentation S1). Peptide electrophoresis after FASP digestion yielded complete proteolysis of proteins and showed comparable intensities of peptides for most samples, indicating successful FASP digestion. Furthermore, performing peptide electrophoresis post-FASP digestion could enable researchers to identify problems that might occur during the digestion step. For example, the peptide electrophoresis of sample Hgut 3B showed protein bands at molecular weight of more than 10 kDa indicating incomplete digestion. The increase of the trypsin to protein ratio should be considered for samples of this type.

#### Validation of Protein Identification

**FIGURE 5:**
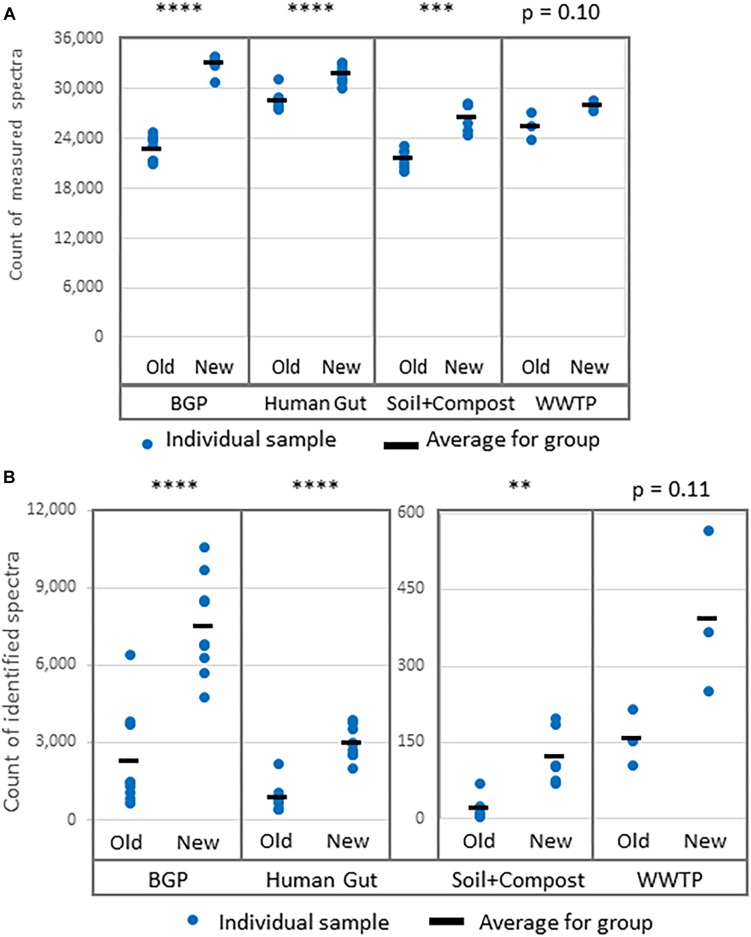
Increase of **(A)** measured spectra and **(B)** identified spectra using the new workflow of sample preparation compared against the old workflow. The data was analyzed with MPA v2. The four types of samples from BGP, human gut, soil, and compost, and WWTP show significant differences regarding spectral counts for old and new workflow (*p*-values of *t*-test are shown in the figure). Similar results were obtained for identified peptides, percentage of identified spectra or identified metaproteins (Supplementary Table S8). *P*-values: ^∗^*p* = 0.05, ^∗∗^*p* = 0.01, ^∗∗∗^*p* = 0.001, ^∗∗∗∗^*p* = 0.0001.

Comparative LC-MS/MS measurements resulted in more identified spectra for the new extraction workflow (Figure 5B). For some soil samples extracted with the old workflow, no proteins with FDR 1% were identified. To allow comparison of search results of both workflows, an FDR of 5% was applied for all soil samples although this strategy is questionable regarding the correctness of identifications. The significant increase for BGP, Hgut and soil was related to a higher percentage of identified spectra from accumulated spectra indicating a higher quality of extraction of the new workflow (Figure 5 and Supplementary Table S8). No significant increase was observed for WWTP. In addition, higher numbers of spectra were measured (Figure 5A). Probably, the FASP workflow was more efficient or removed more contaminants allowing the measurement of more and qualitatively better spectra. Numerous washing steps before digestion removed low molecular weight contaminants more efficiently. Furthermore, high molecular weight contaminants remained in the retentate while collecting the peptides in the filtrate. Skipping lyophilization after FASP and direct injection of acidified eluate had no negative impact on the number of identified spectra (Supplementary Data Sheet S2 and Supplementary Table S8). Peptide and metaprotein counts followed the same trend as identified spectra. Furthermore, this increase in identifications was independent of the MPA version used (see Supplementary Table S8).

**FIGURE 6:**
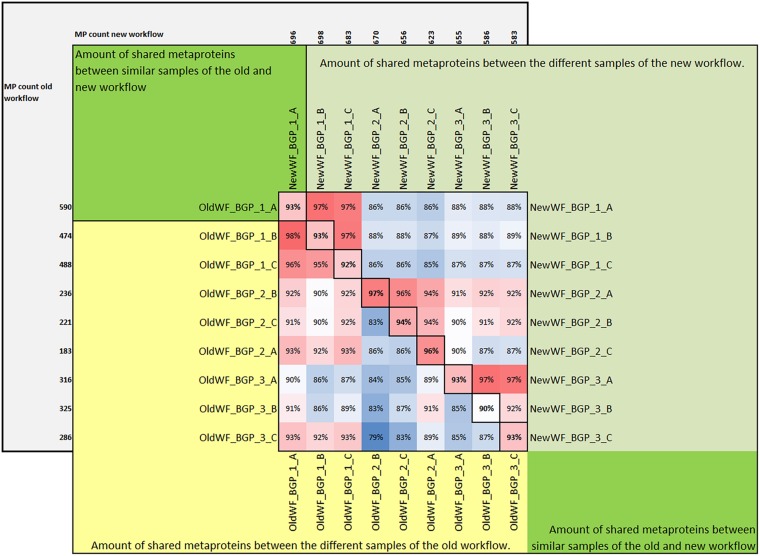
Amount of shared metaproteins between the old and new workflow. The upper triangular matrix shows the amount of shared metaproteins of the different BGP samples using the new workflow. The lower triangular matrix shows the amount of shared metaproteins of the different BGP samples using the old workflow. The diagonal shows the amount of shared metaproteins of the same sample analyzed by the old and the new workflow. For the calculation of the amount of shared metaproteins, the number of shared metaproteins was divided by the smaller number of metaproteins from both samples. For this analysis only metaproteins were considered which had in at least one sample a spectral count of 4. MP, metaprotein.

**FIGURE 7:**
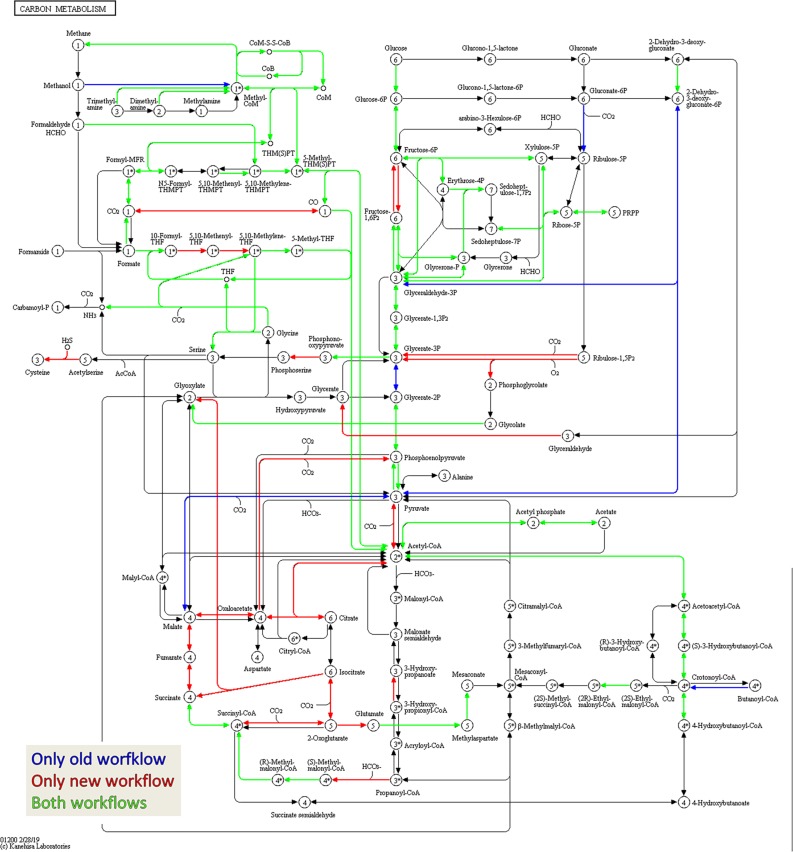
Amount of shared metaproteins between the old and new workflow. KEGG map for the carbon metabolism showing enzymes in the sample BGP_1 (three technical replicates combined, analyzed with MPAv2.12). The map is colored to highlight differences between functional annotation, where blue are KO numbers exclusively found in the analysis with old workflow, red are KO numbers exclusively found in the analysis with the new workflow and green are KO numbers found with both. The maps are also hosted on: http://www.mpa.ovgu.de/review/kegg_carbonmetabolism_BGP_1.png.

**FIGURE 8:**
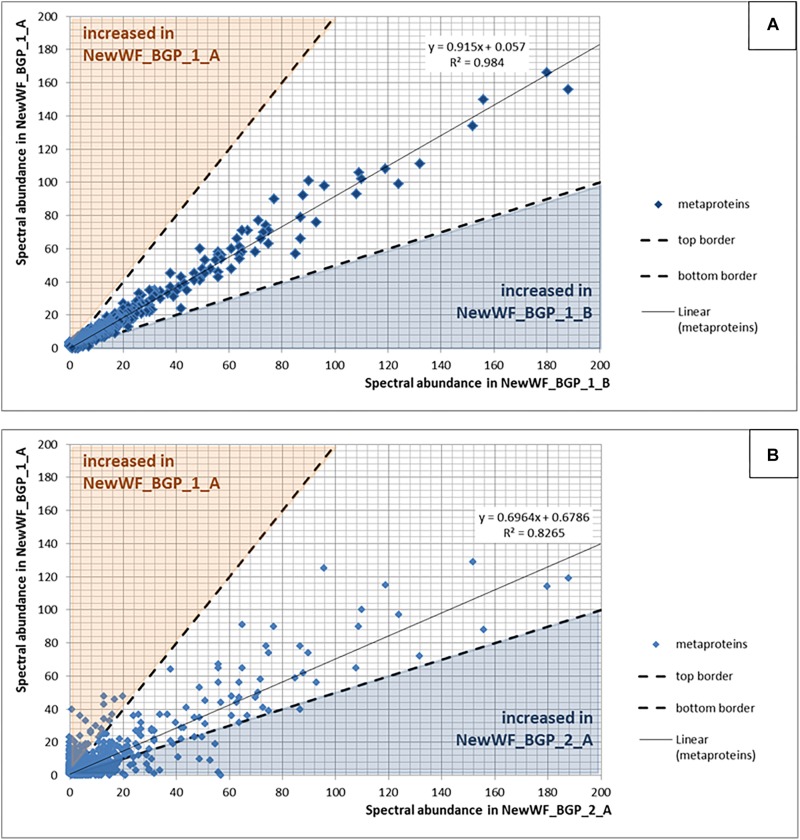
**(A)** Reproducibility using replicated samples. The spectral counts of the metaproteins from the sample NewWF_BGP_1_A were plotted against the spectral counts of the metaproteins from the sample NewWF_BGP_1_B. The points in the blue or the orange area are at least doubled in the corresponding sample. **(B)** Differences between samples. The spectral counts of the metaproteins from the sample NewWF_BGP_1_A were plotted against the spectral counts of the metaproteins from the sample NewWF_BGP_2_A. The points in the blue or the orange area are at least decreased (blue) or increased (orange) twofold.

For qualitative evaluation of the new workflow, taxonomy and function were assigned to identified metaproteins of a BGP 1A to C (using the advanced feature of MPAv2.12). Although some function were detected with the old workflow only, the new workflow showed a higher coverage of metabolic pathways in KEGG map 1200 (Figure 6 and Supplementary Table S10). The Krona plots of both samples showed minor differences in the taxonomy profile only (Supplementary Table S10). The abundances of orders varied about 1% between old and new workflow. Some minor orders were not shown either for the new or the old workflow due to limitations of this visualization. For further validation of the new laboratory workflow, pairwise Pearson correlation coefficients (Supplementary Table S6) based on the abundance of metaproteins and the percentage of identical metaproteins (Figure 7) for all pairs of samples and workflow were calculated. Both figures showed the same trends: (i) replicates of one sample were most similar (more than 90% identical metaproteins, Pearson coefficients higher than 0.9), (ii) different groups of samples were clearly separated (less than 70% identical metaproteins, Pearson coefficients lower than 0.7), (iii) identical samples prepared with the old and the new workflow showed also high similarity (more than 90% identical metaproteins, Pearson coefficients higher than 0.8), and (iv) sample groups with overall lower number of metaproteins (soil, WWTP) show heterogeneous results. These values are in the range of the observed reproducibility (70% identical proteins) of technically replicated LC-MS runs for protein identification (Tabb et al., 2010). For further validation of the reproducibility, spectral counts of identified metaproteins were compared between the two replicates of sample NewWF BGP_1. The scatterplot showed a good correlation between both replicates (Figure 8 and Supplementary Table S14). No changes in abundances (more than twofold) were detected for metaprotein present with at least 10 spectral counts in one of the replicates. In contrast the comparison of the samples NewWF_BGP_1_A and NewWF_BGP_2_A showed 116 metaproteines (present with at least 10 spectral counts in one of the replicates) with more than twofold changes in abundance that could be related to differences in the microbial community of both samples.

### Improvements of the Bioinformatic Workflow

#### BLAST of Metagenomes for Better Protein Annotation

The upgraded MPA integrates a convenient fully automated protein BLAST for user defined metagenomes. It gives the user the choice to use multiple BLAST hits and to combine them into a single entry, if they have the same *e*-value, sequence identity or bit score. A common entry uses the common ancestor taxonomy, chooses the common UniRef clusters and combines different ontologies, EC-numbers, KO-numbers between BLAST hits.

**FIGURE 9:**
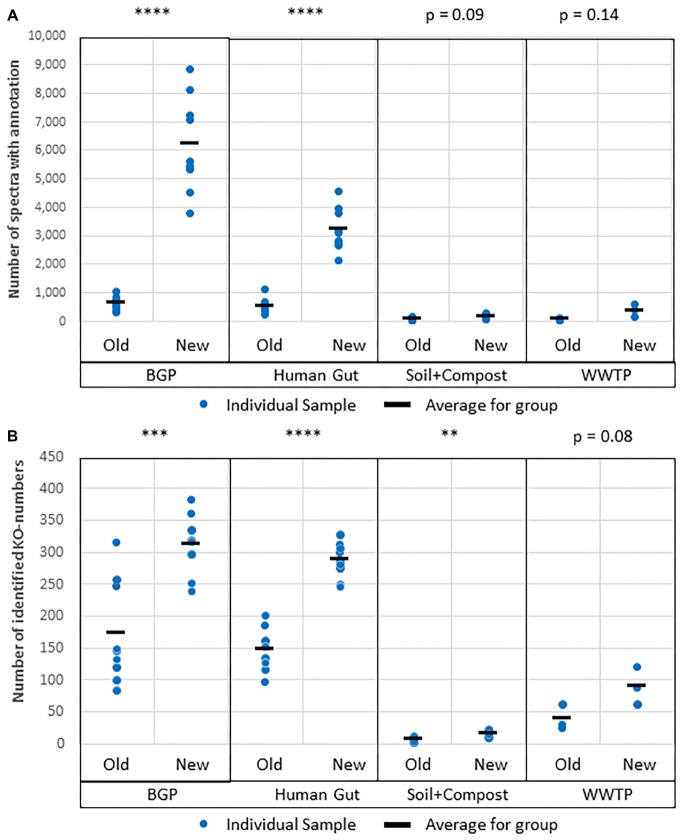
Improved protein annotation via BLAST using MPAv2 in comparison to MPAv1. **(A)** Increase of annotated spectra. **(B)** Identified KO-numbers. Significance values calculated by Student’s *t*-test for differences between the old and the new workflow are shown above the plots. The comparison was carried out with data obtained with the new laboratory workflow. The samples BGP, human gut, soil, and compost, and WWTP as well as their averages (black line) are shown separately. For further detail see Supplementary Table S15. *P*-values: ^∗^*p* = 0.05, ^∗∗^*p* = 0.01, ^∗∗∗^*p* = 0.001, ^∗∗∗∗^*p* = 0.0001.

The protein databases used for protein identification consisted of UniProtKB/SwissProt combined with an appropriate metagenome for the four sample types (Schluter et al., 2008; Qin et al., 2010; Albertsen et al., 2012; Rademacher et al., 2012; Hanreich et al., 2013). MPAv1 did not support the integrated BLAST resulting in lower numbers of annotated proteins. For the BGP, and Hgut, the portion of annotated proteins was doubled applying the integrated BLAST of MPAv2 (Figure 9 and Supplementary Table S11). For soil, and WWTP, the increase was not significant. The increase of annotated proteins was also reflected in the increase in the number of assigned KO numbers allowing better reconstruction of metabolic pathways or cellular functions. The low increase for soil and compost was related to the small size of soil metagenome supplementing UniProtKB/SwissProt.

#### Effect of Peptide Database Lookup for Metaprotein Generation

The new MPA version creates an index peptide database (since version 1.12) for uploaded protein databases (FASTA format). After database searches are finished, a lookup in this peptide index collects all proteins that contain the identified peptides. This strategy works in conjunction with the metaprotein generation, which aims to accurately represent homologous proteins across multiple species.

**FIGURE 10:**
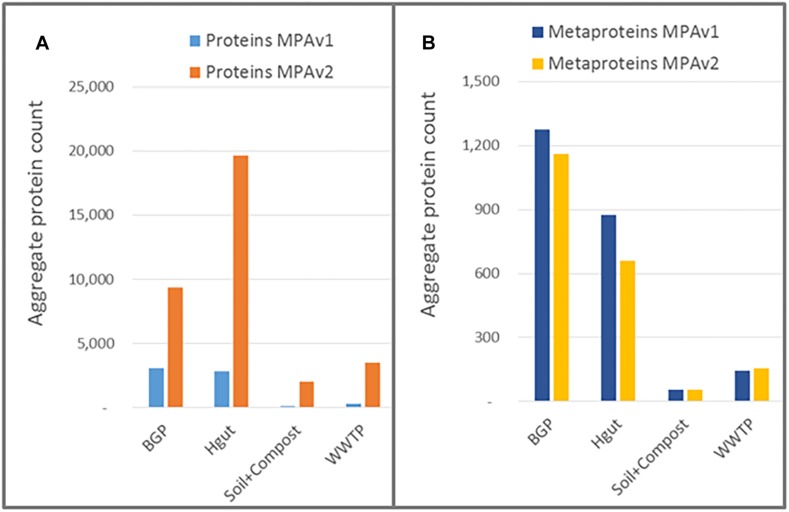
Impact of peptide database lookup on reported proteins **(A)** and metaproteins **(B)** for MPAv1 and MPAv2. The comparison was carried out with data obtained with the new laboratory workflow. The bars represent the accumulated number of proteins/metaproteins for each sample group.

The result of using the peptide database lookup in the new MPA version was an increase of reported proteins by a factor of up to 16, while the number of reported metaproteins remained approximately the same or slightly decreased (Figure 10 and Supplementary Tables S12, S13). This was in line with expectations: since no new PSMs were added, the number of identified metaproteins should remained equal.

The integration of a peptide database lookup increased the ambiguity of metaprotein annotations, in particular the taxonomy. If more proteins were grouped together into a single metaprotein, the taxonomic specificity decreased applying shared peptides for metaprotein calculation and the lowest common ancestor for taxonomic assignment (Muth et al., 2015a; for further options regarding metaprotein generation see Supplementary Table S2). This negative effect was counteracted by increased number of protein annotations from BLAST (Figure 9) providing taxonomic annotations of previously non-annotated metaproteins.

#### Compare Function for Fast Quantitative Analysis of Multiple Datasets

Another feature of the new MPA is the sample comparison function, which allows a quantitative comparison between metaproteins, peptides, taxonomies, and functional ontologies for large number of samples (highest number so far: 200). A comparison between multiple samples at the protein or peptide level is straightforward, since the protein accession or peptide sequence serve as unique identifiers. This is more complicated for metaproteins, taxonomies and functional ontologies, because these more abstract groupings are highly variable and dependent on the underlying data. For instance, using the shared peptide rule for metaprotein generation, a metaprotein will only be created if one peptide belongs to two proteins. If this shared peptide is absent in sample A, but present in sample B, sample A will contain two metaproteins and sample B will contain only one metaprotein, distorting a quantitative comparison. Therefore, the new sample comparison function of the MPA performs the metaprotein generation over any number of samples, enabling an accurate comparison of different experiments (for details regarding metaprotein generation see Supplementary Table S2).

**FIGURE 11:**
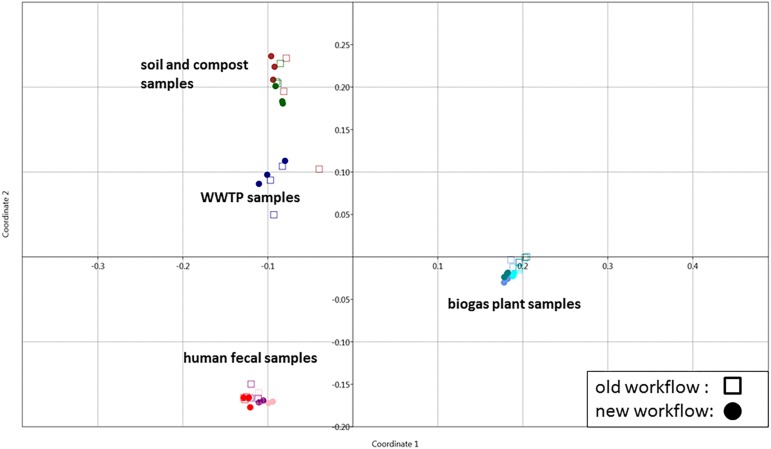
Grouping of samples using PCoA. Principle coordinate analysis of all samples extracted with the previous (square) and the new (dots) workflow using the Past 3 tool and the Bray–Curtis distance as parameter. For analysis, all metaproteins that represented at least one percent of the identified spectra in at least one sample were considered. The samples comprise the three BGP samples 1–3 (aqua, cornflower blue, teal), the three human fecal samples 1–3 (light pink, purple, red), the WWTP samples (navy), the soil sample (brown) and the compost sample (dark green).

**FIGURE 12:**
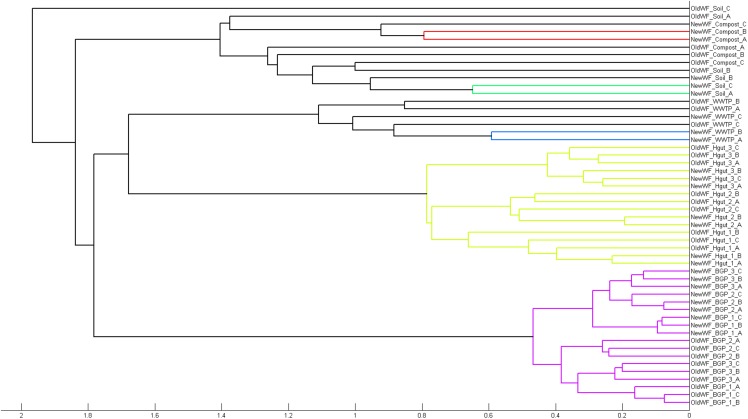
Separation of samples in cluster tree. Cluster analysis of all samples extracted with the previous and the new workflow using Matlab and the “cityblock” distance and the “average” linkage as parameter was carried out. For analysis, all metaproteins that represented at least one percent of the identified spectra in at least one sample were considered. The samples comprise the three BGP samples, the three human fecal samples 1–3, the WWTP samples, the soil sample, and the compost sample.

To demonstrate its functionality, we compared all 54 samples on the metaprotein level using the spectral count of a metaprotein as quantitative measure. The comparison table of MPAv2 (Supplementary Table S6) was exported as a comma separated value file and used as direct input for a PCoA (Figure 11). A clear separation between the human fecal samples, the BGP samples and the soil, compost and WWTP samples was visible. The quality of grouping the technical replicates seemed to depend on the sample types. On the one hand, the observed scattering of replicates was related to the quality of data. WWTP and soil samples with low numbers of identifications showed a higher scattering than BGP and human gut samples. The higher scattering in PCoA was also related to higher distances in the clustering (Figure 12). On the other hand, the scattering of samples with high quality (human gut, BGP) visualized the error of replicates (low distances in the clustering).

#### Chord Diagrams for Visualization of the Relation Between Taxonomy and Function

**FIGURE 13:**
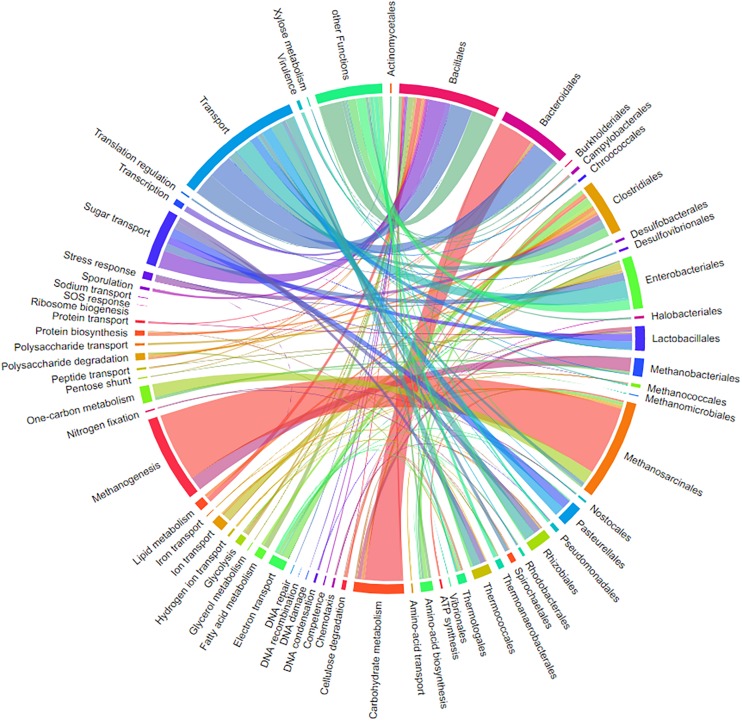
Chord-diagram visualizing the taxonomy-function-relationships for samples BGP 1A–C. Data was exported from MPA. All taxonomies except bacterial and archaeal orders were removed in the diagram (chord diagram for a Hgut sample is found in Supplementary Table S10).

One major question in microbiome research is how taxonomy is linked to function. Metaproteome data contains both levels of information. The previously published tool for connecting both levels into a single interactive figure (Zoun et al., 2017) is supported by a special export function of MPAv2 (Figure 13). The interactive figure can be adapted to the requirements by simply switching on and off certain taxonomies and functions allowing fast visualization of taxonomy-function-relationships according to user requirements (Figure 13 and Supplementary Table S10). This new export supplemented other valuable visualizations available for MPA users internally (pie charts) and externally (KEGG maps, Krona plot).

## Discussion

In this study, we proposed and evaluated a new robust and fast workflow for metaproteomics of microbial community samples for routine application. The advantages over the previous workflow (Heyer et al., 2013; Muth et al., 2015a) included performance improvements in both sample preparation and bioinformatics data processing. The objectives of our new protocols were speed, simplicity, high throughput, reproducibility, and robustness.

### Advantages of the New Laboratory Workflow

The new laboratory workflow combined phenol extraction (Heyer et al., 2013), FASP (Wisniewski et al., 2009) and LC-MS/MS measurement (Link et al., 1999). Phenol extraction combined with cell lysis in a ball mill was previously applied to numerous environmental samples (Jia et al., 2017; Thorn et al., 2018; Heyer et al., 2019). For simplicity and robustness, the new workflow omitted sophisticated and time-consuming enrichment of biomass from environmental matrices by centrifugation or filtration (Xiong et al., 2015). Furthermore, fractionation, which was frequently applied in sample preparation (Hinzke et al., 2019), was sacrificed for speed of the final workflow. The final workflow enabled an investigation with a throughput of up to 5 samples in only 24 h, only limited by the throughput of the MS. The throughput could be improved even further by parallel sample preparation in micro titer plates (Switzar et al., 2013), or the use of more mass spectrometers.

The evaluation of the new workflow confirmed that FASP digestion increased the number of identifications by at least a factor of two in comparison to the well-established in gel digestion protocol (Shevchenko et al., 2006). The most probable explanation for this large difference was a decreased efficiency of trypsin in in-gel digestion, because proteins inside the gel matrix were partially inaccessible to trypsin or the recovery of peptides from the gel matrix was poor (Leon et al., 2013). Furthermore, FASP was considered to remove contaminations: (i) low molecular weight contaminations were removed by filtrations before digestion and (ii) high molecular weight contaminations remained in the retentate after digestion. However, the number of identifications was heavily dependent on the sample type. First, a literature comparison (Supplementary Table S9) confirmed that soil metaproteome studies (Keiblinger et al., 2012; Bagnoud et al., 2016; Bastida et al., 2016; Thorn et al., 2018) identified less proteins and peptides than studies of Hgut (Tanca et al., 2016; Brown et al., 2018; Zhang et al., 2018a; Rechenberger et al., 2019) and BGP (Bize et al., 2015; Hagen et al., 2017; Joyce et al., 2018). Second, it became obvious that sacrificing the fractionation before or after (Hinzke et al., 2019) tryptic digestion resulted in lower number of identifications. Considering the speed for measuring the samples without fractionation, the number of identified proteins was still competitive in most cases, for BGPs even better. Despite the increased efficiency achieved with the new FASP protocol, the number of identifications was still influenced strongly by the sample type. Poor protein abundance could be overcome by collecting higher sample volumes and pooling of multiple extracts of the same sample. When a higher metaproteome coverage is required to derive meaningful results for more scientific projects, supplementary fractionation techniques such as isoelectric focusing (Kohrs et al., 2014) or ion exchange chromatography (Erickson et al., 2012; Kleiner et al., 2017) could be applied. However, these solutions would come at the expense of throughput. Since low protein abundance and poor extraction from sample matrices might occur with any new sample, the recommended strategy for new samples is to control the quality of extraction and digestion using SDS-PAGE and peptide electrophoresis beforehand.

The reproducibility of the workflow was demonstrated by high numbers of identical metaproteins and high Pearson correlation coefficients for replicated samples or for sample types. Considering the number of identical metaproteins, the reproducibility cannot exceed the limits of replicated LC-MS/MS measurements for protein identification (Tabb et al., 2010). High reproducibility was confirmed further by similar spectral counts for identified metaproteins of two technical replicates of a BGP sample, whereas the quantitative comparison of two different BGP samples revealed numerous metaproteins with different abundance.

Robustness of the workflow was related to repeated assignment of replicates to each other using statistical data analysis. Grouping of replicates and separation of different sample types was observed by PCoA and clustering. Therefore, single replicates appeared to be sufficient for future studies. The specificity of the workflow should enable the separation of different samples as shown for BGP and Hgut (different patients). For soil and WWTP, reproducibility and robustness were lower due to low numbers of identified metaproteins. These results indicated that at least several hundred metaproteins are required for statistical data analysis.

### Advantages of the New MPA

Another focus of this study was the improvement of the bioinformatics workflow by further development of the MPA software. Several tools for metaproteomics are available and provide valuable problem-specific solutions (e.g., Prophane, iMetaLab 1.0, UniPept) (Schneider et al., 2011; Cheng et al., 2017; Mesuere et al., 2018). None of these tools, however, offers the user a full workflow beginning with MS data and ending with protein reports and visualizations. Major advantages of the previous MPA were the dynamic metaprotein generation and the flexibility in taxonomic as well as functional filtering.

In contrast to the recently published MPA Portable (Muth et al., 2018), which fits well into a research context, where data science experts and computing resources are more easily available, the MPA 2.12 enables users with little or no background in computer science to conduct metaproteomics experiments with ease. While both options – local deployment or central solution – are available to users, central solutions (Cheng et al., 2017; Afgan et al., 2018; Liao et al., 2018) can keep up with the ever increasing data generated by high-throughput MS and the associated computational demands for broad application in routine analyses.

The newly implemented peptide database lookup and the integrated protein BLAST doubled the number of metaproteins annotated on the taxonomic and functional level. Together with the previously implemented metaprotein generation, the MPA now provides a unique workflow of functions that are available separately by other tools, e.g., Unipept or Prophane. The unique workflow within a single software speeds up the data analysis by omitting the file-based transfer of data between different tools. For further improvement, binned metagenomes containing taxonomic and functional data of high quality (Junemann et al., 2017) could be used. Assignment of metaproteins to genome bins would allow a more specific reconstruction of metabolic pathways based on additional information from the context of the genome bin. Furthermore, the concatenation of metagenomes from a similar sample and UniProtKB/SwissProt could improve the identification rate even more (Heyer et al., 2016). In addition, metapeptide databases based on raw metagenomes have been shown to increase protein identification too (May et al., 2016). The issue of correct selection of databases requires attention of users but is discussed elsewhere (Muth et al., 2015b; Timmins-Schiffman et al., 2017; Schiebenhoefer et al., 2019).

Building on these strengths, the new quantitative comparison function provides an overall metaprotein generation unifying single datasets for final export into other software. The exported CSV-files allowed a fast subsequent analysis of multiple sample data with Excel, MatLab, Past3 or R. The simple and fast combination of multiple datasets by MPA is a precondition for quantitative and statistical analysis of data from high-throughput-studies. It needs to be mentioned that due to the application of multiple search engines more than one peptide could be assigned to a spectrum. Due to high mass accuracy of precursor spectra with orbitrap instruments this ambiguity is a very rare event. Therefore, it was decided to keep both results when developing the first version of MPA. The minor risk of failures in counting should be considered for diagnostic applications. We strongly suggest the validation of potential markers peptides and quantification based on multiple peptides.

In addition, the chord diagram is a smart interactive tool visualizing the relation between taxonomy and functions that could be used for primary exploration of data or for preparing interactive visualization of data for publications.

### Steps Toward the Application of Metaproteomics in Applied Research and Diagnostics

The new metaproteomic workflow was substantially improved regarding speed, throughput and simplicity. Reproducibility, and robustness were shown by statistical analysis of the provided data. In contrast to these strengths, its resolution was limited due to sacrificing additional fractionation steps in sample preparation. However, it could be easily upgraded for fundamental science by adding fractionation on the peptide level (e.g., MudPIT; Schirmer et al., 2003), at the expense of speed. Next steps for its application in applied research and diagnostics are: (i) validation using more samples, (ii) further exploration of its strengths and limitations, and (iii) approval of its sensitivity and specificity in real projects from researchers in biotechnology and medicine.

Related to the exploration of strengths and limitations, the depth of data required for valuable data analysis needs to be considered. Instead of deep exploration of microbiomes by achieving as many identifications as possible, proteotyping of microbial communities (Heyer et al., 2016; Kohrs et al., 2017) aims to detect single marker proteins or process (disease) specific protein signatures. It is questionable, whether metaproteins are the preferred level of data. Metaproteins contain a high level of information (taxonomy and function), but merging peptides of multiple proteins could hinder correlations with patient/process data. Therefore, single peptides should also be correlated to the state of the samples. Based on such results, multiple reaction monitoring (Yao et al., 2013) could be applied as a more specific and more quantitative approach for diagnostic applications. Furthermore, the specificity of selected marker peptides needs to be crosschecked by bioinformatic analysis (e.g., the tryptic peptide analysis of Unipept 4.0; Mesuere et al., 2018)^2^. However, Unipept is based on UniProt database and does probably not contain all peptides detected in the samples.

The main dilemma is that further development and validation of the workflow for diagnosis requires its extensive application producing comprehensive datasets for subsequent correlation to patient/process data, but in comparison to conventional diagnostic tools the effort still appears to be very high at this stage. The samples analyzed in this paper exemplify potential applications. In order to justify further comprehensive studies, selected results are discussed referring to recent literature. Omitting extensive sample preparation enabled also the detection of “contaminating” non-microbial proteins from host (Lehmann et al., 2019) or from feed (Heyer et al., 2015) that could be valuable for understanding disease or technical processes. For instance, the disease marker calprotectin is commonly monitored in stool samples through ELISA to discriminate between inflammatory bowel syndrome and inflammatory bowel disease (Caccaro et al., 2012). Calprotectin was easily found using our metaproteomics workflow alongside many other potential disease markers of human and microbial origin (Supplementary Table S6; Lehmann et al., 2019). Whereas ELISA is restricted to a single protein and relies on antibodies that may bind unspecifically, metaproteomics can detect a multitude of protein alterations for disease specific pattern recognition and thus enable a more comprehensive and robust diagnosis. This will be particularly useful if the impact of the microbiome on certain diseases such as diabetes, several autoimmune diseases, obesity and depression is better understood and microbial marker proteins for these diseases are known. For BGP, the supporting effect of annotating hits from non-annotated metagenome data by BLAST was obvious. Key enzymes for all major pathways of anaerobic digestion were detected. The abundance of methyl-coenzyme M reductase has been identified previously as a predictive biomarker for performance of BGP (Munk et al., 2012). Whereas the suggested RT-PCR assay focussed only on a single function, metaproteome data provides additional data that discriminated between the acetoclastic and hydrogentrophic pathways of methanogenesis (Heyer et al., 2016, 2019).

## Conclusion

In conclusion, the new metaproteomics workflow presented in this study combines robust and fast sample preparation with improved data processing in a single standardized workflow. The evaluation of the workflow showed a significant increase in quality and quantity of generated results compared to our previously reported workflows. Performance and processing time provide a basis for establishing metaproteome based diagnostics in clinical settings and routine analysis of technical and environmental samples in the future. Further steps to explore the potential of the workflow are necessary and should be a major focus of future research.

## Data Availability

The raw data and the FASTA database are available for download from PRIDE (PXD010550) (Vizcaino et al., 2016).

## Ethics Statement

Fecal samples were collected from three healthy, omnivorous male subjects (A, B, and C) in the age-range of 30–33 as part of the proof-of-principle study. The study was approved by the ethical committee of the Otto von Guericke University Magdeburg (Number 99/10). All healthy volunteers provided written informed consent. The samples were stored at −20°C.

## Author Contributions

The improvement of the laboratory workflow was carried out by AnB, CS, and RH. The further development of the MPA was done by KS, RZ, RH, TM, and SD. The manuscript was written by RH, DB, KS, and UR. FK and SP tested the software and provided user feedback for development. TM, SD, AlB, and GS contributed with the valuable advice and by editing the manuscript. All authors read and approved the final manuscript.

## Conflict of Interest Statement

The authors declare that the research was conducted in the absence of any commercial or financial relationships that could be construed as a potential conflict of interest.

## Footnotes

1 www.mpa.ovgu.de

2 https://unipept.ugent.be/

## Supplementary Material

TABLE S1 Digestion time.

TABLE S2 Manual MPA.

TABLE S3 Sample metadata.

TABLE S4 Metaprotein lists.

TABLE S5 Taxonomic annotation.

TABLE S6 Comparison table and reproducibility.

TABLE S7 Protein quantification.

TABLE S8 Identifications.

TABLE S9 Literature comparison.

TABLE S10 Additional visualizations.

TABLE S11 Effect of BLAST.

TABLE S12 PeptideDB lookup.

TABLE S13 Metaprotein annotations.

TABLE S14 Calculation of scatterplots.

TABLE S15 Number of identified KOs and ECs.

DATA SHEET S1 Collection SOPs.

DATA SHEET S2 Chromatograms.

PRESENTATION S1 Quality control gels.

## Abbreviations

BGP: biogas plant
de.NBI: German Network for Bioinformatics Infrastructure
DTT: dithiothreitol
FASP: filter aided sample prep
Hgut: human gut
IAA: iodoacetamide
LC-MS/MS: liquid chromatography tandem mass spectrometer
MPA: MetaProteomeAnalyzer
MPAv1: MetaProteomeAnalyzer version 1.0.5
MPAv2: MetaProteomeAnalyzer version 2.12
MS: mass spectrometry/mass spectrometer
PCoA: principal coordinates analysis
RT: room temperature
SOP: standard operation procedure
TFA: trifluoroacetic acid
WWTP: wastewater treatment plant
