# Supplementary material for: A Robust and Universal Metaproteomics Workflow for Research Studies and Routine Diagnostics Within 24 h Using Phenol Extraction, FASP Digest, and the MetaProteomeAnalyzer
Source: Front Microbiol. 2019 Aug 16;10:1883. doi: 10.3389/fmicb.2019.01883 (PMC6707425; doi:10.3389/fmicb.2019.01883)
Supplement: TABLE S1 — Digestion time. [file Table_1.DOCX]

Evaluation of different trypsin incubation times

For in gel digestion of environmental samples the trypsin incubation time is usually overnight^1^. According to *Wiśniewski et al. (2016)*^2^ 2 h trypsin incubation is sufficient for the FASP digest. Since environmental samples contain high amounts of sample impurities the decreased trypsin incubation times were examined. Therefore, the biogas plant samples BGP 3 and BGP 7 were digested using the FASP digest with 2 h, two times 1 h, 4 h and overnight trypsin incubation. The quality of the FASP digest was validated by peptide electrophoresis and LC-MS/MS measurement with an Orbitrap Elite™ Hybrid Ion Trap-Orbitrap MS/MS (MS) (both from Thermo Fisher Scientific, Bremen, Germany). Protein database search was performed against UniProtKB/ SwissProt using Mascot and a Mascot score of 40 as threshold.

Evaluation of the peptide electrophoresis [Figure 1] showed a strong band below 10 kDa but no bands above 26 kDa. This indicated a successful tryptic digest for all investigated trypsin incubation times. In line LC-MS/MS measurement revealed between 40,096 to 42,177 measured spectra, between 1,330 to 1,990 identified spectra as well as between 659 to 912 identified proteins [Figure 2]. Overall, there might be little decrease in the number of identified spectra and proteins, reasoned maybe by increased autolysis of trypsin^3^. However, this effect was not significant.

Since this study wanted to reduce the worktime for the sample preparation it was decided to incubate with trypsin only for 2 h.


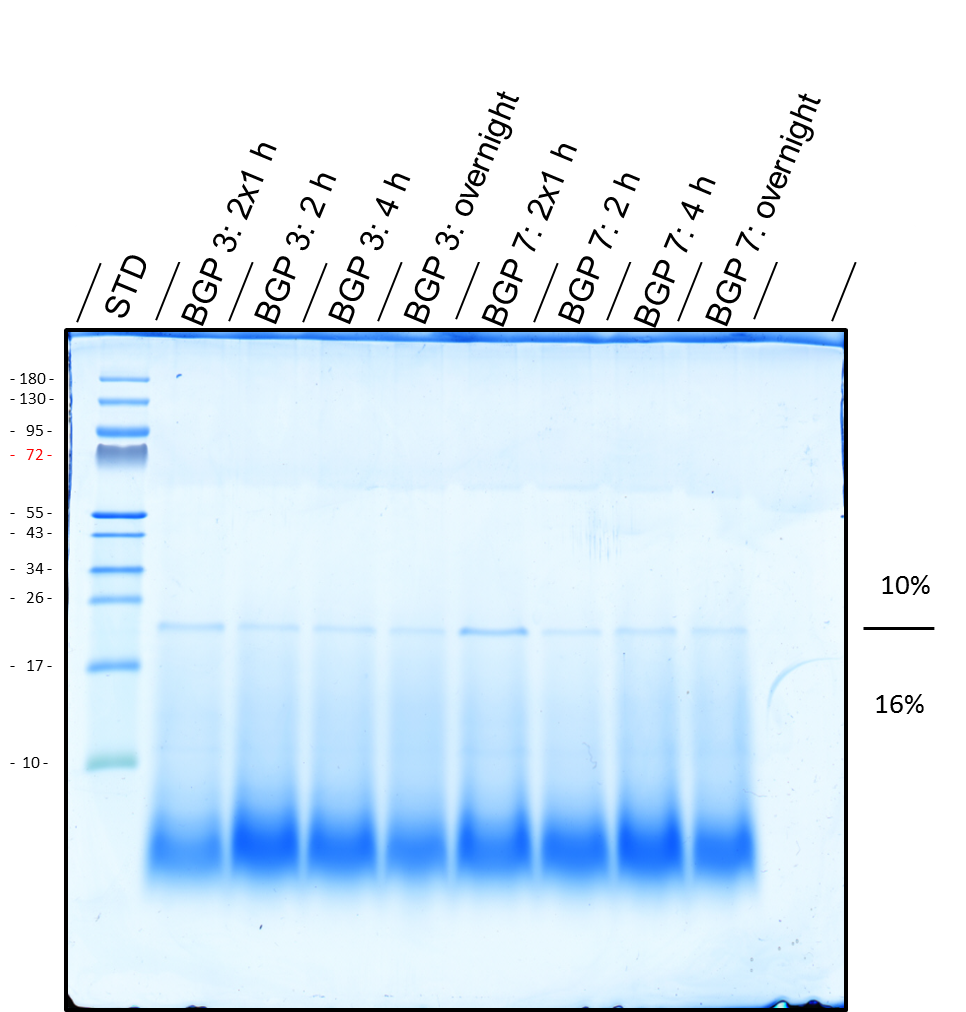


Figure 1: Impact of incubation time on the amount of digested peptide. For peptide separation a 16%/ 10% peptide electrophoresis with 1 mm gel thickness and 90 µg digested protein was carried out and stained with colloidal coomassie. Proteins extracts from BGP 3 and a further biogas plant were digested using the FASP digest. (STD) size standard;


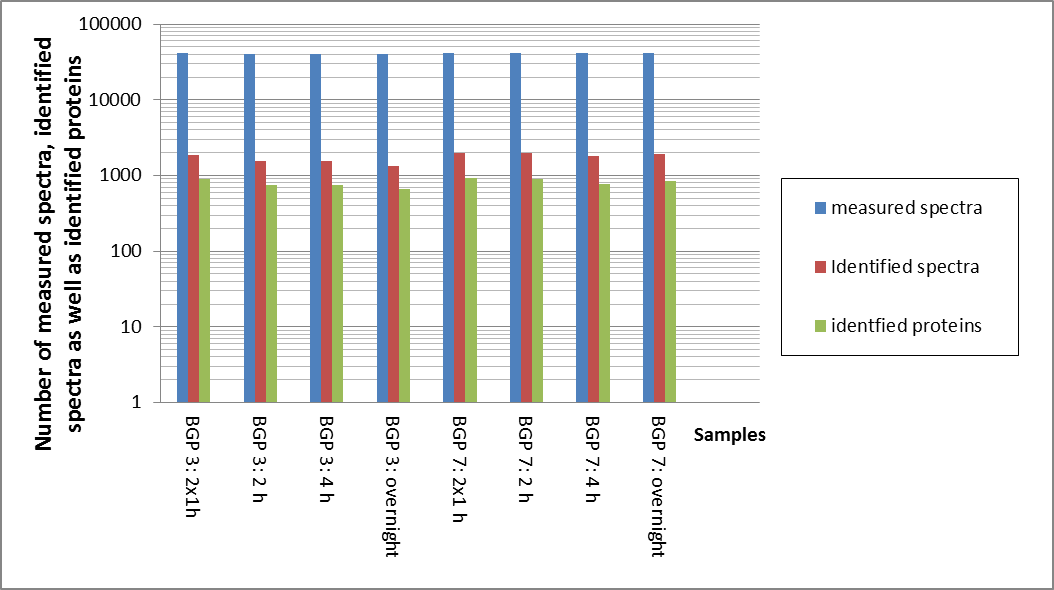


**Figure 2: Impact of incubation time on the number of identified spectra and proteins.** Samples were measured by a LC-MS/MS measurement with an Orbitrap Elite™ Hybrid Ion Trap-Orbitrap MS/MS (MS) (both from Thermo Fisher Scientific, Bremen, Germany). Protein database search was performed against UniProtKB/ SwissProt using Mascot and a Mascot score of 40 as threshold.

**References**

1. Heyer R, et al. Metaproteome analysis of the microbial communities in agricultural biogas plants. N Biotechnol 30, 614-622 (2013).

2. Wisniewski JR. Quantitative Evaluation of Filter Aided Sample Preparation (FASP) and Multienzyme Digestion FASP Protocols. Anal Chem 88, 5438-5443 (2016).

3. Hu M, Liu Y, Yu K, Liu X. Decreasing the amount of trypsin in in-gel digestion leads to diminished chemical noise and improved protein identifications. J Proteomics 109, 16-25 (2014).
