# Supplementary material for: A Robust and Universal Metaproteomics Workflow for Research Studies and Routine Diagnostics Within 24 h Using Phenol Extraction, FASP Digest, and the MetaProteomeAnalyzer
Source: Front Microbiol. 2019 Aug 16;10:1883. doi: 10.3389/fmicb.2019.01883 (PMC6707425; doi:10.3389/fmicb.2019.01883)
Supplement: TABLE S2 — Manual MPA. [file Table_2.DOCX]

**Manual for the**

**MetaProteomeAnalyzer Software (MPA)**


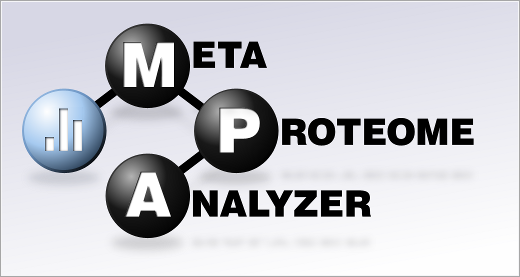


Content

[**Introduction** 3](#_Toc527469141)

[Metaproteomics analysis 3](#_Toc527469142)

[Scope of the MetaProteomeAnalyzer software 3](#_Toc527469143)

[Remote Server (www.mpa.ovgu.de) 4](#_Toc527469144)

[**Installation** 6](#_Toc527469145)

[**Overview** 7](#_Toc527469146)

[Basic Elements of the MPA 7](#_Toc527469147)

[**Workflow** 9](#_Toc527469148)

[Load FASTA database 9](#_Toc527469149)

[Select Project and Experiment 11](#_Toc527469150)

[Input Spectra Panel, protein database searches 13](#_Toc527469151)

[Fetch Results, Overview and Process Results 18](#_Toc527469152)

[Database search results view 20](#_Toc527469153)

[Annotate unknown proteins via BLAST 24](#_Toc527469154)

[Export Results 26](#_Toc527469155)

[Compare Results 29](#_Toc527469156)

[Metaprotein concept 30](#_Toc527469157)

# **Introduction**

## Metaproteomics analysis

In nature microorganisms live in complex microbial communities. Comprehensive taxonomic and functional knowledge about microbial communities supports medical and technical application such as fecal diagnosis as well as operation of biogas plants or waste water treatment plants. Furthermore, microbial communities are crucial for the global carbon and nitrogen cycle in soil and in the ocean. Among the methods available for investigation of microbial communities, metaproteomics can approximate the activity of microorganisms by investigating the protein content of a sample. Although metaproteomics is a very powerful method, issues within the bioinformatic evaluation impede its success. In particular, construction of databases for protein identification, grouping of redundant proteins as well as taxonomic and functional annotation pose big challenges. Furthermore, growing amounts of data within a metaproteomics study require dedicated algorithms and software. More information on metaproteomics data analysis can be found in the review “*Challenges and perspectives of metaproteomic data analysis*” (J Biotechnol., 2017, <https://www.sciencedirect.com/science/article/pii/S0168165617314979?via%3Dihub>)

## Scope of the MetaProteomeAnalyzer software

The MetaProteomeAnalyzer software (MPA) is an intuitive open-source tool for metaproteomics data analysis and interpretation, which includes multiple search engines and the feature to decrease data redundancy by grouping protein hits to so-called metaproteins. The MPA provides a complete pipeline from peak lists generated by the mass spectrometer software to statistical analysis of results produced by protein database search. Since the MPA was developed for metaproteomics, many features focus on taxonomic and functional analysis of the discovered proteins. The protein groups called metaproteins constitute a core functionality of the MPA to properly abstract biologically relevant information from raw results. The MPA is not applicable to specialized proteomics questions like post-translational modifications (PTM) or quantification via isotope labelling.

# Remote Server

To enable users easy access to the latest version of the MPA, software support and computing resources, the MPA is available as a server version under the URL [www.mpa.ovgu.de](http://www.mpa.ovgu.de). Using a Remote Desktop Connection, users can connect to the server use the software without having to install or update it. To get access and support to the remote server you can contact the development team via email [mpa@ovgu.de](mailto:mpa@ovgu.de).

To connect to the server, follow these instructions:

1. Establish remote Connection
   1. Open “Remote Desktop Connection” on Windows operating systems

***
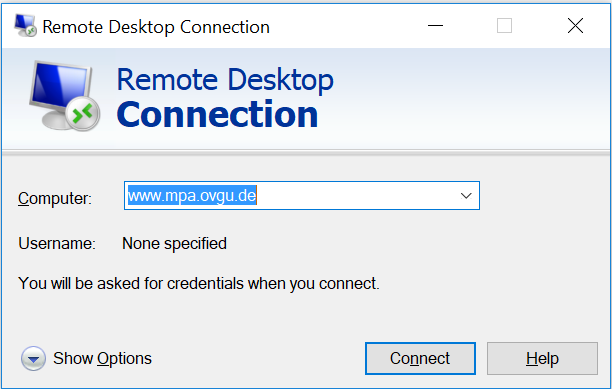
***

- 1. Use an equivalent tool for Mac or Linux operating systems

Mac
<https://docs.microsoft.com/en-us/windows-server/remote/remote-desktop-services/clients/remote-desktop-mac>

Linux
<https://www.linux.com/learn/intro-to-linux/2017/11/how-set-easy-remote-desktop-access-linux>

1. Connect to [***www.mpa.ovgu.de***](http://www.mpa.ovgu.de)
2. Enter your credentials


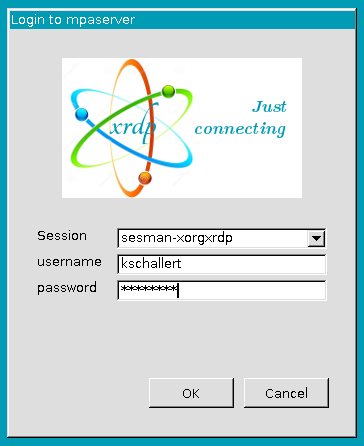


1. You can start the MPA using the “start-mpa.sh” Skript


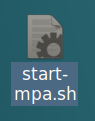
 ***
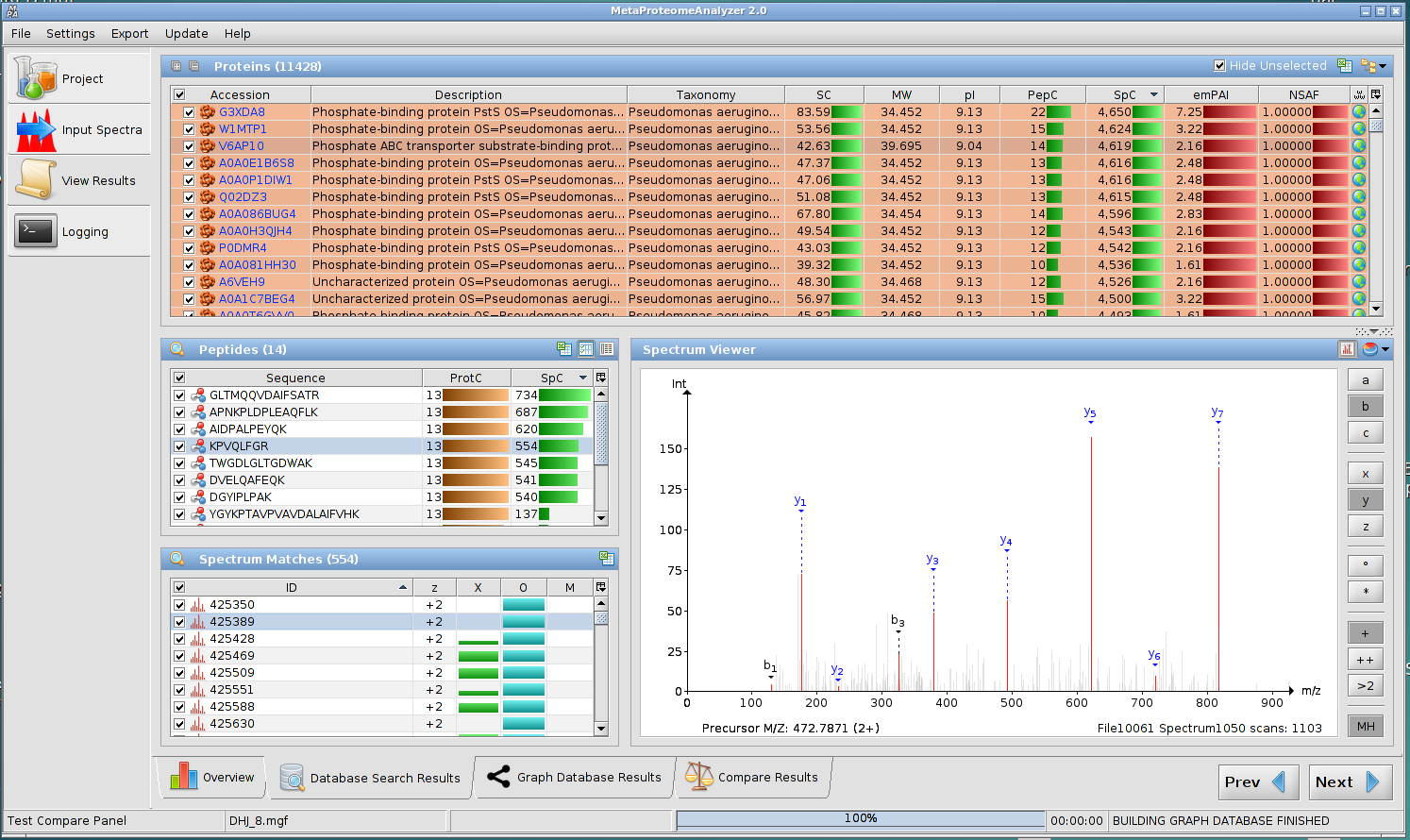
***

1. Using the X-button you can disconnect but not close a session, while the MPA is running (i.e. long searches) and connect later to continue your work
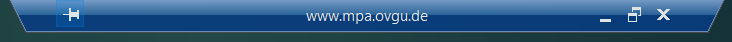

2. To properly close the session use the “Logout” option of the remote operating system


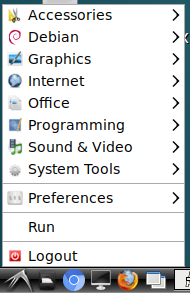

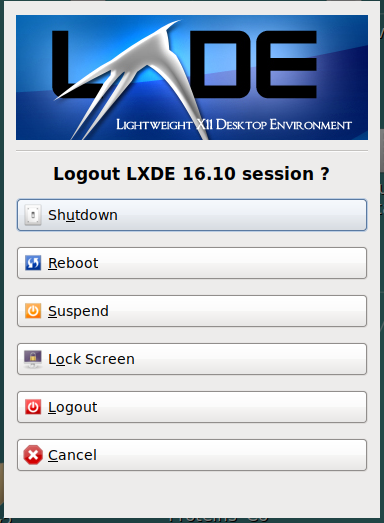


# Installation

## Windows operating systems

1. Download and unzip full package from www.mpa.ovgu.de
2. Install XAMPP and start MySQL module on localhost
3. Change the “base_path” and “xampp_path” in the "config_WINDOWS.properties" file
4. Run init/init_db_windows.bat (change the xampp dir in the script if necessary)
5. Run "MPA.bat" to start MPA software
6. Optional: Create link of the "MPA.bat" file and copy to start menu or desktop

## Linux operating systems

1. Download and unzip full package from www.mpa.ovgu.de
2. Install LAMP stack (Linux, Apache, MySQL, PHP)
3. Change the “base_path” and in the "config_LINUX.properties" file
4. Run mysql in the command line to initialize the MPA database:
5. Start the MPA using from the command line
6. Optional: Create an sh-skript to easily start the MPA without the console

# **Overview**

## Basic Elements of the MPA

The MPA graphical user interface (GUI) allows easy navigation between different tasks, that together make up the workflow of the MPA: 1. Loading and organizing spectrum data 2. Spectrum preprocessing and running protein database searches 3. Result analysis. Three panels are corresponding to these tasks and accessible using the workflow tabs or through the navigation buttons: 1. Project Tab (Figure 3), 2. Input Spectra Tab (Figure 4), 3. View Results Tab (Figure 5). Additionally, the Logging panel will display status information. The user will naturally follow the three steps in order when analyzing mass spectrometry data.

Figure 3 highlights the main components of the GUI:

1. **Menu Bar:** Contains several functions for settings, protein database handling and exports.
2. **Current Panel:** This area will show the main content of the current panel.
3. **Workflow Panels:** The workflow panels allow easy navigation between using 4 tabs for the workflow.
   1. **Project Panel:** For organization and selection of spectrum data.
   2. **Input Spectra Panel:** For loading spectrum data and starting protein database searches.
   3. **View Results Panel:** For viewing, analyzing, visualizing and further processing of results.
   4. **Logging Panel:** For more detailed feedback about the current status.
4. **Navigation Buttons:** Contains additional buttons for navigation between tabs.
5. **Status Panel:** Shows useful information about the current status.


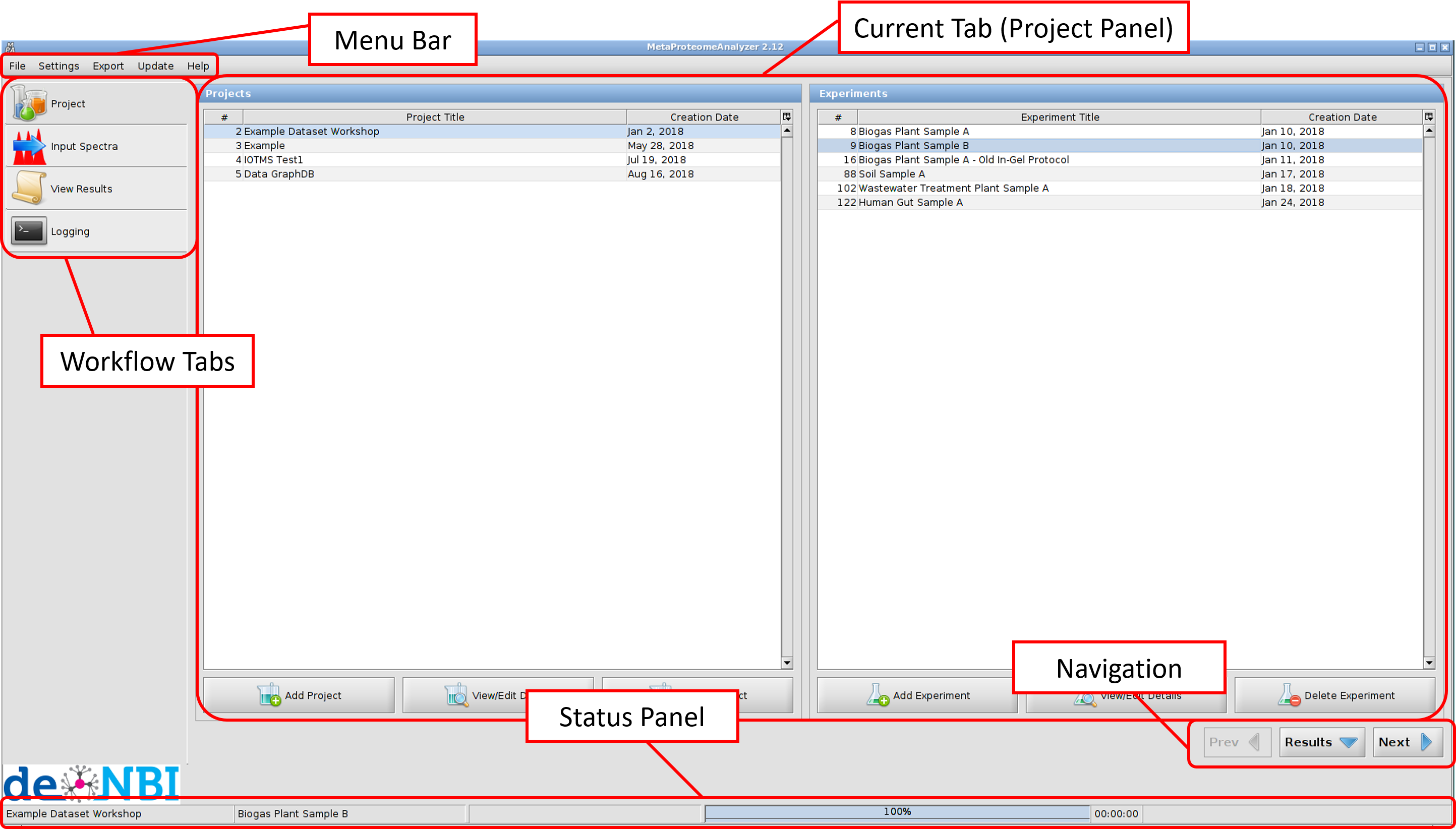


Figure 3: Main elements of the graphical user interface (GUI). The MPA will start with the Project Panel, other panels are accessible via navigation buttons or by switching workflow tabs. Certain general options are available in the menu bar. The Status Panel will display helpful information.


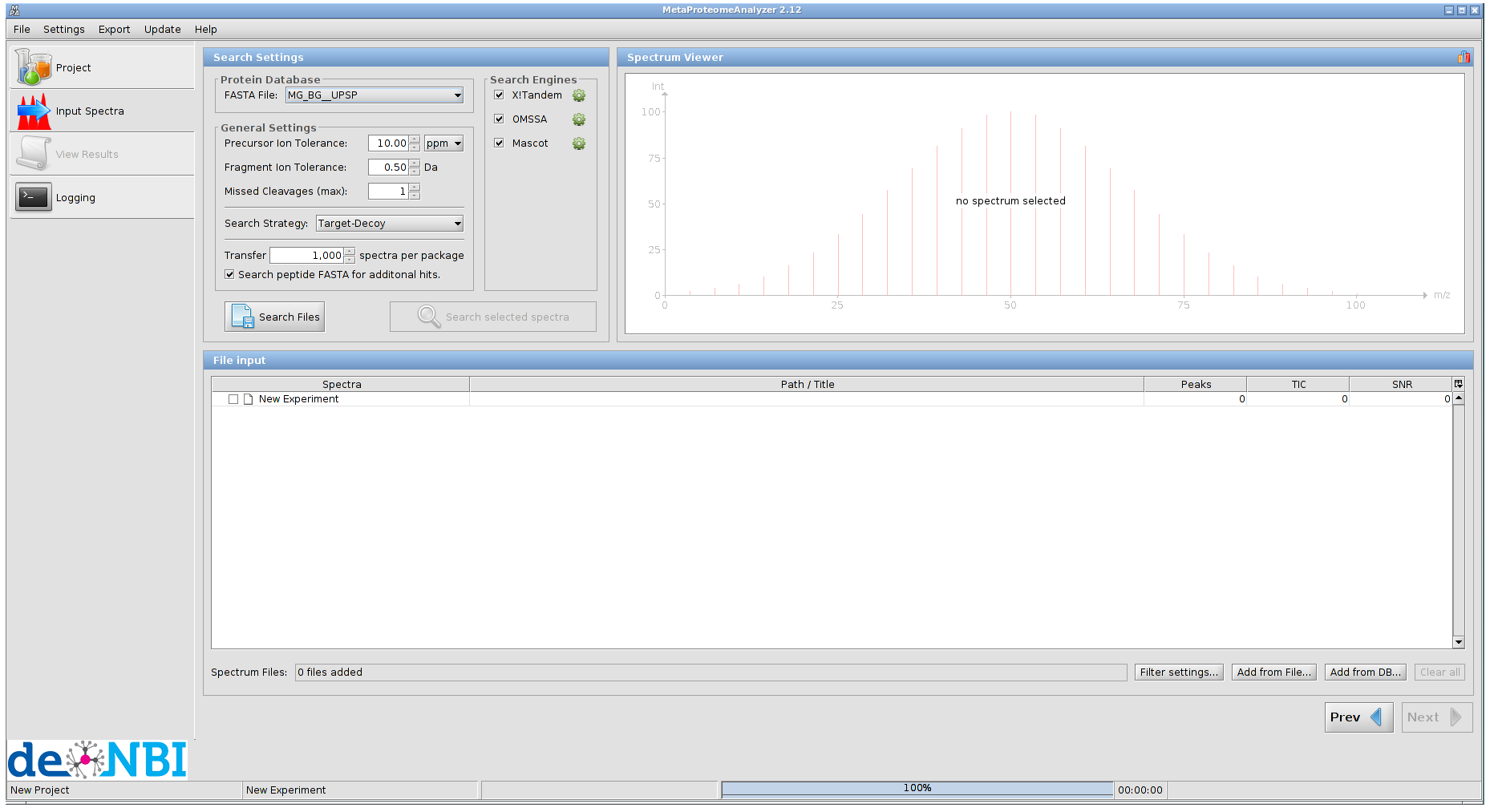


Figure 4: Input Spectra Panel. The Input Spectra panel, when opened initially. From here protein database searches can be started using either spectrum files directly or by loading spectra from files or databases and applying a preselection. Mascot search results (dat-files) can be loaded into an experiment (no search).


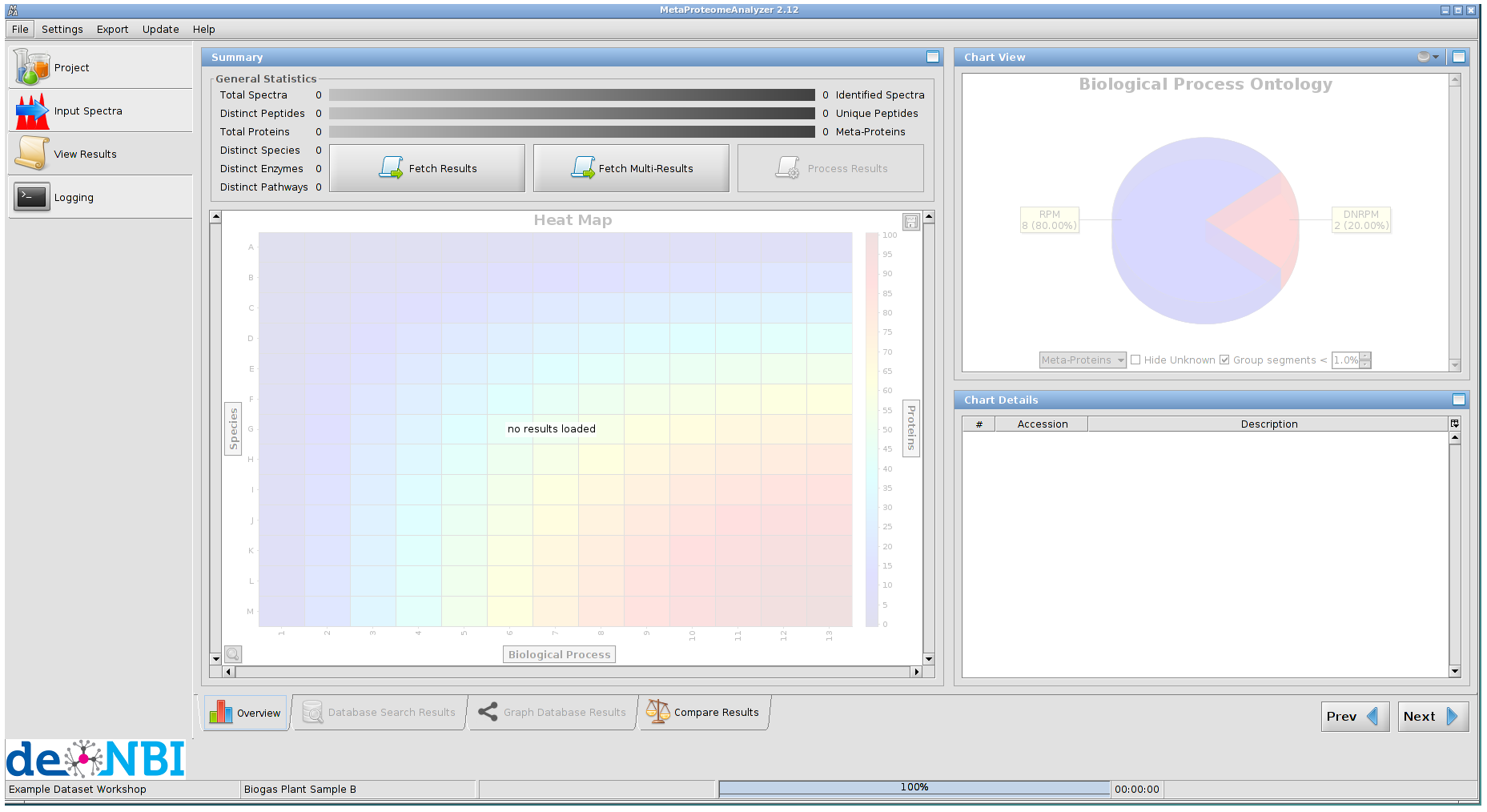


Figure 5: View Results Panel: The View Results panel, when opened initially. Results from database searches can be viewed and analyzed here. The panel is divided into four additional tabs: 1. Overview, 2. Database Search Results, 3. Graph database Results and 4. Compare Results.

# **Workflow**

## Load FASTA database

The first step is to choose a protein sequence database (i.e. an appropriate metagenome) against which the mass spectra are searched. Protein sequence databases in the form of FAA files (or FASTA) are uploaded to the MPA where they will be preprocessed and stored for the use in all future searches (see Step-by-step guide). If the FASTA file is formatted in a specific way (i.e. UniProt formatting), the MPA will recognize this and parse the data accordingly (Table X). In case of UniProt entries additional metadata (taxonomy, etc.) is retrieved as well.

Step-by-step guide:

1. In the **Menu Bar**🡪Click **Update**🡪 Click **Add Fasta database**


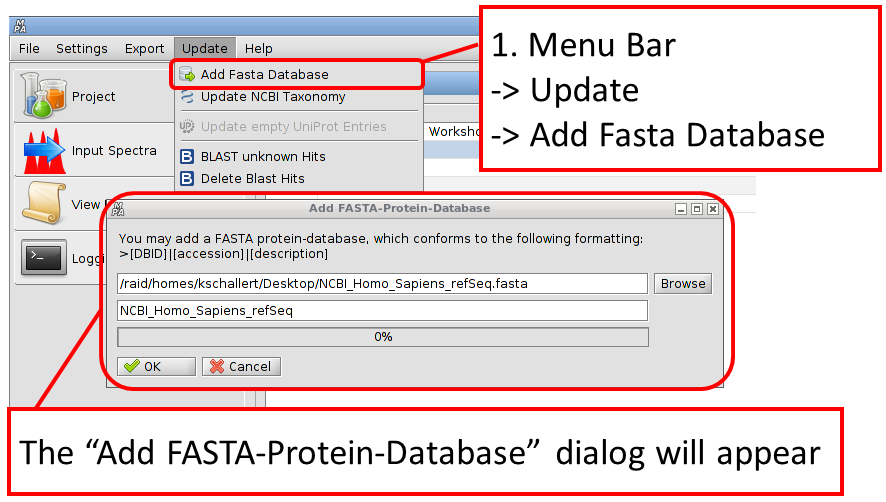


1. Click **Browse**, Select Fasta file from file system


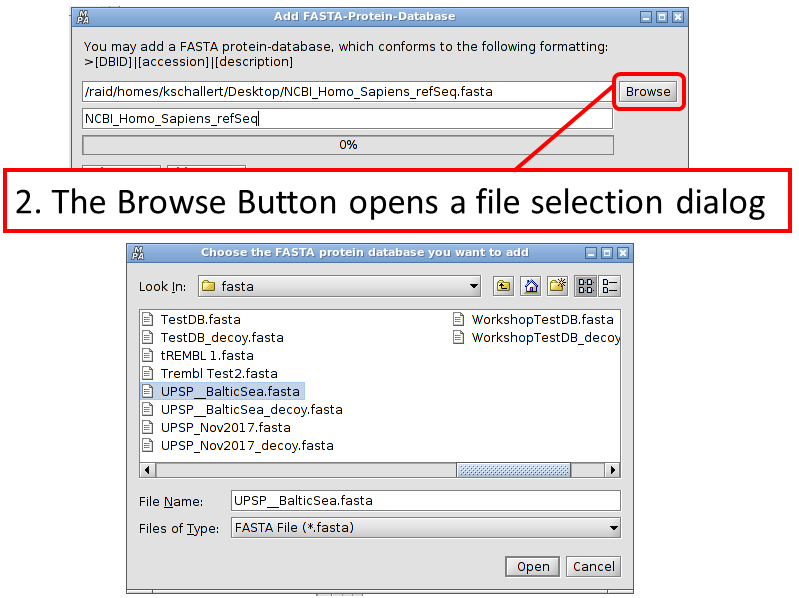


1. Select a name for the new protein database
2. Click **OK** to start the upload, waiting time is approximately 20m – 24h highly dependent on the size of the FASTA file


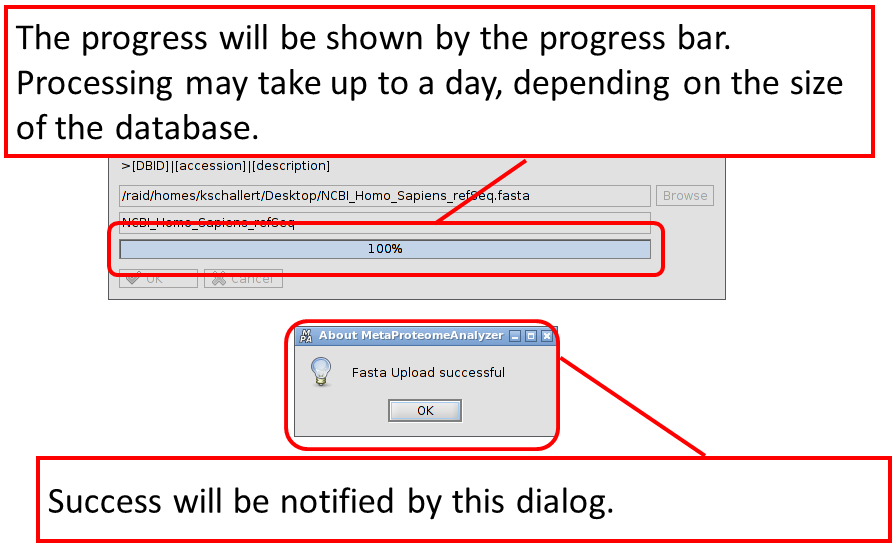

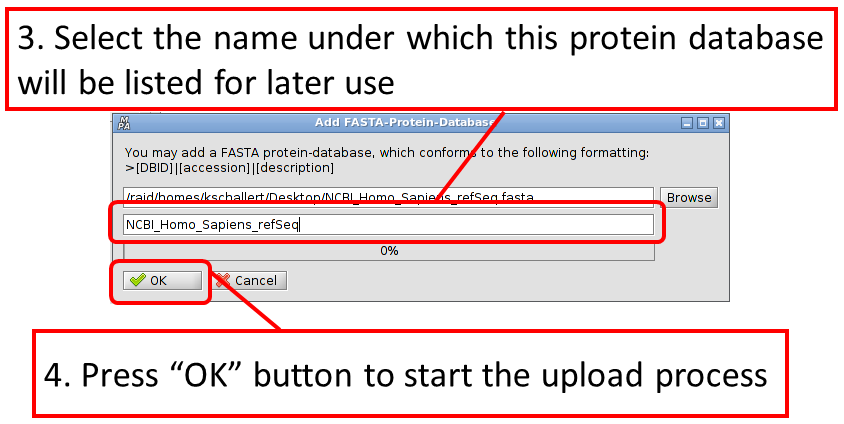


1. After processing is finished, the database will be available in the **dropdown menu** under **Search Settings** for protein database searches


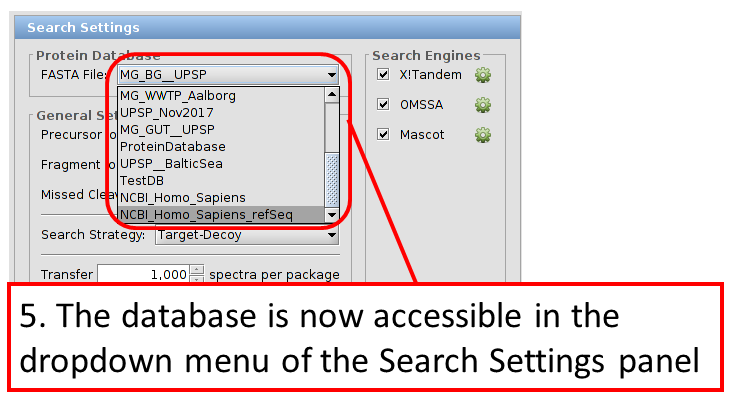


| **FASTA file header structure** | **Comment** |
| --- | --- |
| >sp\|UNIPROT-ACCESSION\|description | UniProtKB/SwissProt entry, metadata will be queried from UniProt |
| >tr\|UNIPROT-ACCESSION\|description | UniProtKB/Trembl entry, metadata will be queried from UniProt |
| >gi\|NCBI-ACCESSION\|description | Old NCBI gene bank entry |
| >ref\|NCBI-ACCESSION\|description | NCBI reference sequence |
| >generic\|ACCESSION\|description | Formatting for metagenomes, variant one, the accession must be unique |
| >mg\| ACCESSION\|description | Formatting for metagenomes, variant two, the accession must be unique |
| >description | Default case for FASTA, not recommended, may cause issues when proteins are checked for redundancy |

## Select Project and Experiment

To properly organize your data, the MPA offers a system where you can arrange your data into *Projects* which in turn consist of *Experiments.* An Experiment is intended as the smallest individual unit corresponding to a LC-MS/MS run or a biological sample. Projects are intended for organizing these experiments. Projects and experiments can also have *Properties,* which can be used to comment on a given item or store experimental or data processing information. Note, that you can load any number of data (files) into a single experiment, which will from that point be considered a single experiment. This is useful when combining Mascot results and search results from the MPA (X!Tandem/OMSSA).

Step-by-step guide: Create or modify a project

1. In the **Project panel** press the **Add project** button.
2. The project modification dialog will appear. Enter a name for the Project into the **New Project Name** text field. The dialog is also used, when projects are modified
3. Press the **Save** button to create the project, it will now appear in the list of projects.
4. The **experiment list** will list all experiment associated with this project, a new project will not contain any experiments yet.


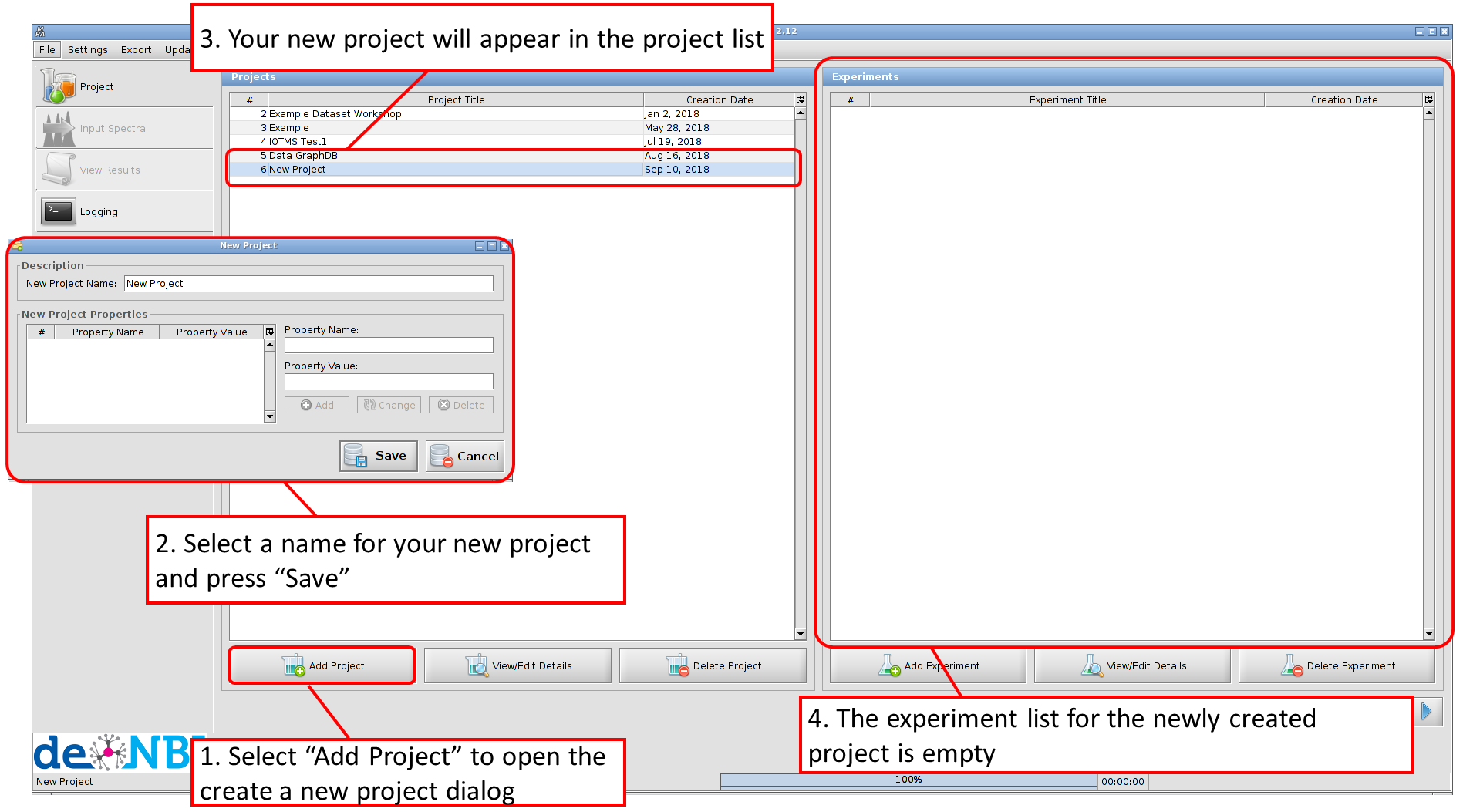


Step-by-step guide: Create or modify an experiment

1. In the **Project panel,** select the project you want to add an experiment to from the list of projects. Press the **Add experiment** button.
2. The experiment modification dialog will appear. Enter a name for the Project into the **New Experiment Name** text field. The dialog is also used, when experiments are modified
3. Press the **Save** button to create the experiment, it will now appear in the list of experiments for the currently selected project.
4. The **experiment list** will list all experiment associated with this project, a new project will not contain any experiments yet.


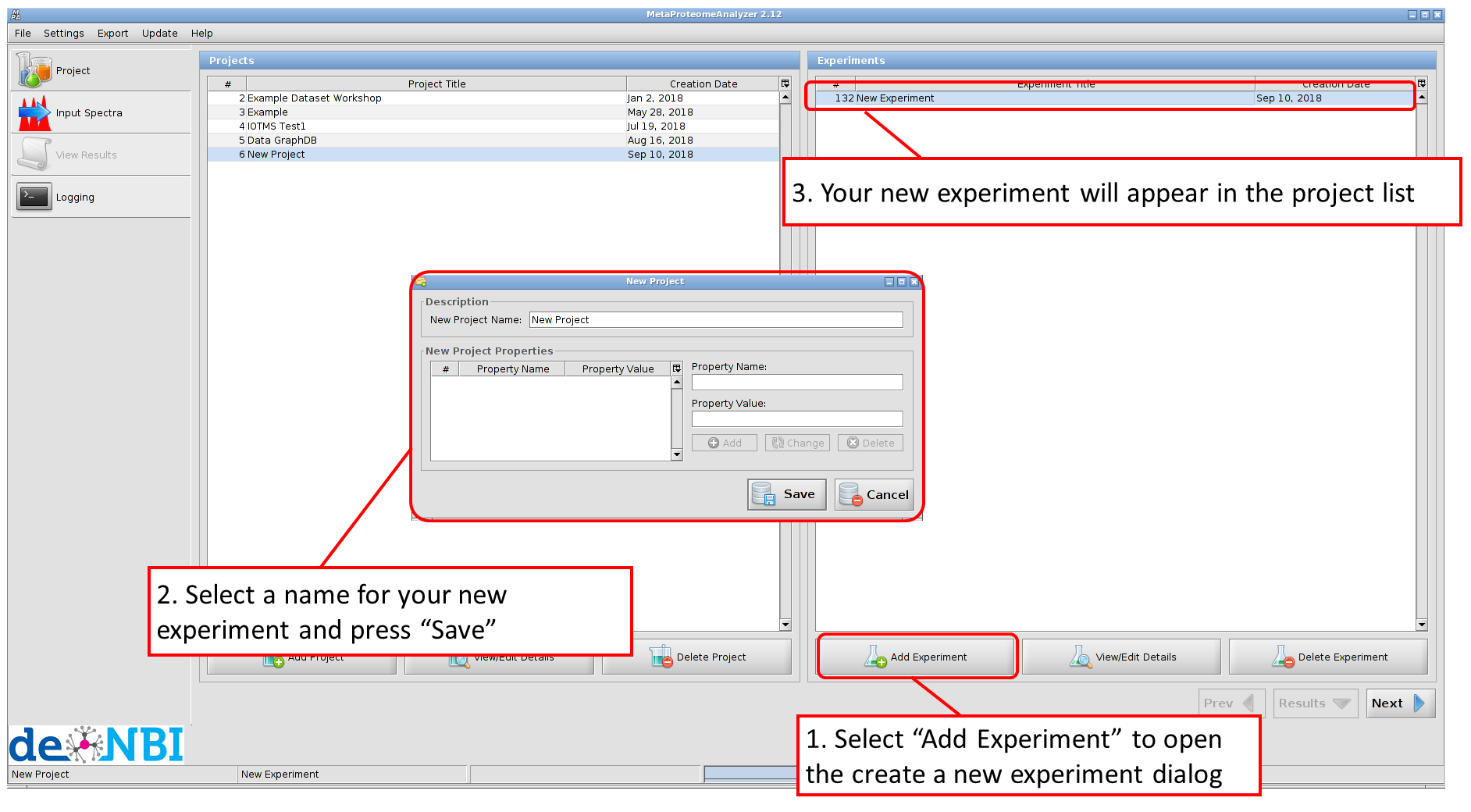


1. The selected project and selected **experiment** will appear in the **status panel** bottom left corner, which is also visible in the other panels.


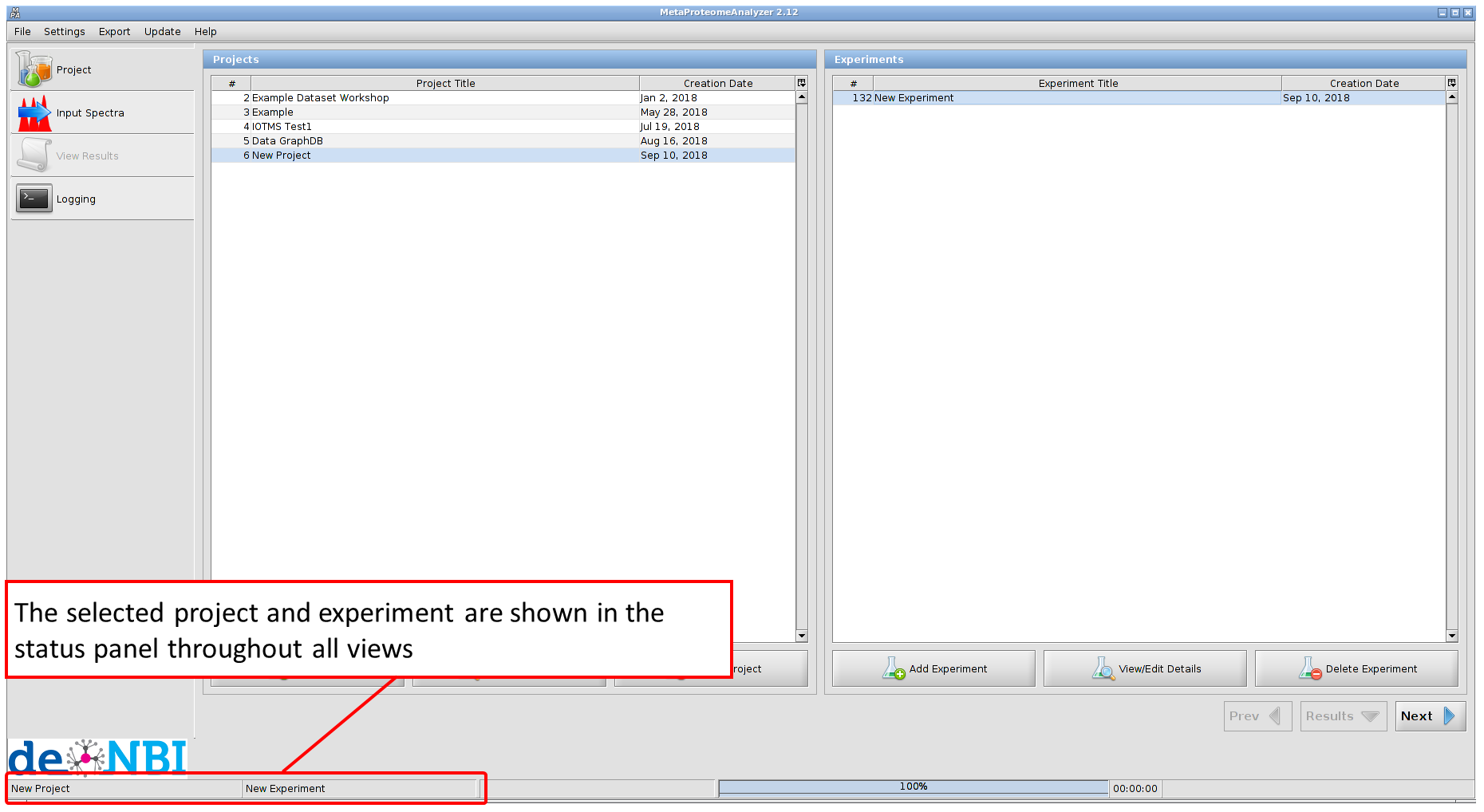


Step-by-step guide: Navigation buttons

1. There are two ways to navigate between the different steps of the workflow, the panel selection organized in four tabs **Project**, **Input Spectra**, **View Results** and **Logging** at the top left and the navigation buttons **Previous** (Prev), **Results**, and **Next** at the bottom right.
2. If a project and an experiment are selected, you can proceed to the **Input Spectra** panel to start protein database searches or load Mascot results.
3. If a project and an experiment are selected and the experiment contains search results, you can also proceed to the **View Results** panel directly, skipping the Input spectra panel.


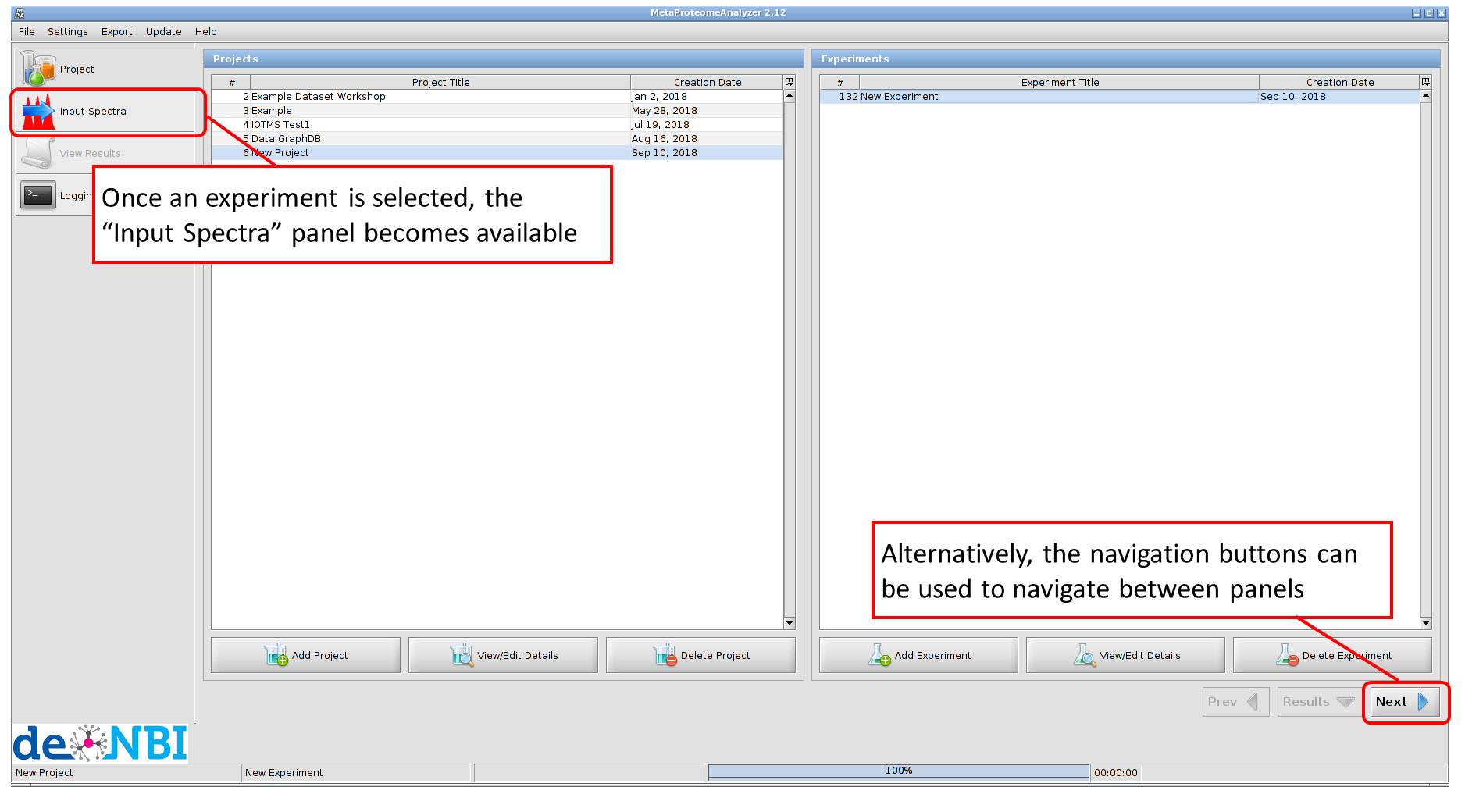


## Input Spectra Panel, protein database searches

The MPA offers support for three protein database search engines: X!Tandem, OMSSA and Mascot. For X!Tandem and OMSSA, searches are fully integrated into the MPA, while for Mascot search results can be loaded, but the searches themselves have to be done separately. Combining two or all three of these search engines in an ensemble approach yields more identifications overall, since variations between these algorithms can produce significantly different results.

The Input Spectra panel offers two methods to load data: 1. Whole-File Input via the “Search Files” button and 2. Selective File Input via the “File Input” panel. The step-by-step guide will deal with the more common whole file input method.

Step-by-step guide: Create or modify a project

1. This figure shows how the Input Spectra Panel is organized:
   1. **Search Settings Panel:** used to modify search parameters and start searches.
   2. **File Input Panel:** Used to load spectrum files or spectra from other experiments and allowing the possibility to select individual spectra or filter them.
   3. **Spectrum Viewer Panel:** Shows spectra that are selected in the File Input Panel


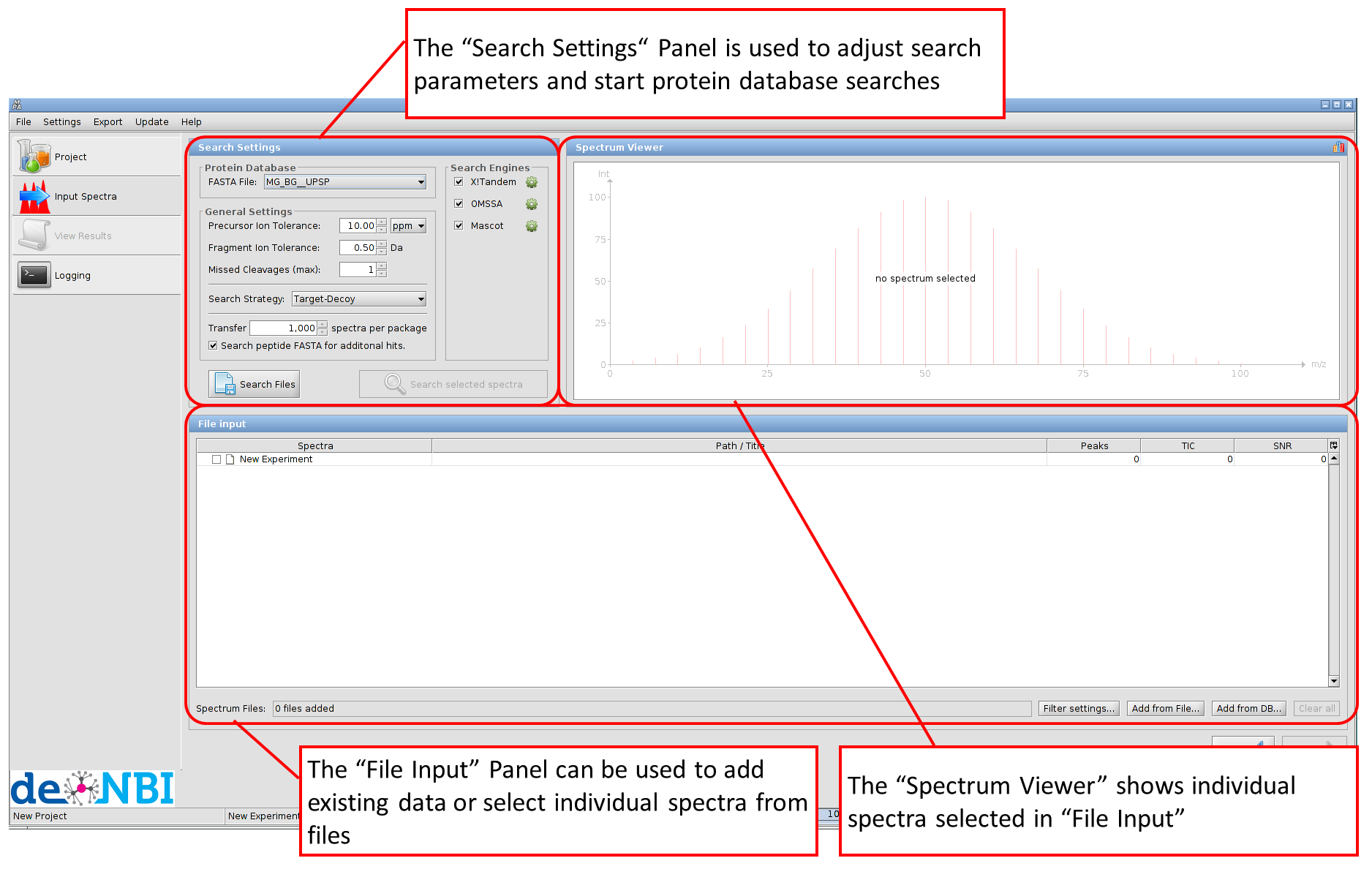


1. **Select protein sequence database:** the most important parameter for protein database searches, choose a protein database you have uploaded using the dropdown menu., this will not apply to loading Mascot results


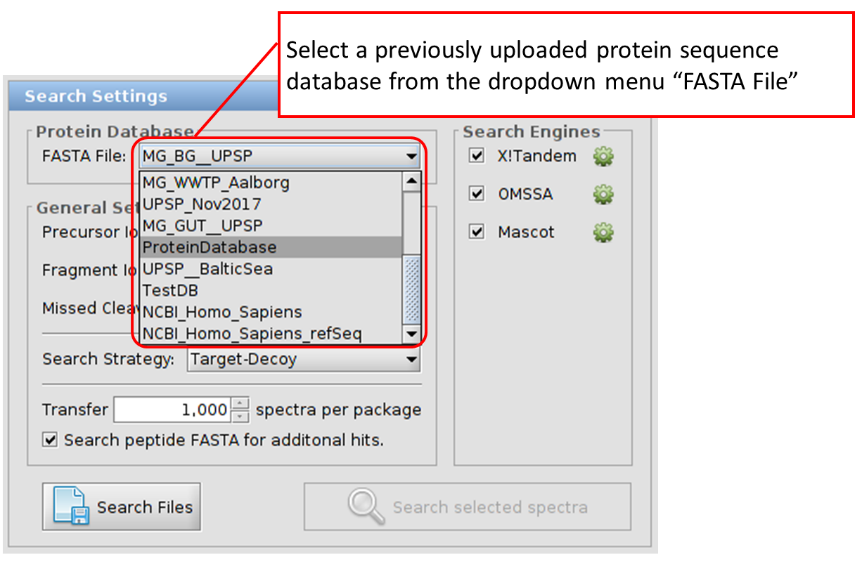


1. **Mass Tolerances:** The most important search parameters are found under “General Settings”: the Precursor Ion Tolerance, the Fragment Ion Tolerance and the number of missed cleavages permissible. Select parameters here that align with the accuracy of your mass spectrometer.


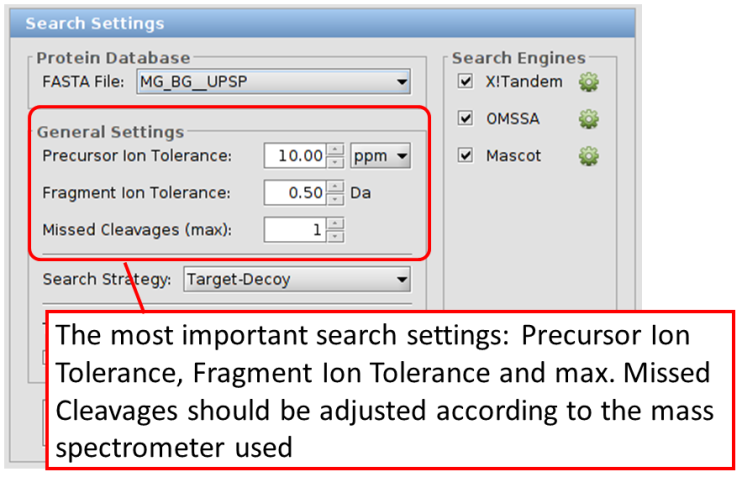


1. **Search engines:** Select which search engines you want to use using the checkboxes. X!Tandem and OMSSA require MGF peak list files and will use the parameter set specified. Mascot results must be provided as DAT Mascot result file. If the required files are not loaded, the search engine will be ignored. The Gear Icons next to the search engine name opens up advanced settings for the corresponding search engine.


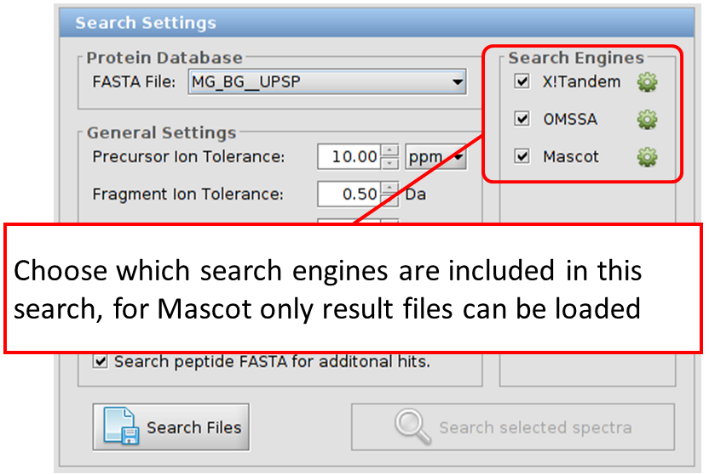


1. Use the **Search Files** button to open the **Search File dialog**


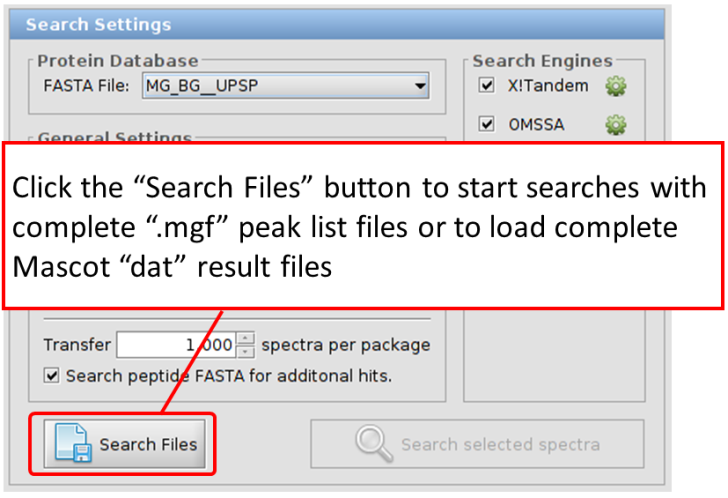


1. **Browse Button:** This button will open a dialog, which will allow you to select MGF and DAT files from the file system.
2. You can choose between **Single Experiment** or **New Experiment for each selected file,** which will determine in which experiment the search results will be stored. The default value, **Single experiment**, will store all data in the currently selected experiment – the naturally expected behavior. The **New Experiment for each selected file** option will ignore the current experiment and will store all results in new experiments corresponding to the name of the selected file. This option is intended for large batches of files that will run for several hours or days.


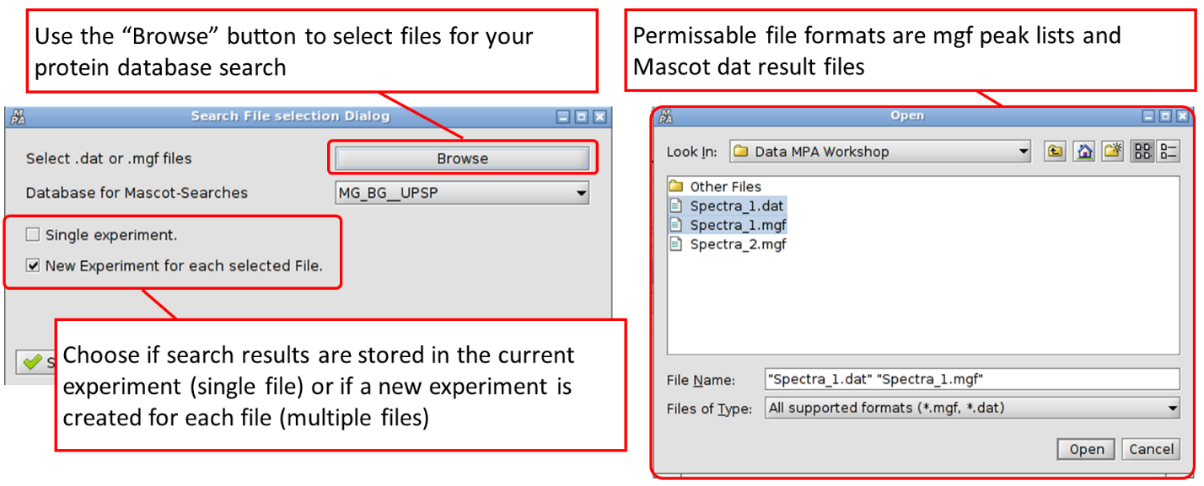


1. In the case that Mascot result files (DAT files) are selected, the exact database that was used to perform these searches needs to be specified in order to connect the proteins that were identified to their metadata. This dropdown menu will contain the same databases as the dropdown menu found in the Search Settings panel.


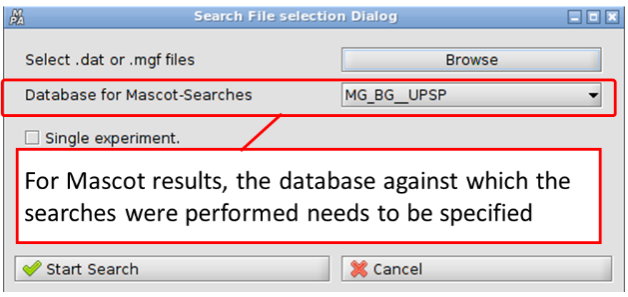


1. Finally, use the **Start Search Button** to actually start the data loading process and the protein database searches, using the currently selected parameters.


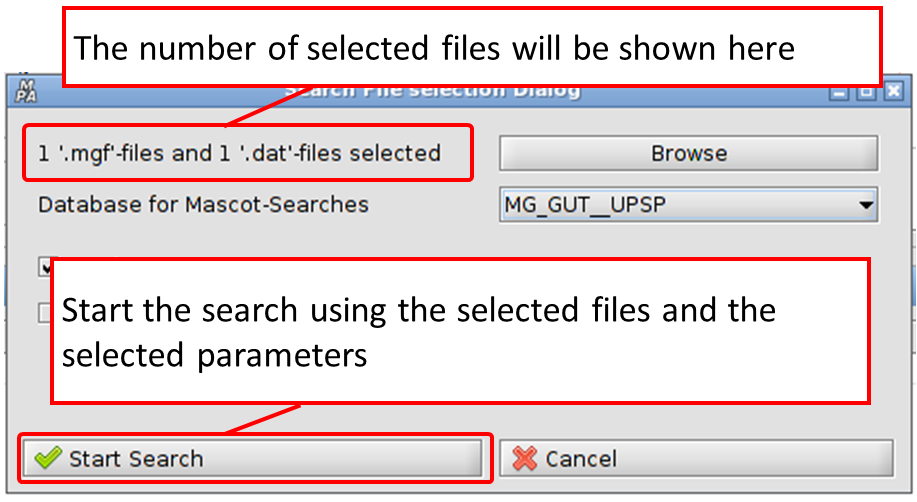


1. The following figure shows where the progress of protein database searches can be monitored using the **Status Panel**. The approximate duration for protein database searches using X!Tandem and OMSSA can be assumed to take 1-2h for every 1 GB of peak list files (MGF). This means choosing a folder with 100 GB of peak list files will take several (4-8) days, which should always be considered when starting searches. Loading Mascot results will progress significantly faster, taking 2-10 min for 1 GB of Mascot result files.


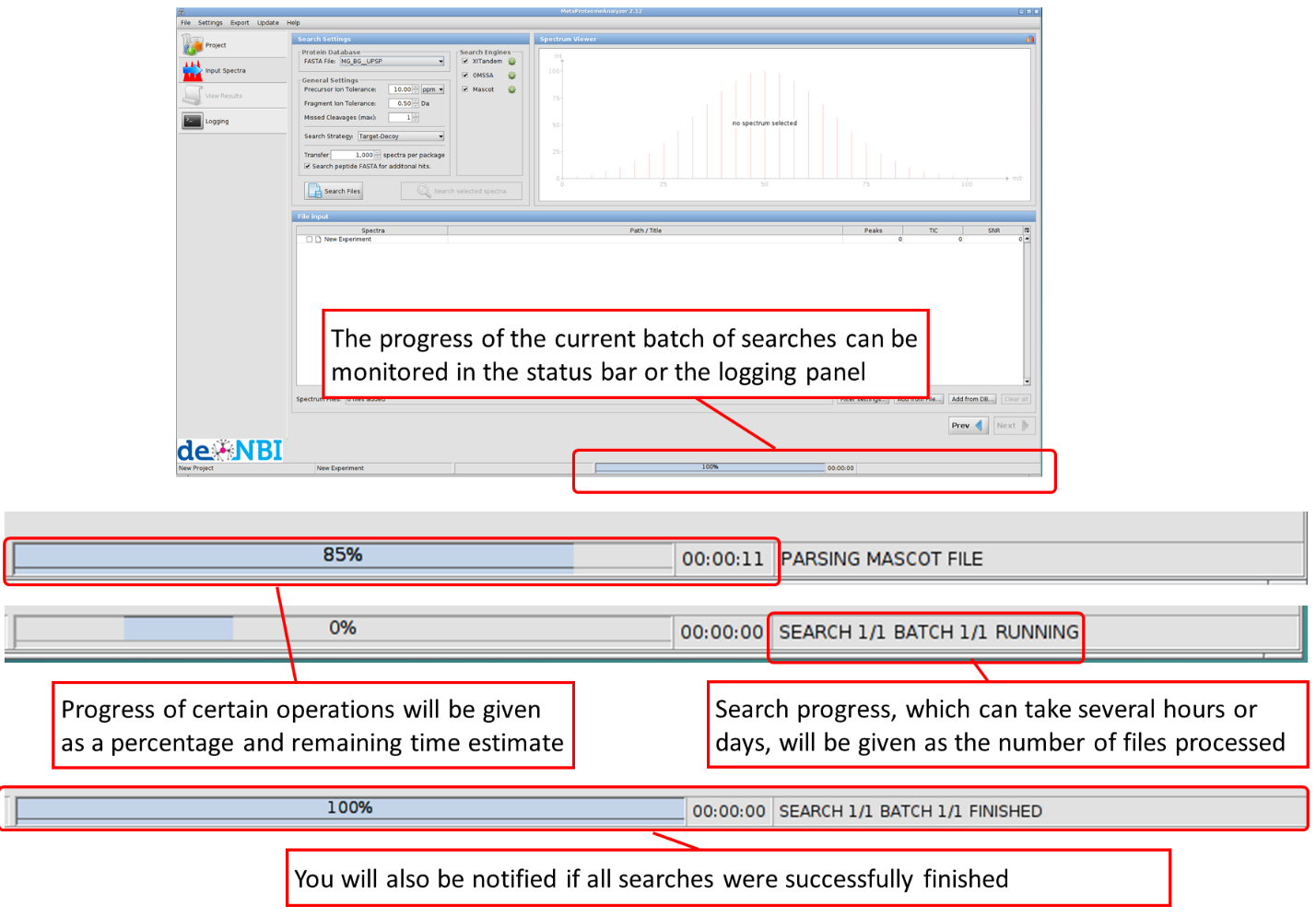


## Fetch Results, Overview and Process Results

The View Results panel is divided into four more tabs: The **Overview** tab, the **Database Search Results** tab, the **Graph Database Results** tab and the **Compare Results** tab. The overview tab is where results from previously selected results are loaded (**Fetch Results** button), which involves processing steps that remove redundancy and preparing the data to be used in all result views and for exports. The **Process Results** button will open a dialog for further processing by setting a desired FDR and metaprotein strategy. If you load results with the Fetch Results button, no metaproteins will be created and a default FDR value will be applied. Therefore, using Process results is intended for refinement, where you can repeat the processing and change parameters until FDR and metaprotein strategy fit to your requirements.

Step-by-step guide: Load and process results

1. If an experiment that contains search results is selected in the Project panel, you can navigate to the View Results panel via the navigation buttons.


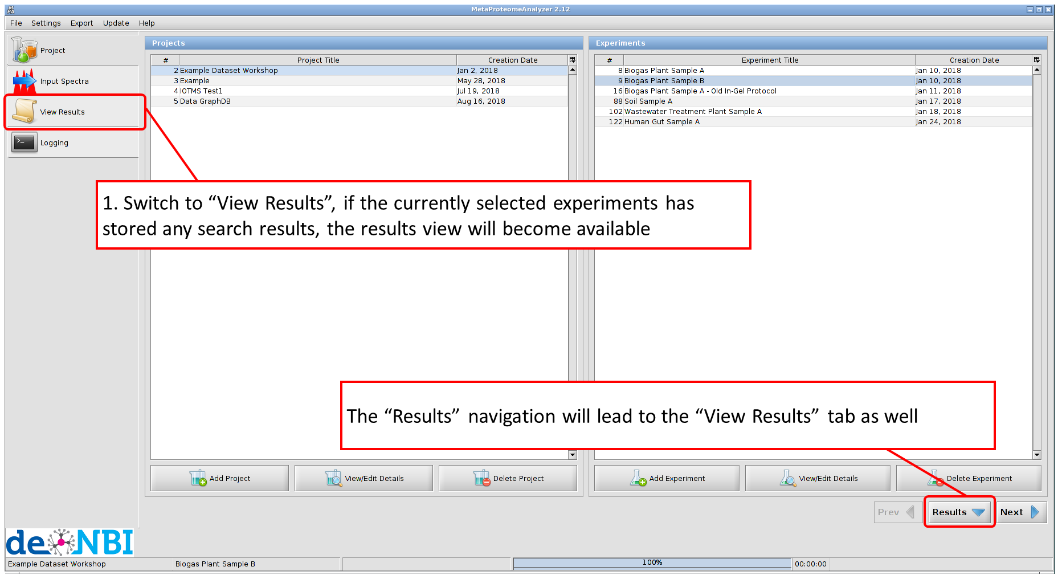


1. The View Results panel will start off in the Overview Tab. Click **Fetch Results** to load the results of the current experiment. You may also combine results from multiple experiments (**Fetch Multi-Results**). Loading results takes 1-10 minutes for typical datasets (1-2 GB), but may take up to several hours for very large datasets.


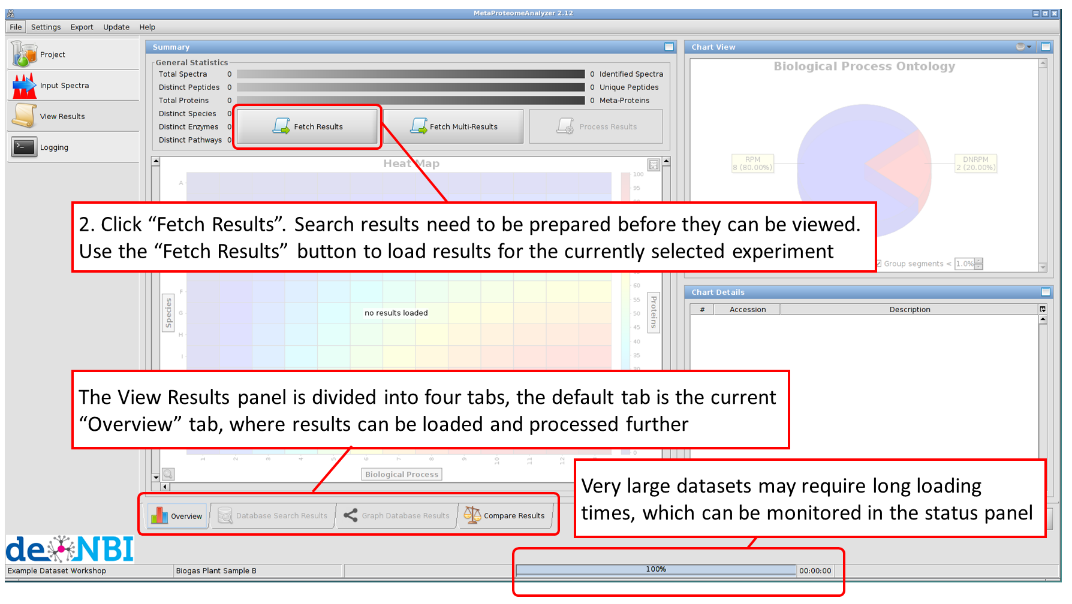


1. Once the results are loaded the **Process Results** button will be available, press it to open the Results processing dialog.


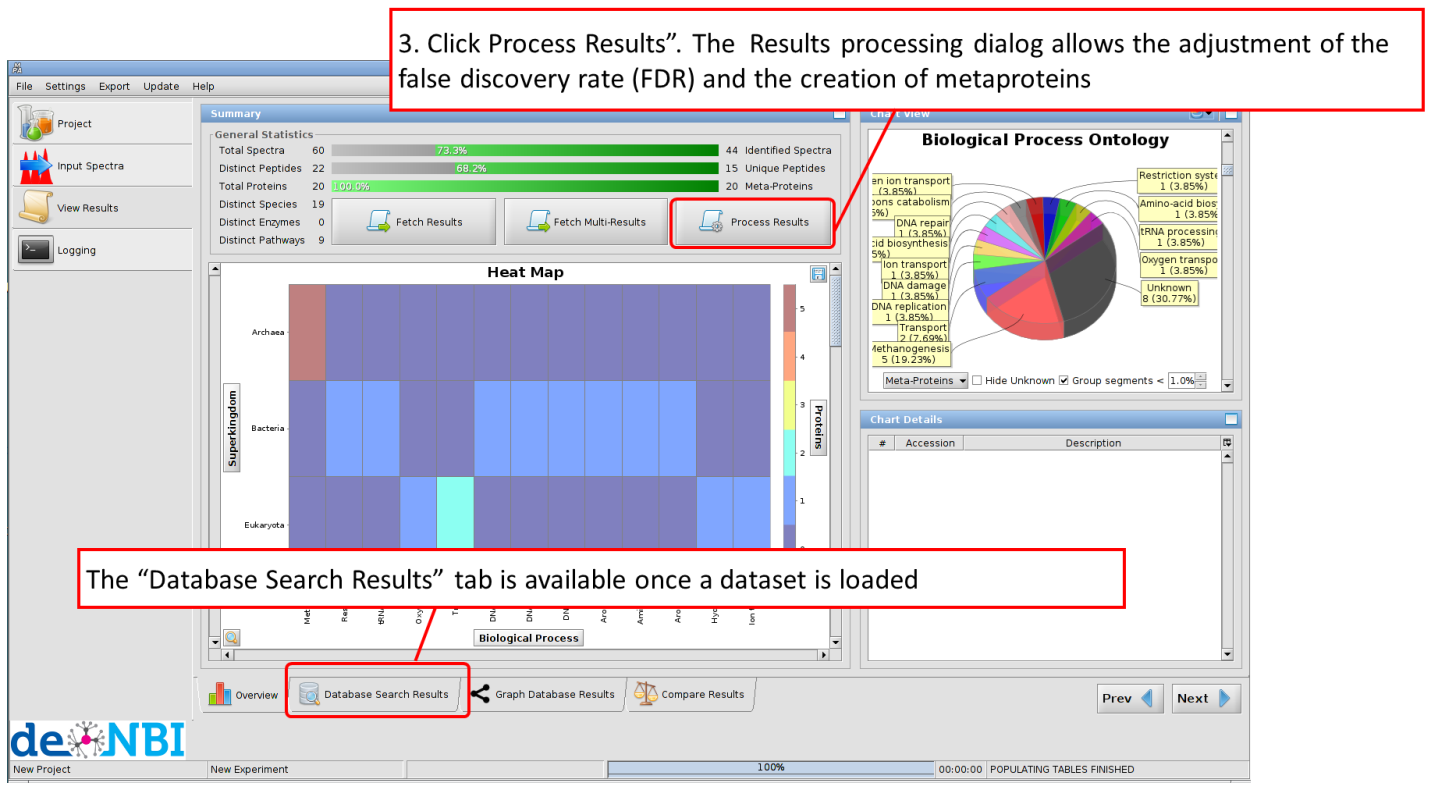


1. Change the false discovery rate (FDR) to suit your requirements, you can increase it again when you repeat the process results step, but PSMs above the default cut off will not be shown.
2. Configure the metaprotein strategy you want to employ, see the section on metaprotein generation for more details.
3. Start the processing by pressing the **OK** button. Depending on the size of the dataset, processing may take a few seconds up to several hours.


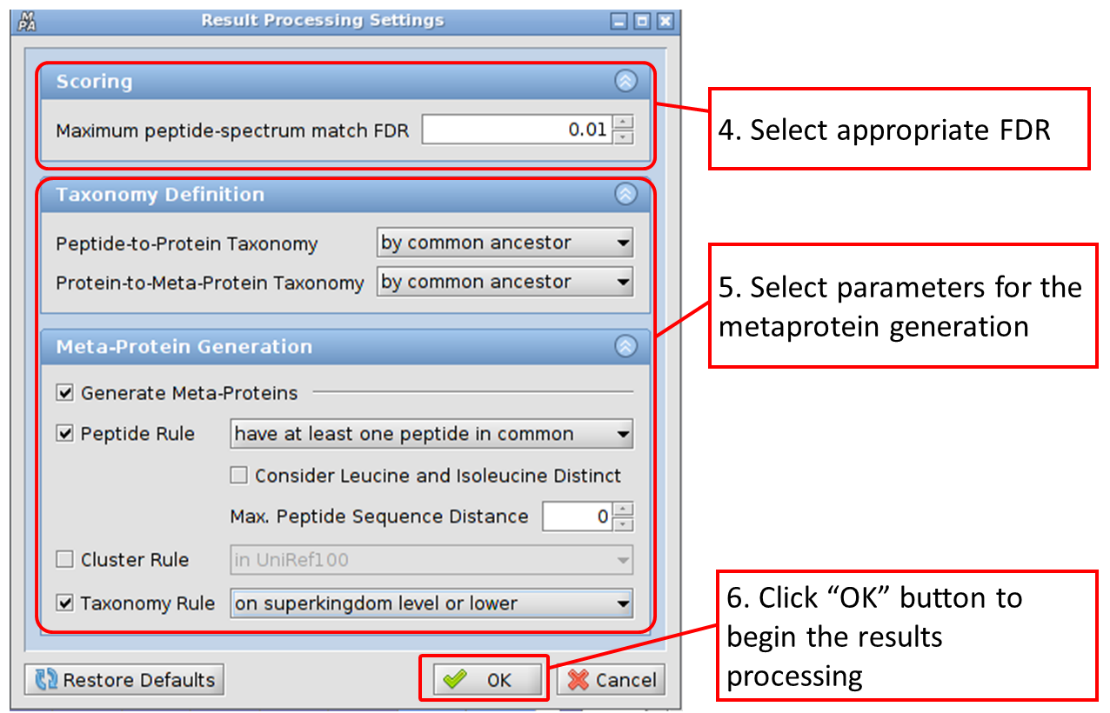


1. The most notable change when you process results is that the number of metaproteins will now be lower than the number of proteins. The **Summary** panel gives you general statistics about spectra, peptides and proteins identified. The Overview tab will also show you the Heat Map and the Pie/Bar charts. To switch to the table view for detailed results, go to the Database Search Results tab at the bottom.


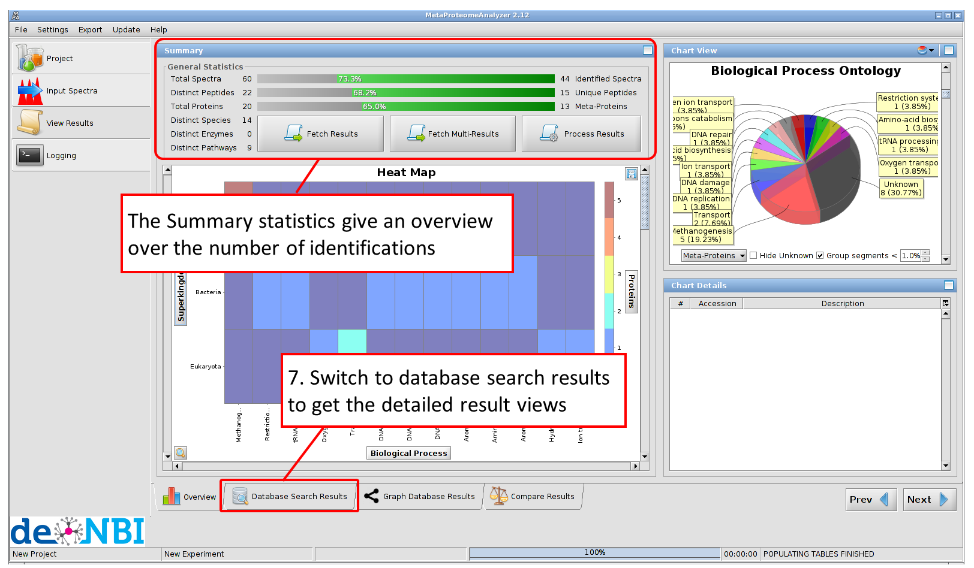


## Database search results view

The database search results view will show you all the detailed information about your data including different protein table views, peptides, PSMs, individual spectra and charts for taxonomic and functional analysis. Most notably, the different protein table views will arrange proteins. In the following all the important elements of the Database Search Result panel will be shown. The following table lists all the available protein table views. A major feature of these table is, that selection changes made in one table will apply to all other tables. This means, for example, that you can select certain taxonomies in the Taxonomy View, then switch to the Pathway View, which will now only show pathways for the selected taxonomies.

| **Protein table view** | **Description** |
| --- | --- |
| Basic View | Lists all identified proteins non-redundantly |
| Meta-Protein View | Lists metaproteins, where child elements of a metaprotein are the proteins that belong to a metaprotein |
| Ontology View | Shows a tree for UniProt Keyword Ontologies, the leaf nodes of this tree are the proteins that fit into the given keyword category. The tree will contain individual proteins multiple times (e.g. several Keywords or EC numbers). Selecting or Deselecting a protein will apply the change to all instances of this protein. |
| Taxonomy view | Shows a tree for NCBI protein taxonomy, the leaf nodes of this tree are the proteins that belong to a certain taxonomy. |
| Enzyme View | Shows a tree of Enzyme Commission numbers (EC), the leaf nodes of this tree are the proteins that fit into the EC category. The tree will contain individual proteins multiple times (several Keywords). Selecting or deselecting a protein will apply the change to all instances of this protein. |
| KO View | Lists KEGG Orthology numbers (KO) – the KEGG classification system of proteins. Proteins are associated with the KO based on the mapping from UniProt. |
| Pathway View | Lists KEGG pathways and the proteins that belong to specific pathways. Clicking on a specific pathway number (blue link), will open the systems browser and load KEGG pathway with the identified proteins colored in. |

Step-by-step guide: Database Search Results

1. The protein list is central part of the results and is arranged as a sortable list showing the protein accession and several other data associated with a given protein. To switch to other protein table views, press the icon in the upper right corner of the table. There are two levels of selection to the tables: highlighted selection, which will use the selected element for other tables and the checkmark selection, which will/will not consider a given element for counts and exports.


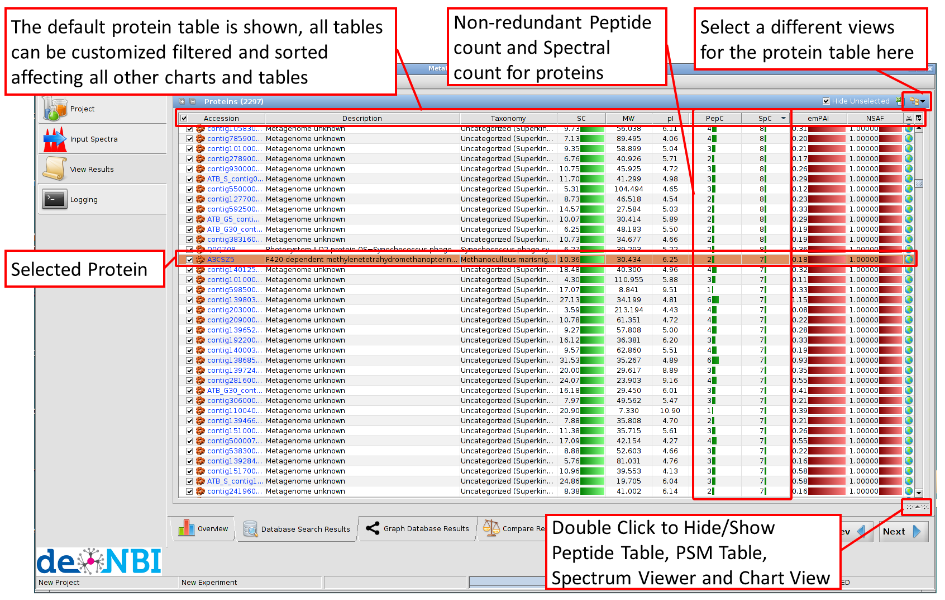


1. If a protein is selected (highlight selection), its peptides will be shown in the Peptides table. Similarly, if a peptide is selected, its PSMs will be shown in the Spectrum Matches table and if a PSM is selected, its spectrum will be shown in the spectrum viewer. These views are intended to be used for assessing the quality of the results, for instance the protein hit shown below is of very high quality, since it contains many peptides, which contain many PSMs from different search engines and the quality of the PSM is high since it identifies most of the peaks in its spectrum.


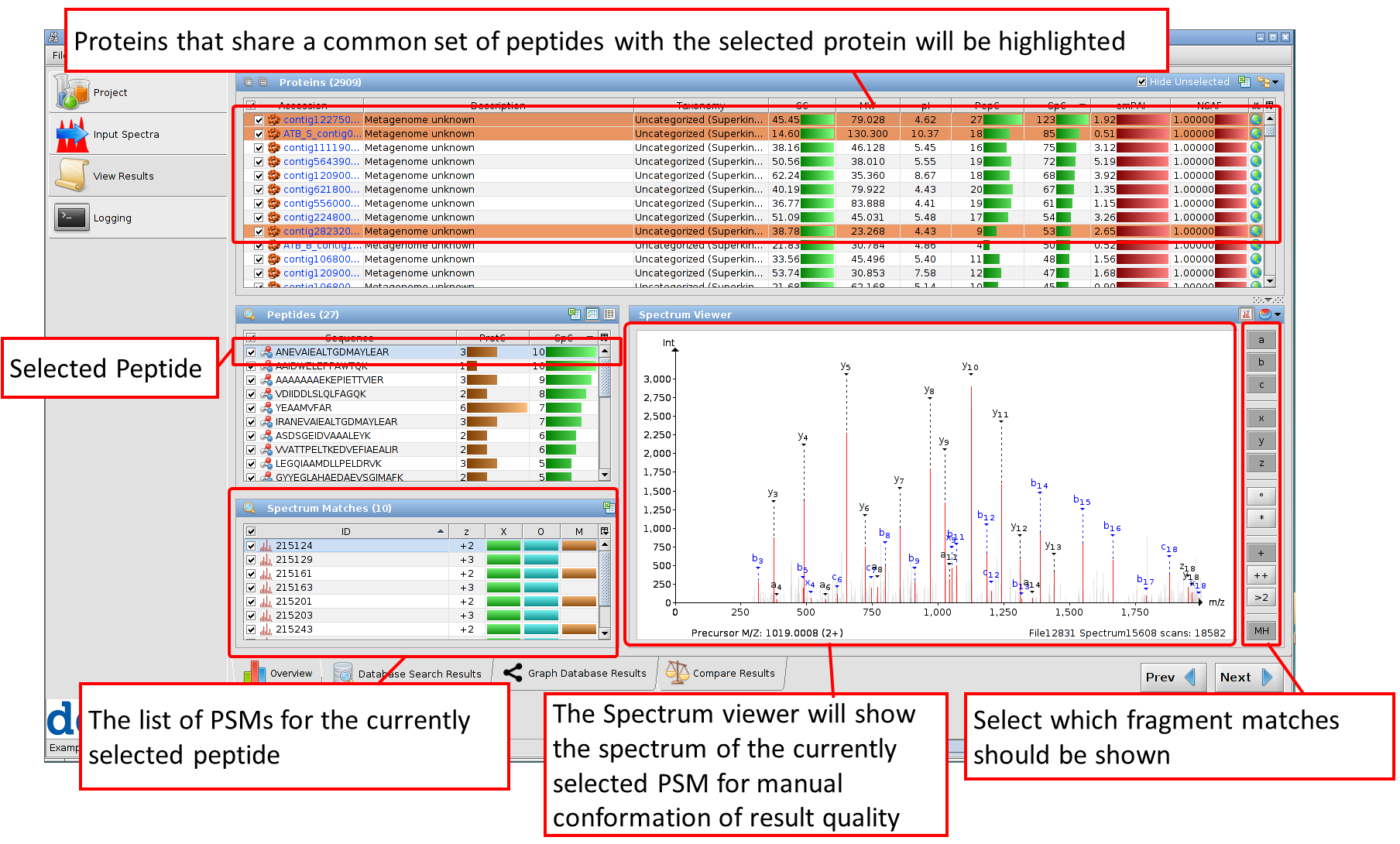


1. Switch to the **Meta-Protein View** using the dropdown menu in the upper right corner. Metaproteins greatly reduce the redundancy introduced by homologous protein sequences. Peptide and Spectrum counts towards metaproteins will be counted non-redundantly. You should use metaproteins instead of proteins for the purpose of presenting protein identifications as charts or tables or for further statistics. The table below lists all protein table views available.


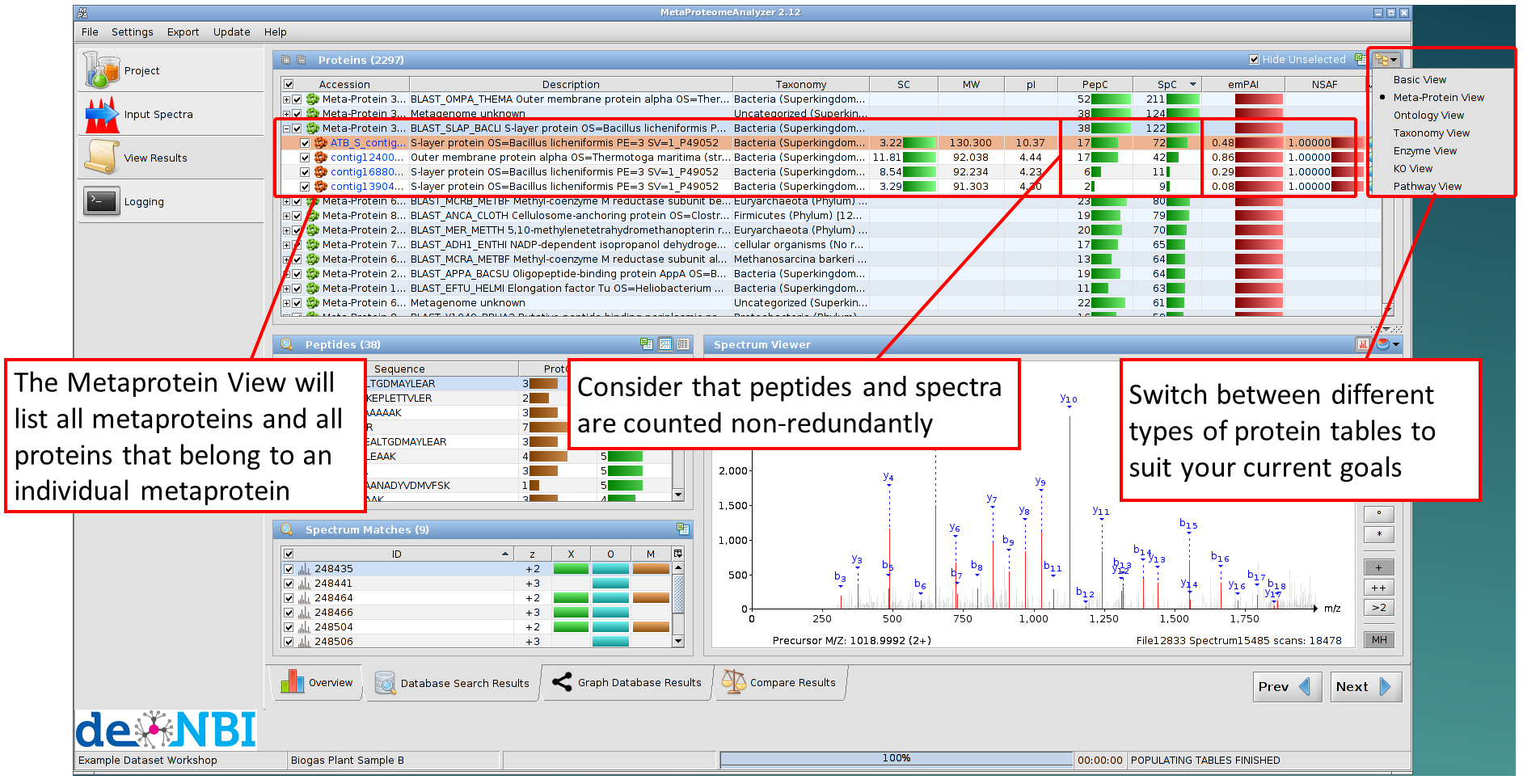


1. The **Taxonomy View** and **Pathway View** are shown in the two following figures. The Taxonomy View will show a taxonomic tree with the proteins inserted at the point of their assigned taxonomy. This view is great to deselect certain taxa if you do not want to consider them in your analysis (checkmark deselect). The Pathway View will list all KEGG pathways that are identified by the protein identifications. Clicking on a pathway number will open the browser and show the pathway with all the proteins identifying it colored in red.


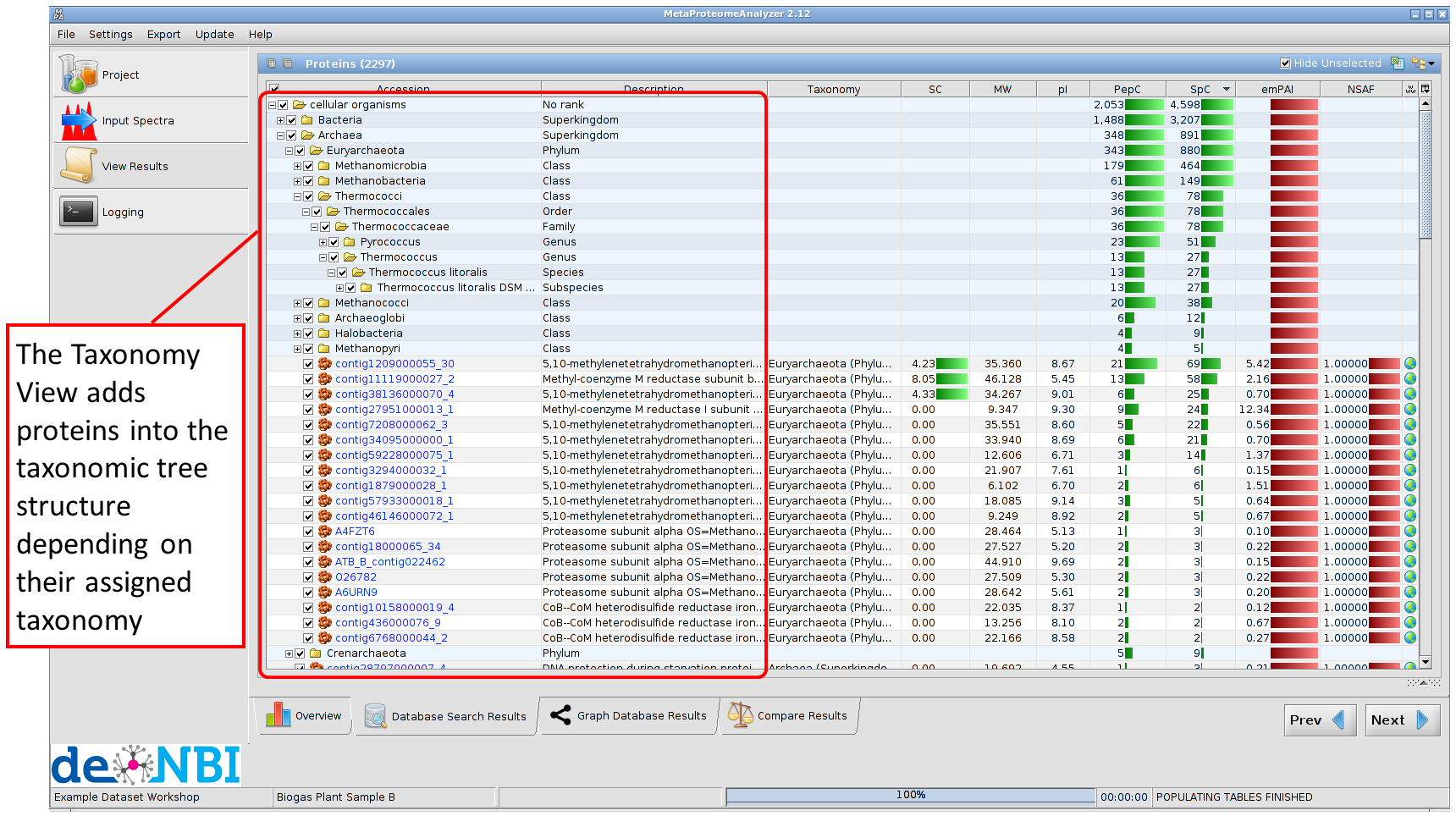


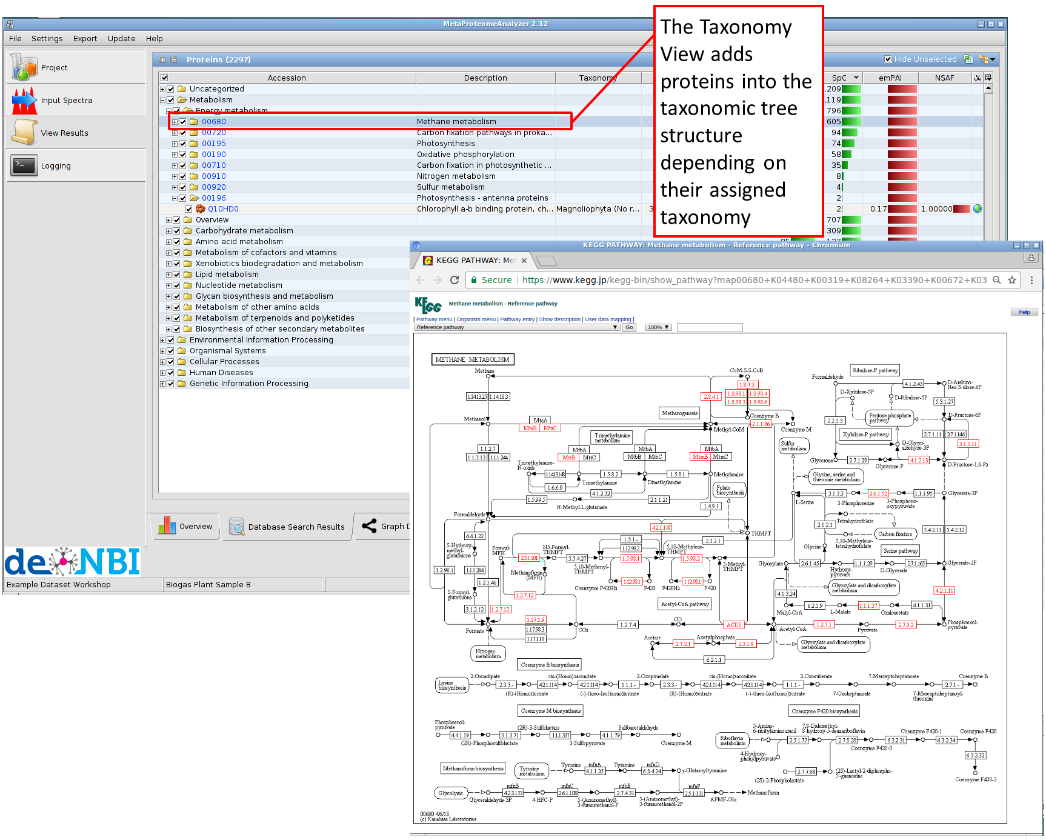


1. The **Detail Charts** view can be reached by clicking on the pie chart symbol above the Spectrum Viewer. Highly customizable charts are available for taxonomies and UniProtKB Keywords ontologies.


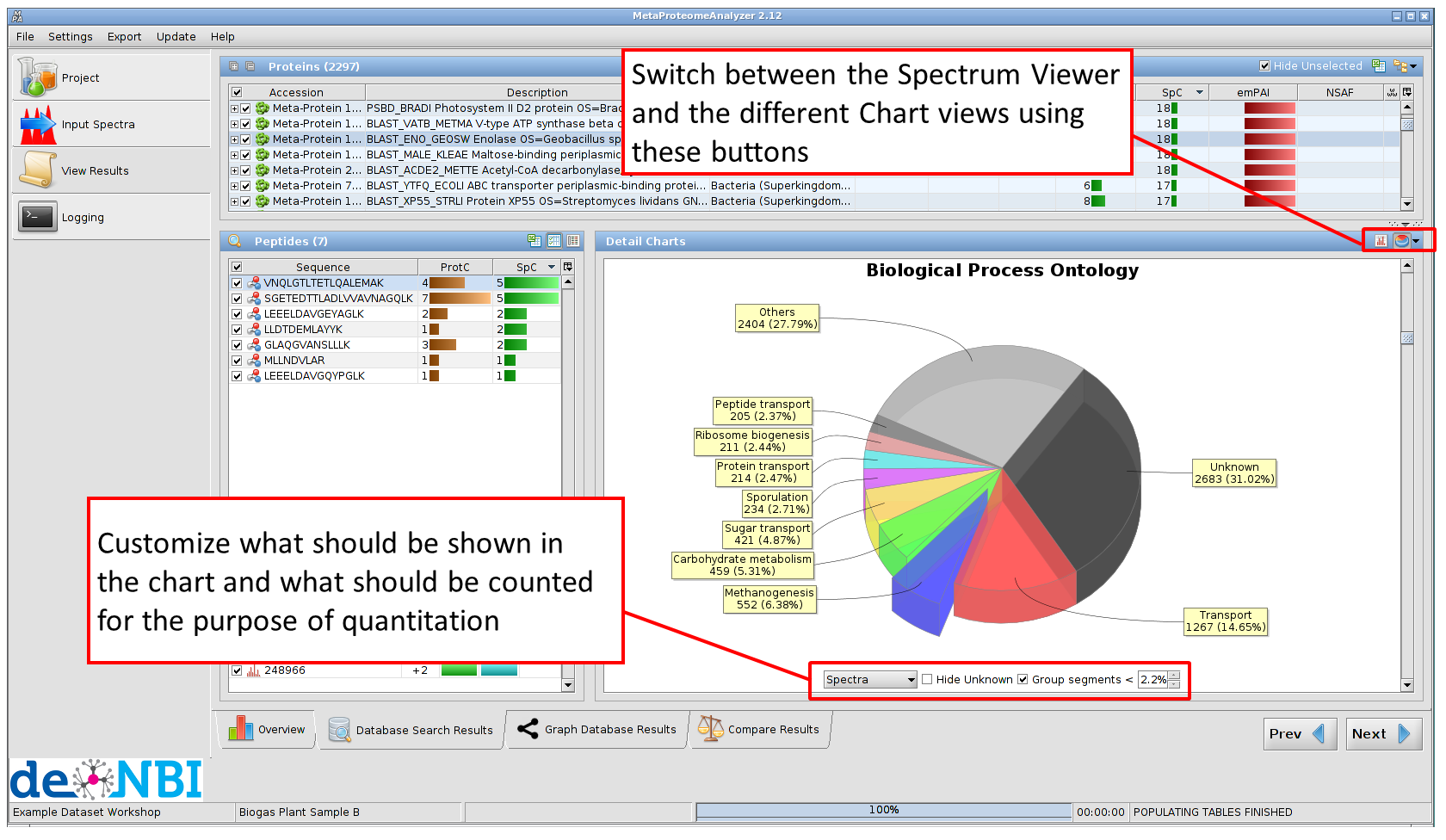

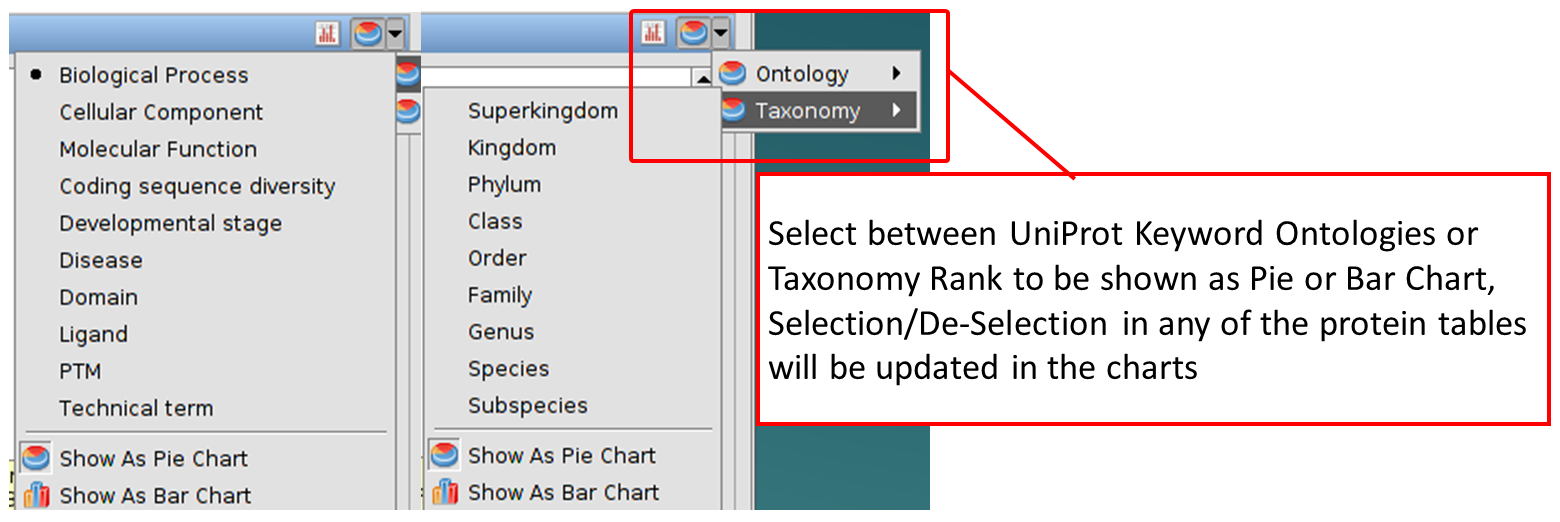


## Annotate unknown proteins via BLAST

Often in metaproteomics, metagenomes are used, that do not contain functional and taxonomic annotation like protein database from UniProtKB. To get some functional and taxonomic information for these protein sequences, a typical strategy is to do a sequence similarity search using BLAST and use the first protein hit in this search to annotate the metagenome protein with metadata. The MPA relies mostly on the well curated UniProt metadata and also integrates the possibility to do an automated BLAST on experimental results or entire databases. A key feature is the automated use of multiple BLAST hits, instead of just one, which takes account of the fact that often multiple equally confident BLAST hits are found. Six strategies can be used to annotate a protein from the BLAST hits as can be seen in the table below.

Step-by-step guide: Annotate protein sequences with UniProt metadata via BLAST

1. In the menu bar under **Update**, the options **BLAST unknown hits** and **Delete Blast Hits** can be found. Choosing BLAST unknown hits will open the BLAST dialog.
2. The default value for the protein sequence database is UniProtKB/SwissProt and should suffice in most cases.
3. Select for which experiment BLAST should be performed by specifying an experiment ID. The experiment ID is shown in the Experiment table in the front column. If you leave this value at “-1”, all experiments in all project will be used and all identified proteins from these experiments will be subjected to a BLAST search. Choosing “Global BLAST” will search all proteins from all protein databases – including proteins that were never identified - and is not recommended since it may take several weeks for typical databases.
4. Choose a BLAST hit combination strategy. The recommended strategies are “Best Identity” and “Best E-value”. For further information, see the table below.
5. Press the **OK** button to start BLAST. Only proteins that are not associated with any UniProt metadata are searched (i.e. metagenome). Proteins that were searched via BLAST previously will also be excluded.


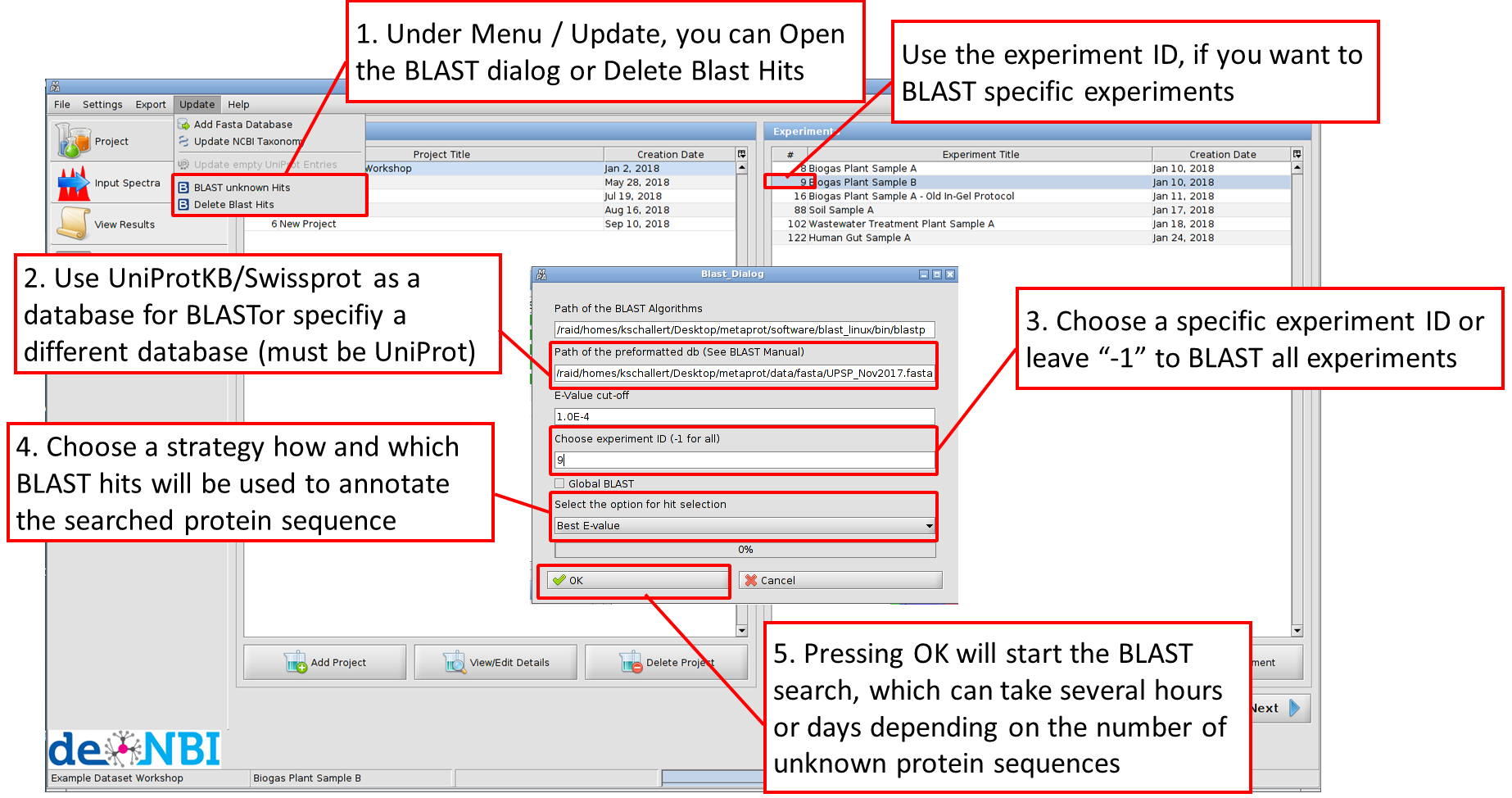


1. The BLAST dialog will report progress for the current experiment and the Status panel will show you which experiment is currently searched. For typical experiments with thousands of unknown proteins, BLAST will take up to one hour per experiment. When multiple experiments (“-1“) are selected, processing may take several days. Once the processing is finished, the BLAST dialog will disappear and the Status panel will report “BLAST FINISHED”.


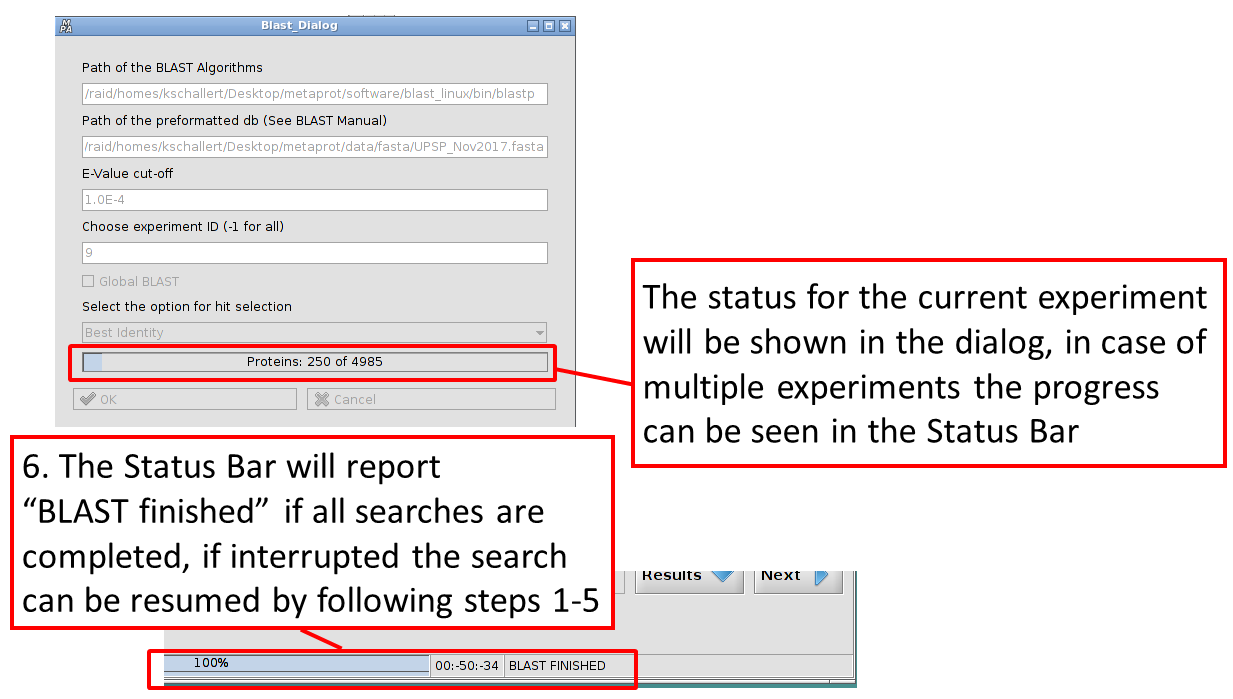


| **Strategy for BLAST hits** | **Description** |
| --- | --- |
| Best E-value | The E-Value is used to rank BLAST hits. From this ranked list, all hits that share the same E-Value as the first hit will be combined to create the annotation for the protein entry. |
| Best Identity | The sequence identity is used to rank BLAST hits. From this ranked list, all hits that share the same identity as the first hit will be combined to create the annotation for the protein entry. |
| Best Bitscore | The Bitscore is used to rank BLAST hits. From this ranked list, all hits that share the same Bitscore as the first hit will be combined to create the annotation for the protein entry. |
| First E-value | The E-Value is used to rank BLAST hits. From this ranked list, only the first entry will be used for annotation. |
| First Identity | The sequence identity is used to rank BLAST hits. From this ranked list, only the first entry will be used for annotation. |
| First Bitscore | The Bitscore is used to rank BLAST hits. From this ranked list, only the first entry will be used for annotation. |

## Export Results

The MPA offers many export functions, which can be used to generate customized figures or apply further statistics. Export functions are available for tables, charts, the compare panel results and the heat map. Furthermore, specialized export functions are accessible through the export menu.

Step-by-step guide: Export functions

1. All the tables of the Database search results panel can be exported as comma separated value file using the **Spreadsheet Icon** in the top right corner of the particular table. In the Export Dialogs, you can specify the columns that you want to export. The tables will be exported “as seen”, meaning hidden elements will be ignored unless they are deliberately shown.


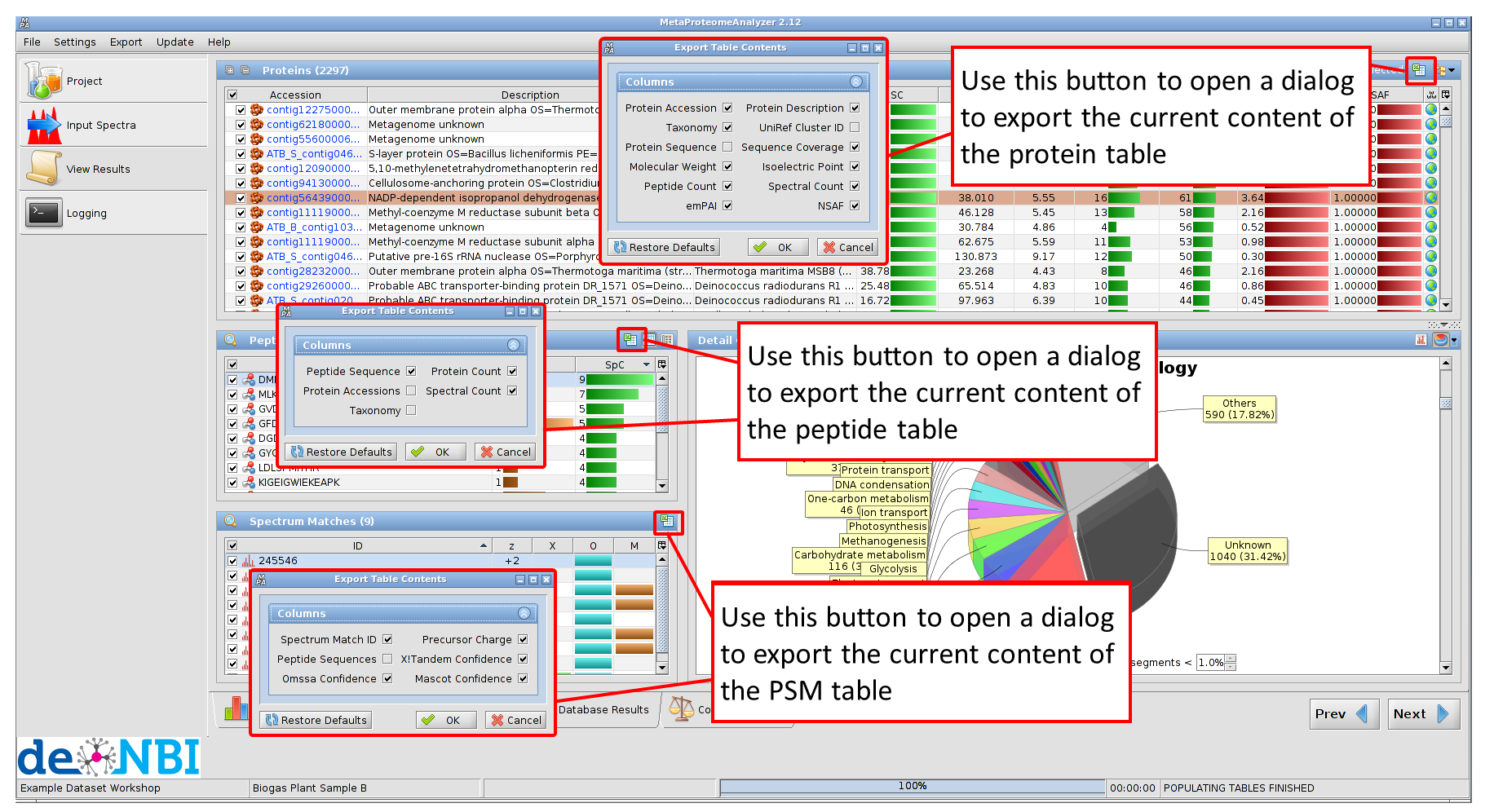


1. To export the Chart View data into a CSV file, right click on the empty chart area and click “Save as CSV…”.


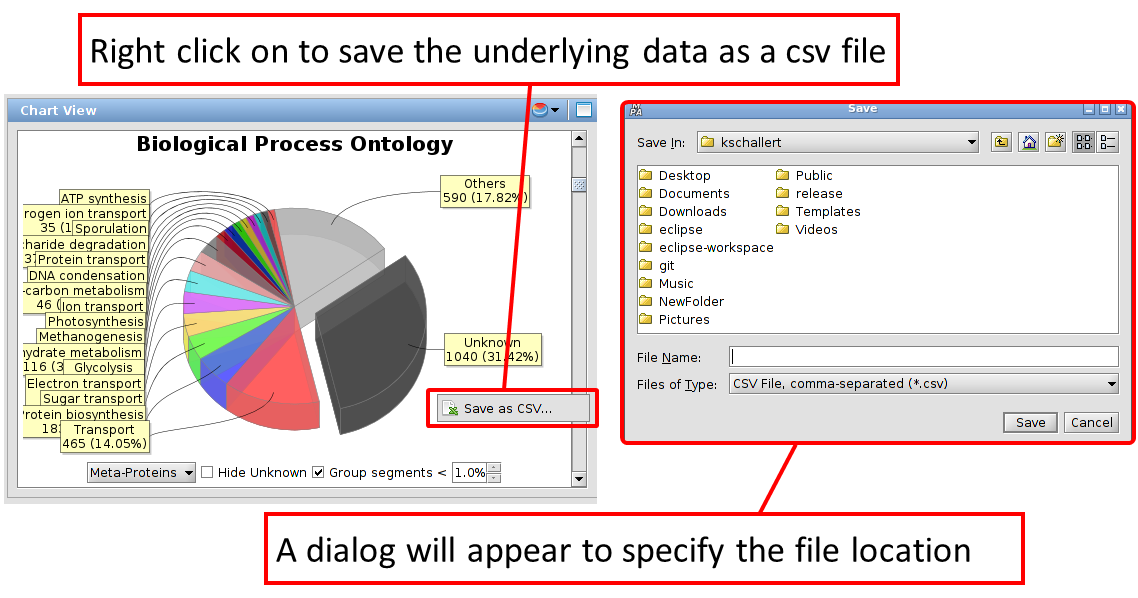


1. To export the complete heat map, click the Disk Icon in the upper right corner. The image will be saved as a PNG file and will include all elements ignoring the current zoom level.


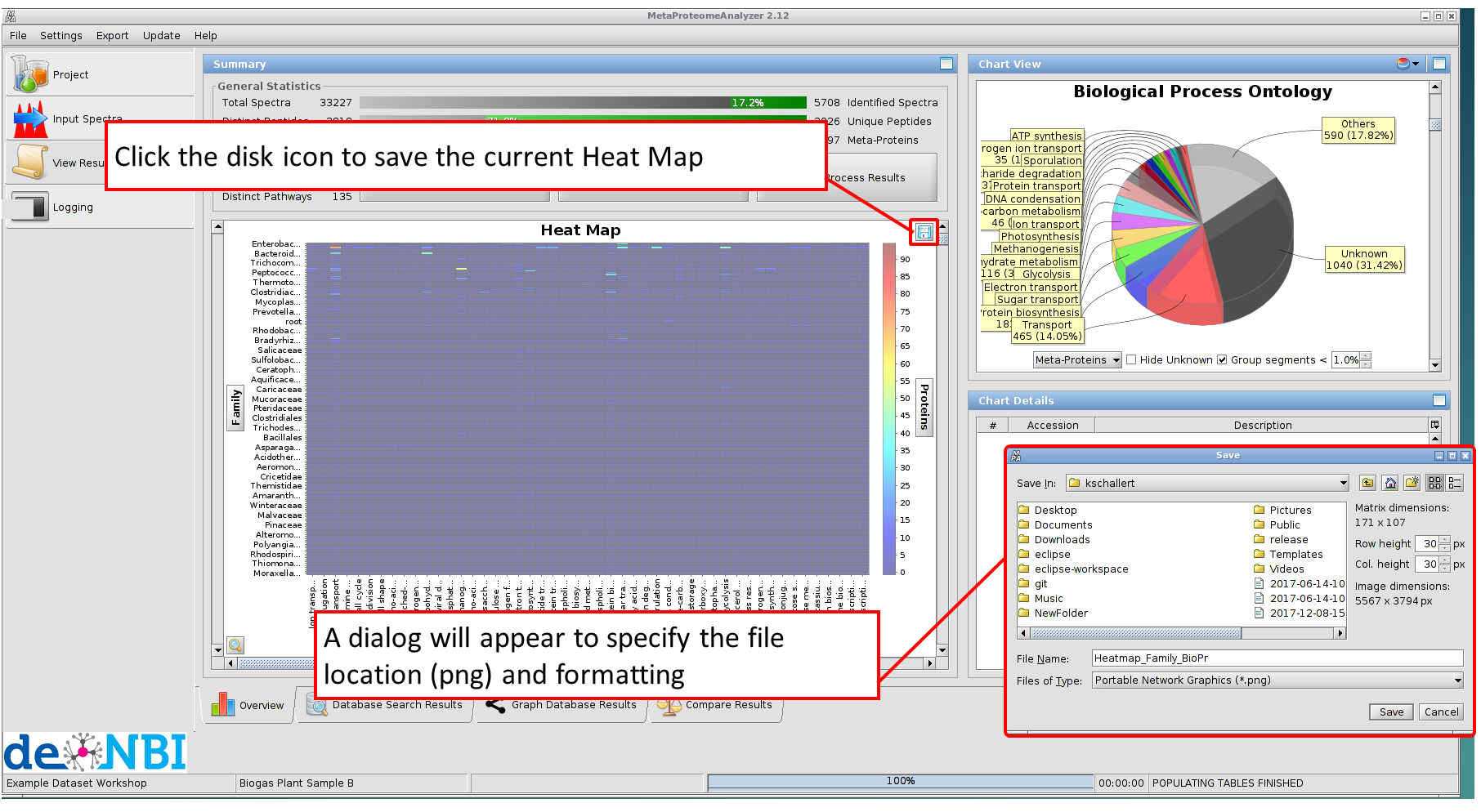


1. In the **Export Menu** you can find the **“CSV file …”**option, which allows you to export many different data from the currently loaded experiment. Of particular interest are the Metaprotein export, the Krona export and the Chord visualization export.


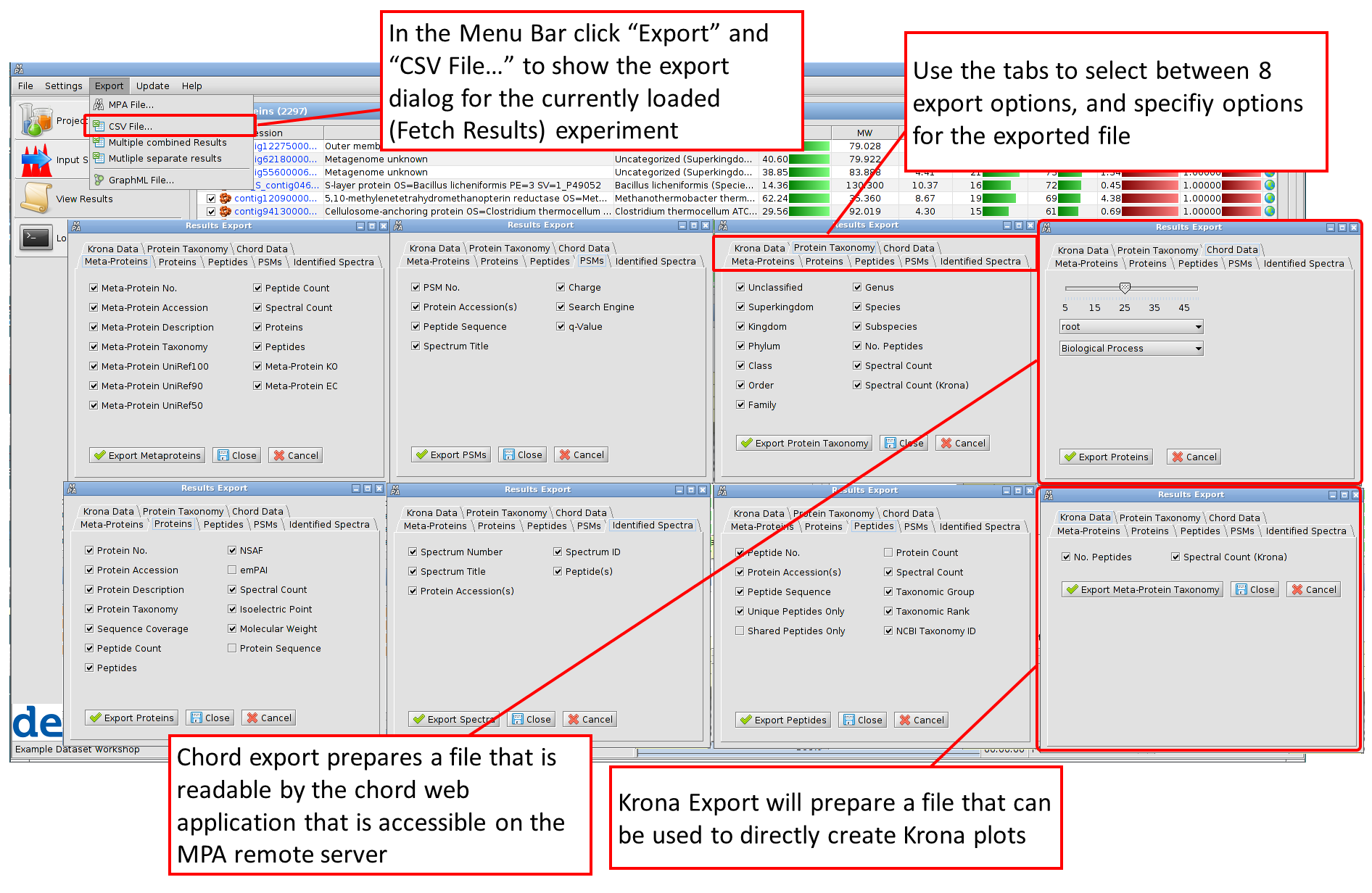


##

## Compare Results

The compare results panel allows the comparison of any number of experiments on the levels of metaproteins, proteins, peptides, taxonomies and ontologies using spectra or peptide counts as comparison value. The main feature of this comparison functionality, is that the comparison categories (i.e. metaproteins) are created using the data of all selected experiments, removing the danger of inconsistencies.

Step-by-step guide: Compare Panel

1. To compare experiments, switch to the Compare Results panel and add experiments by clicking into the experiment list which shows **“Click here to add an experiment”.** In the upper right corner, select the comparison category and quantification count. To adjust settings for metaprotein and FDR, use the **Gear Icon** in the upper right corner of the Compare button. Pressing the **Compare** button will start the comparison, which will take several minutes up to several hours, depending on the size of the data.


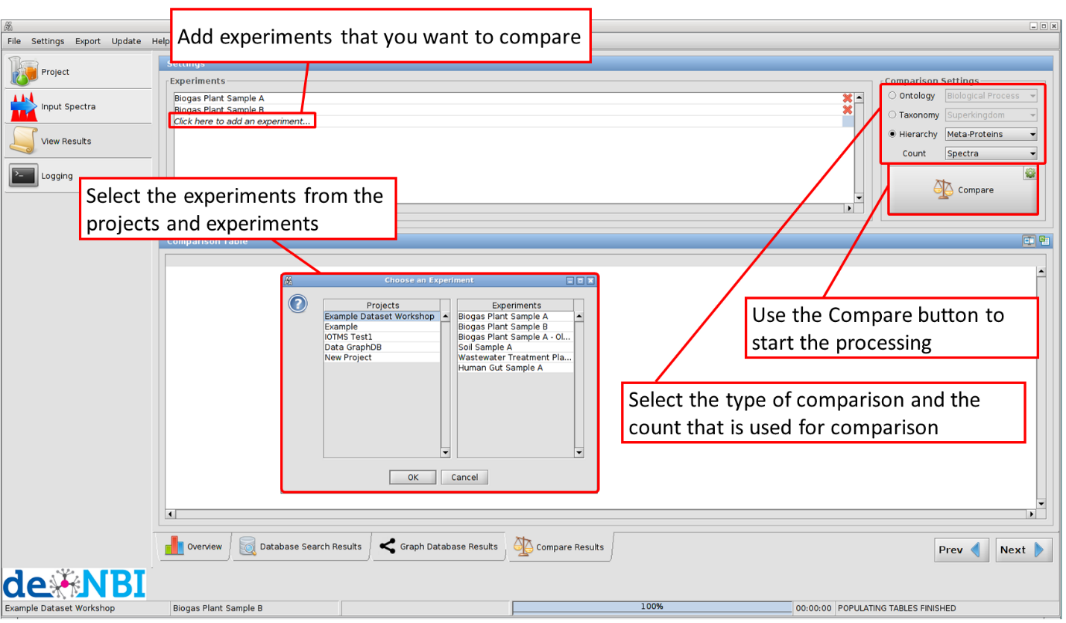


1. Once the comparison is finished, export the created table as CSV file using the **Spreadsheet Icon** in the upper right corner of the comparison table.


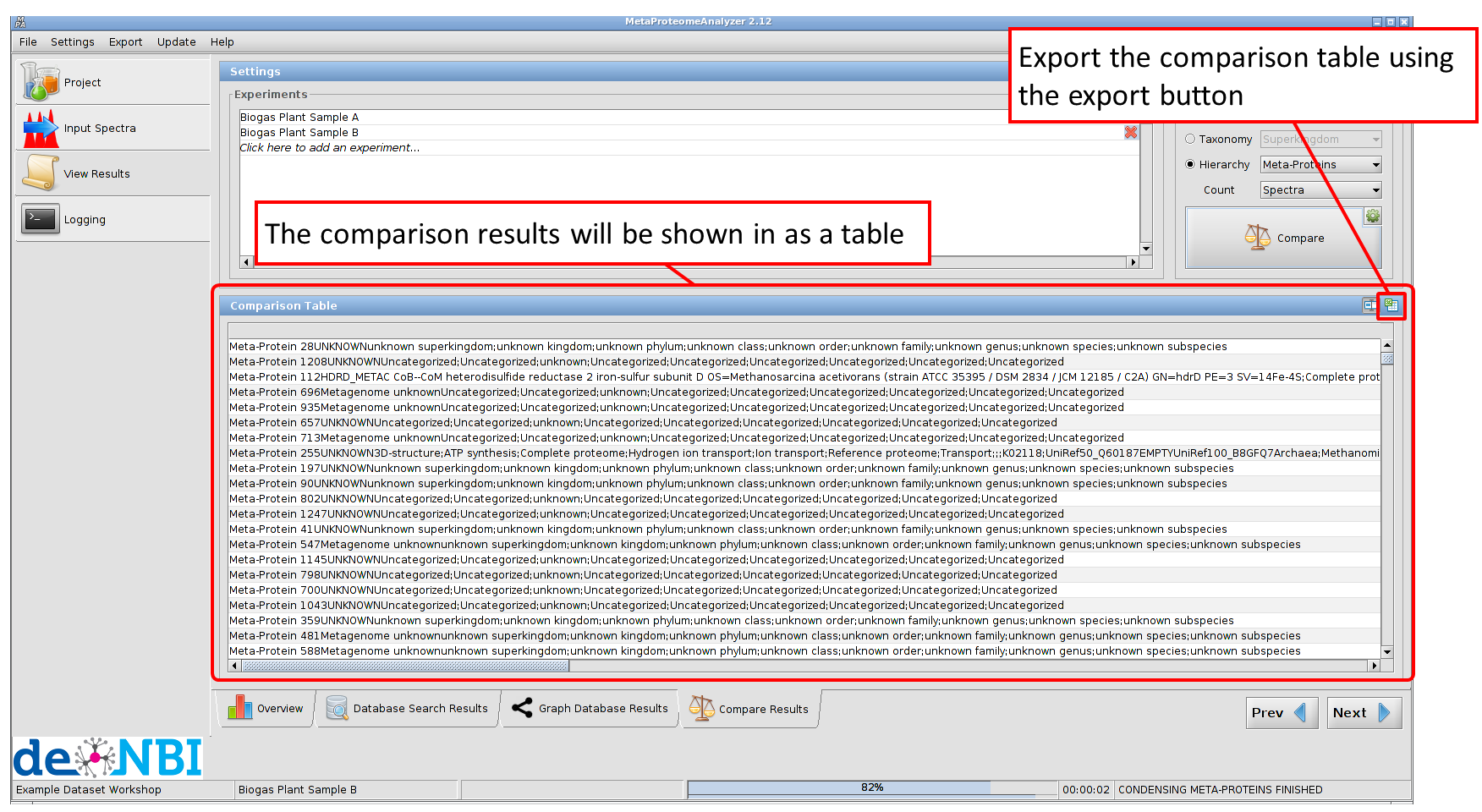


# Metaprotein concept

Metaproteins are protein groups that consider the special use case of metaproteomics. In order to deal with homologous proteins, which are expected in a multi-species system, proteins are grouped into metaproteins using a set of rules. The metaprotein will then be assigned a taxonomy based on the proteins that it contains depending on the specification the user provides. Unlike protein groups used by other proteomics tools, metaproteins should not be considered a single protein with an ambiguous identification, but instead they constitute a group of related proteins all of which are potentially contained in the sample. From this it follows that metaproteins will sometimes be assigned apparently unspecific taxonomies (i.e. Superkingdom rank), which indicates that the protein sequences on which the metaprotein is based are highly conserved across different taxa, making a specific taxonomic assignment impossible in a microbial community of multiple unknown species. Metaproteins will also combine other metadata from its proteins into a single entry: UniProt Keywords, UniRef Clusters, KEGG Orthologies and enzyme commission numbers (EC).

Metaproteins will be created according to the rules the user chooses. All three rules can be combined in any combination. The three rules are: 1. Peptide Rule, 2. Cluster Rule and 3. Taxonomy Rule as seen in Figure 1. Table 1 shows all available options and gives a description of how it will affect the metaprotein generation.


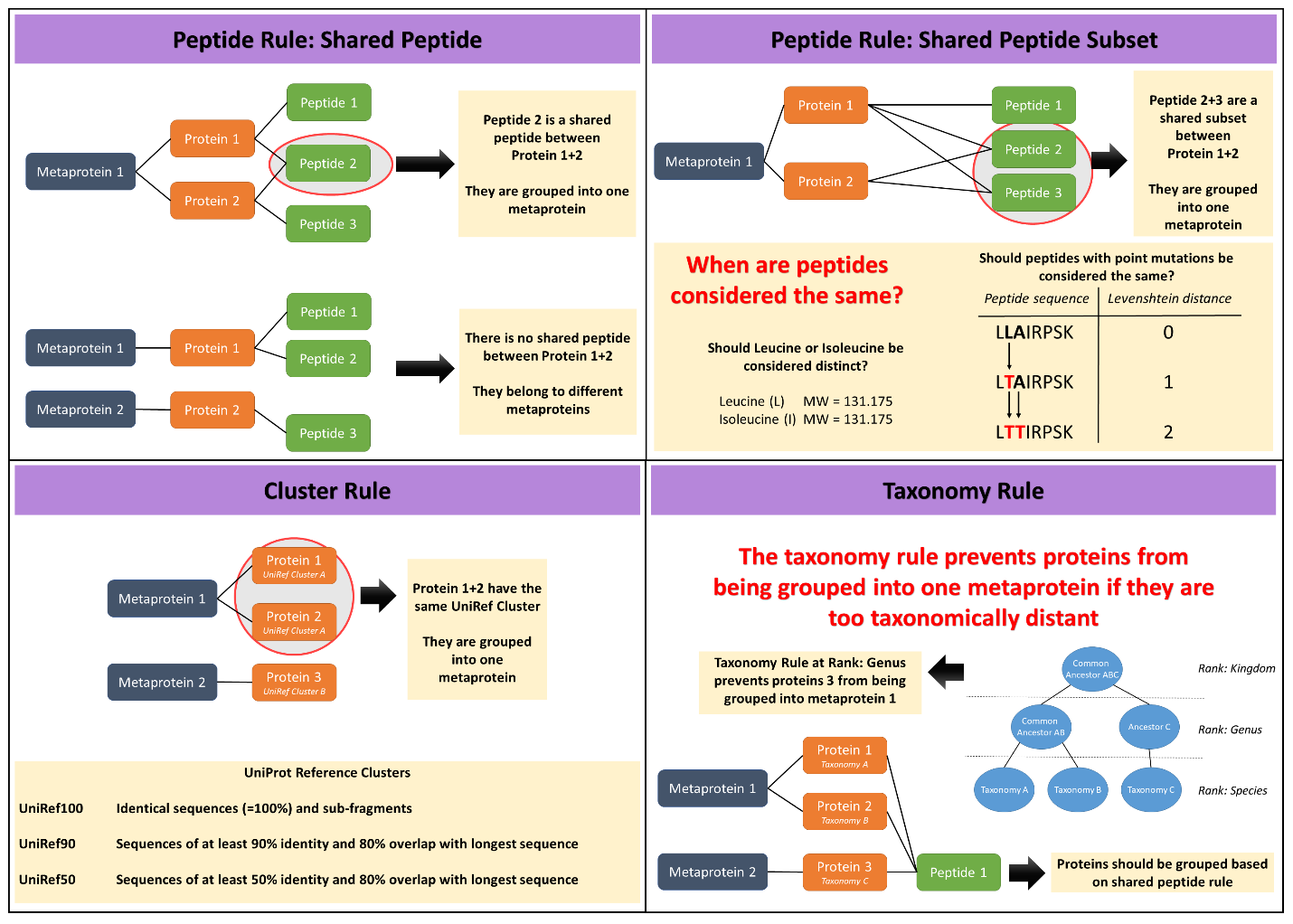


Figure 1: Metaprotein Rules. Different rules can be applied to determine how proteins are grouped together into metaproteins: 1. Peptide Rule, 2. Cluster Rule, 3. Taxonomy Rule.

Table 1: List of metaprotein rules and other options.

| **Metaprotein Rule** | **Description** |
| --- | --- |
| Peptide Rule: Shared Peptide | Two proteins will be considered for one metaprotein if they have at least one peptide in common. Using this rule, two proteins of a metaprotein may have no peptides in common if they share a peptide with a third protein. |
| Peptide Rule: Shared Peptide Subset | Two proteins will be considered for one metaprotein if they share a common set of peptides. This means that either both proteins contain the exact same set of peptides or if they share all the same peptides where one protein may have fewer peptides from the total set. Using this rule, two proteins will not be grouped if both possess unique peptides. |
| Peptide Rule: Leucine/Isoleucine | Since Leucine and Isoleucine have the same molecular weight, they are considered to be indistinguishable by mass spectrometry. This option will either consider peptides that only differ in these amino acids equal or distinct for the purpose of other peptide rules. |
| Peptide Rule: Levenshtein distance | The Levenshtein distance measures the number of single amino acid substitutions between two peptide sequences. Using this rule, peptides with the Levenshtein distance that are set by the user will be considered equal for the purpose of other peptide rules. |
| Cluster Rule: UniRef100 | Using this Cluster Rule, proteins will be grouped into a metaprotein if they belong to the same UniRef100 cluster. |
| Cluster Rule: UniRef90 | Using this Cluster Rule, proteins will be grouped into a metaprotein if they belong to the same UniRef90 cluster. This will always include all proteins that also share the UniRef100 cluster. |
| Cluster Rule: UniRef50 | Using this Cluster Rule, proteins will be grouped into a metaprotein if they belong to the same UniRef50 cluster. This will always include all proteins that also share the UniRef90 and UniRef100 cluster. |
| Taxonomy Rule | The taxonomy rule will prevent two proteins from being grouped into a metaprotein if they are not taxonomically close enough. In this option the highest taxonomic rank is chosen for which proteins are still grouped into a metaprotein. This rule does not work on its own and has to be used together with the peptide or cluster rule. |
| Peptide-to-Protein Taxonomy | Two options are available to determine in which way protein taxonomies are redefined based on the peptide taxonomy: lowest common ancestor (LCA) or most specific member. LCA will find the lowest common ancestor taxonomy (up to “root”) to which all peptides of this protein belong. Most specific member will select the first taxonomy of those peptide taxonomies with the lowest rank (i.e. sup-species). |
| Protein-to-Metaprotein Taxonomy | Similarly, two options are available to determine in which way metaprotein taxonomies are generated based on the protein taxonomy: lowest common ancestor (LCA) or most specific member. LCA will find the lowest common ancestor taxonomy (up to “root”) to which all proteins of this metaprotein belong. Most specific member will select the first taxonomy of those protein taxonomies with the lowest rank (i.e. sup-species). |


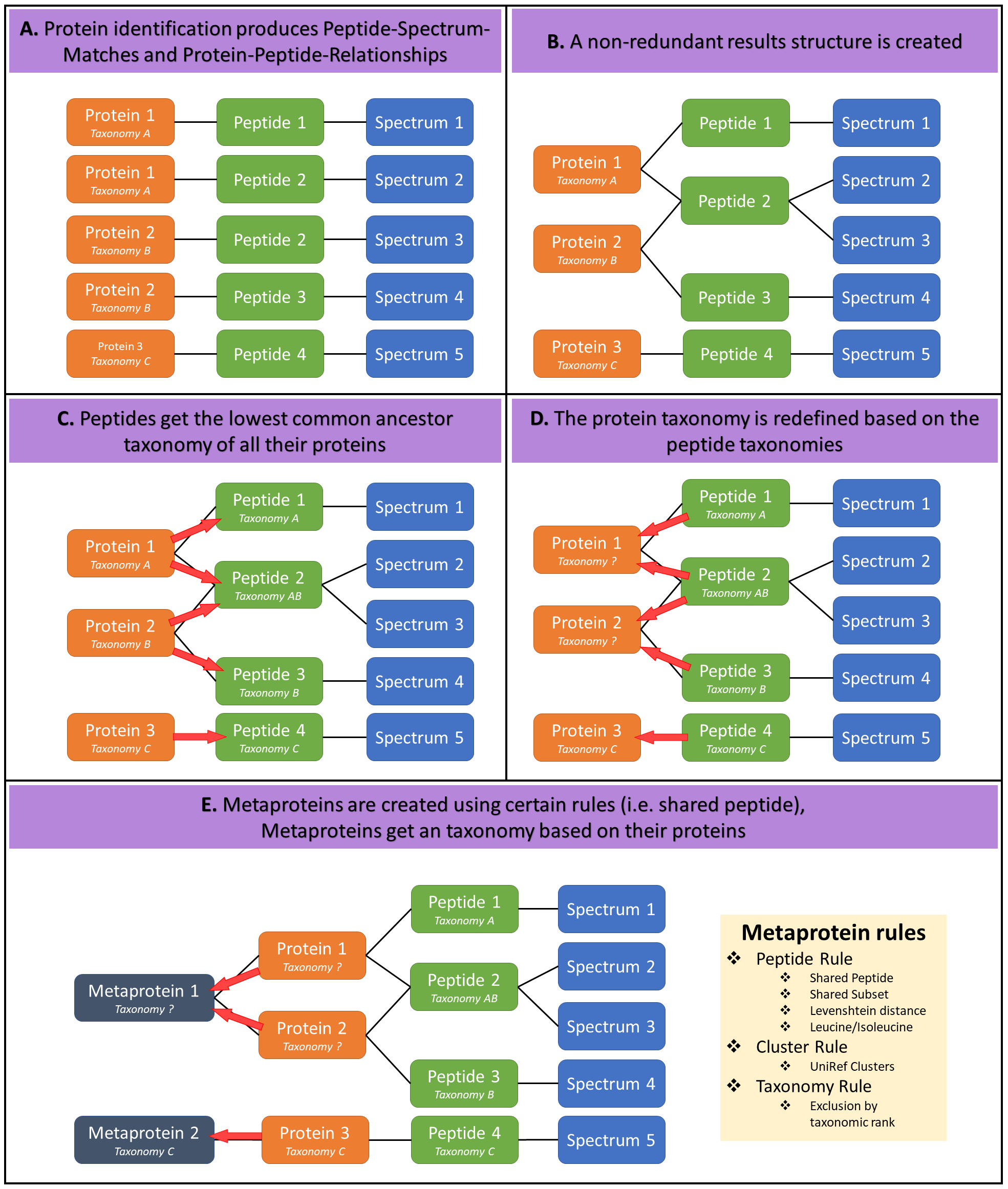


Figure 2: Metaprotein Taxonomy.The five main steps A-E are followed, when creating metaproteins to determine the taxonomy of the metaprotein. The “Protein-to-Peptide” (C) taxonomy is set to be the lowest common ancestor taxonomy (LCF). The “Peptide-to-Protein” (D) and “Protein-to-Metaprotein” (E) taxonomies can be set to LCA or “most specific member” independently of each other.
