# Supplementary material for: A Robust and Universal Metaproteomics Workflow for Research Studies and Routine Diagnostics Within 24 h Using Phenol Extraction, FASP Digest, and the MetaProteomeAnalyzer
Source: Front Microbiol. 2019 Aug 16;10:1883. doi: 10.3389/fmicb.2019.01883 (PMC6707425; doi:10.3389/fmicb.2019.01883)
Supplement: TABLE S10 — Additional visualizations. [file Table_10.DOCX]

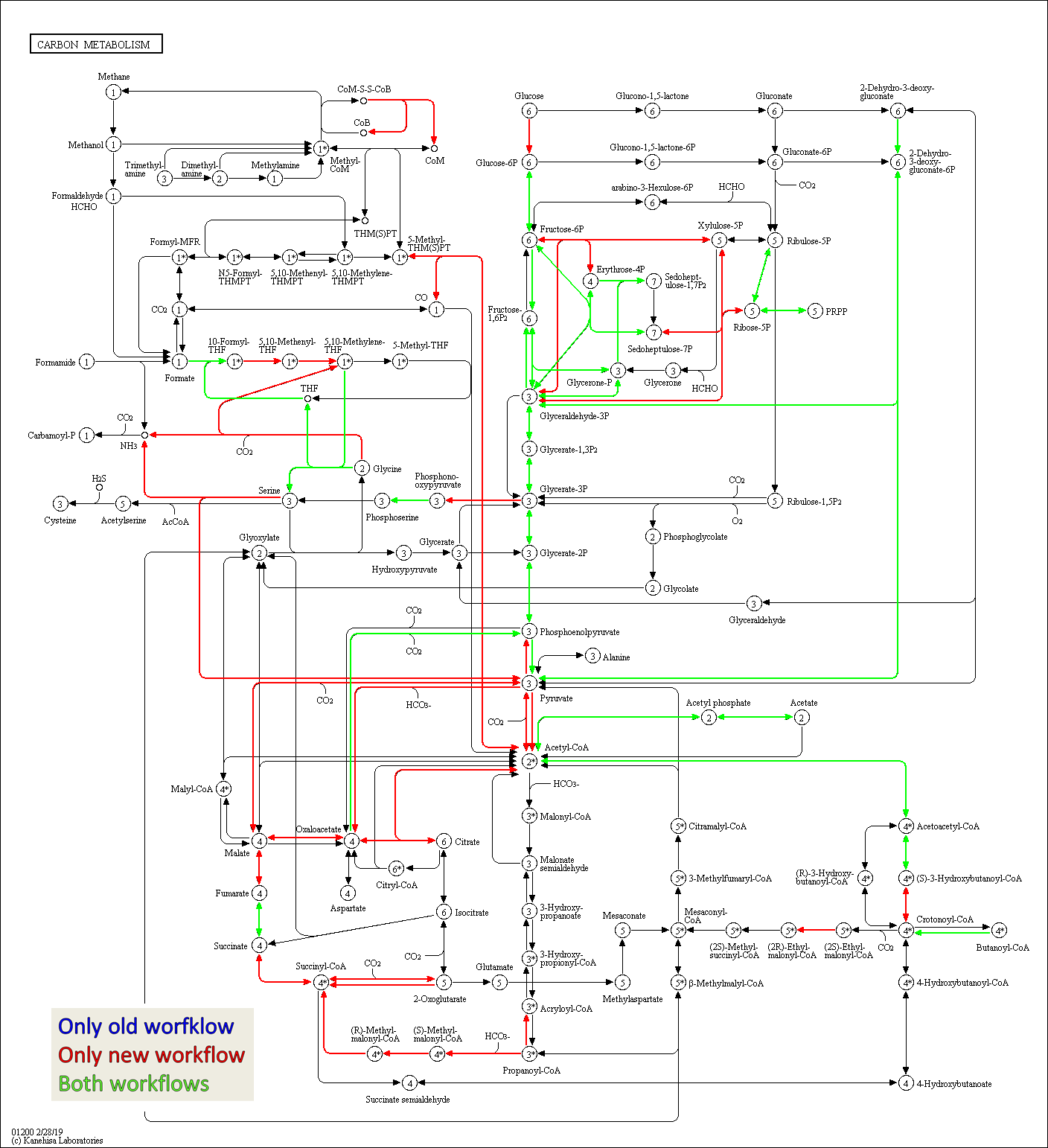


**KEGG map carbon metabolism Hgut_1 Old Workflow vs. New Workflow**

This is the KEGG map for the carbon metabolism showing enzymes in the sample Hgut_1 (3 technical replicates combined). The map is colored to highlight differences between functional annotation, where blue are KO numbers exclusively found in the analysis with the old laboratory workflow, red are KO numbers exclusively found in the analysis with the new laboratory workflow and green are KO numbers found with both. The maps are also hosted on: <http://www.mpa.ovgu.de/review/kegg_carbonmetabolism_Hgut_1.png>


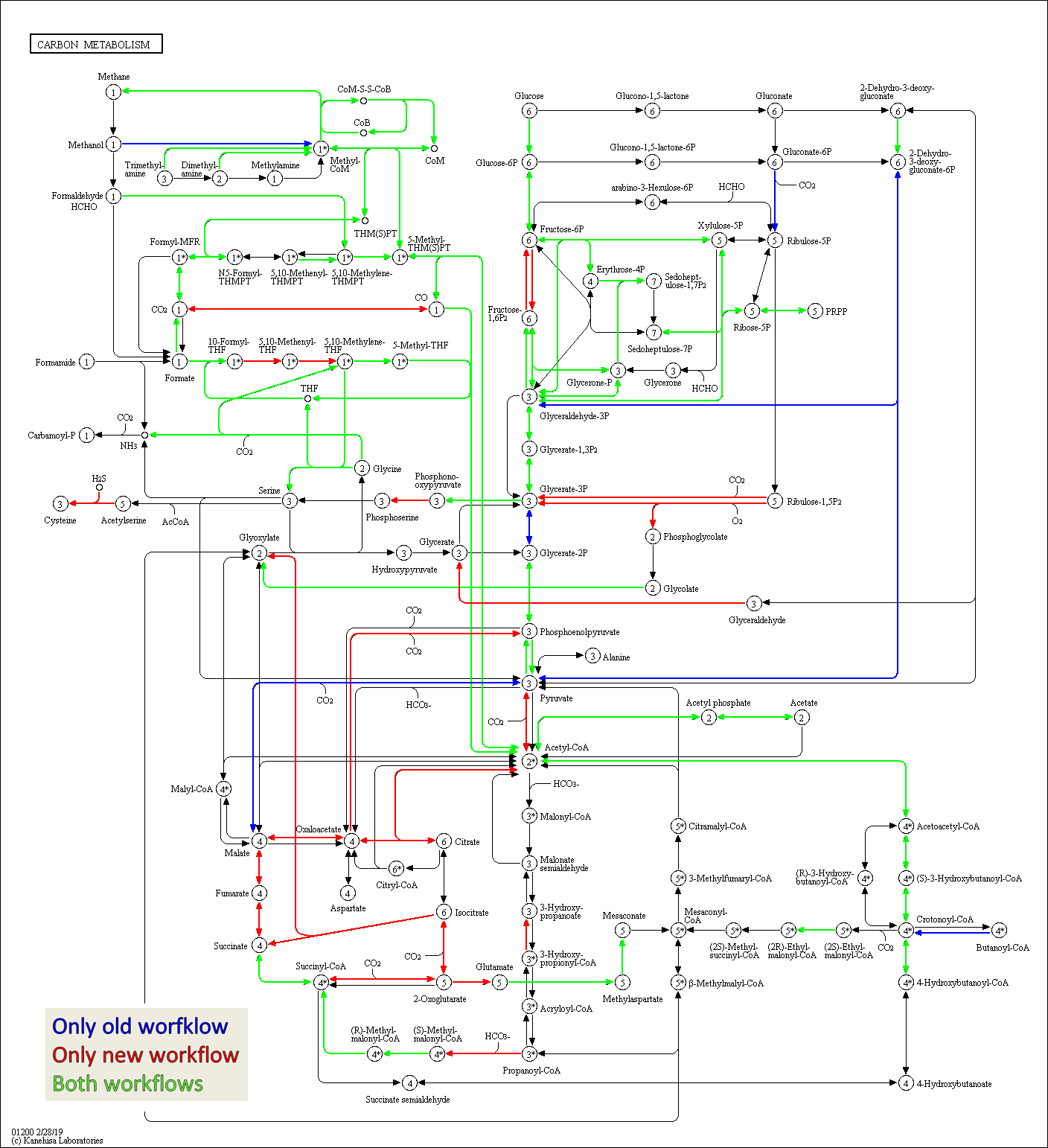


**KEGG map carbon metabolism BGP_1, Old Workflow vs. New Workflow**

This is the KEGG map for the carbon metabolism showing enzymes in the sample BGP_1 (3 technical replicates combined). The map is colored to highlight differences between functional annotation, where blue are KO numbers exclusively found in the analysis with the old laboratory workflow, red are KO numbers exclusively found in the analysis with new laboratory workflow and green are KO numbers found with both. The maps are also hosted on: <http://www.mpa.ovgu.de/review/kegg_carbonmetabolism_BGP_1.png>

**Krona Hgut_1 New Workflow**


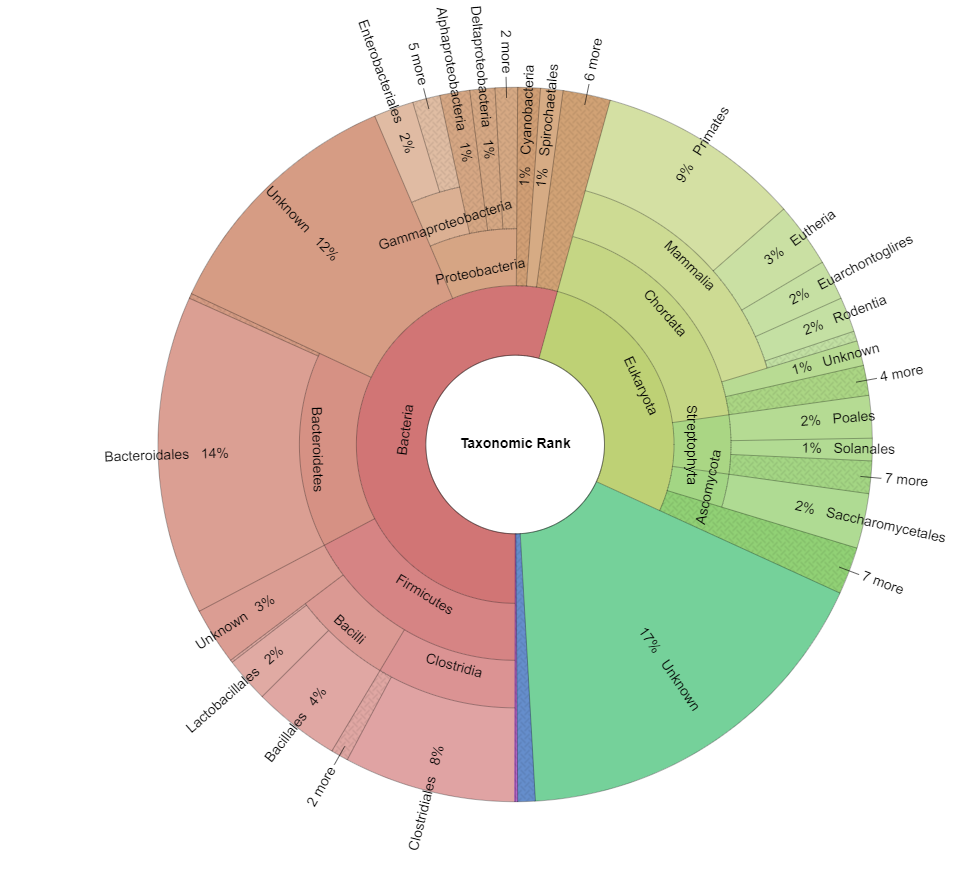


**Krona Hgut_1 Old Workflow**


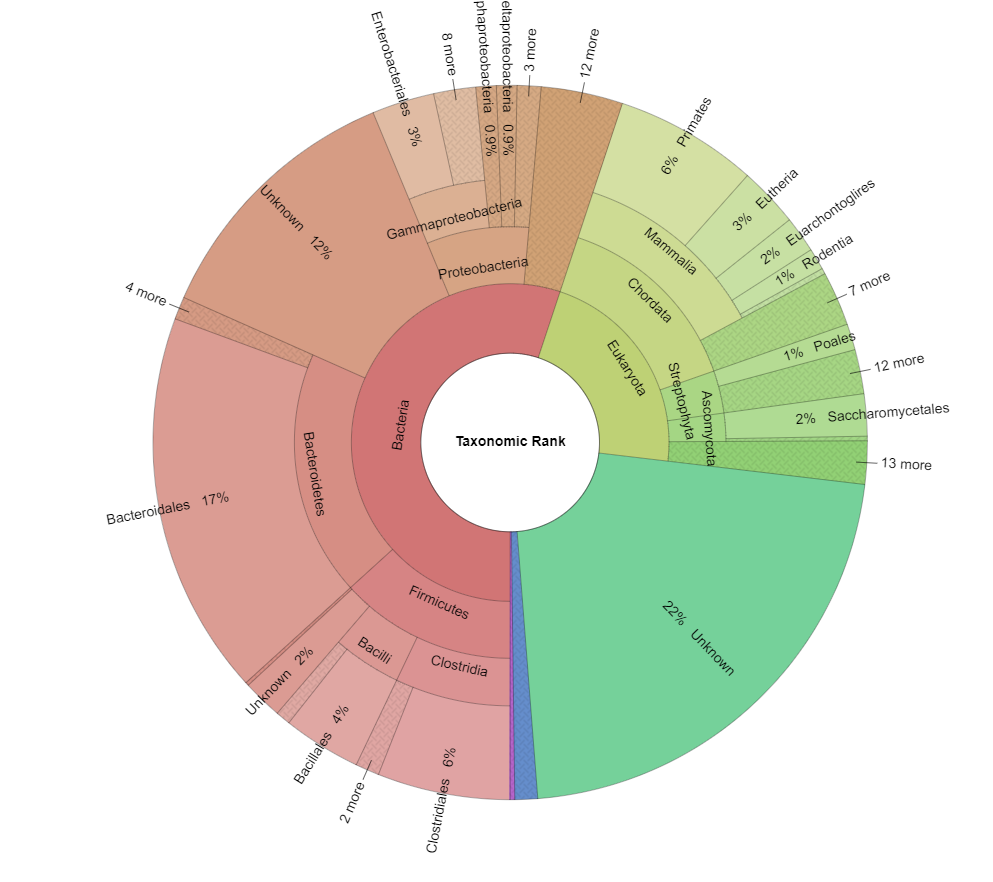


Seen are two snapshots of a comparative Krona Plot for sample Hgut_1 (old vs. new workflow). The Krona plots highlight that taxonomies only change slightly between different old and new workflow, although the number of spectra used is drastically different. The interactive graph is hosted on: <http://www.mpa.ovgu.de/review/comparative_KronaPlot_Hgut_1.html>

**Krona BGP_1 New Workflow**


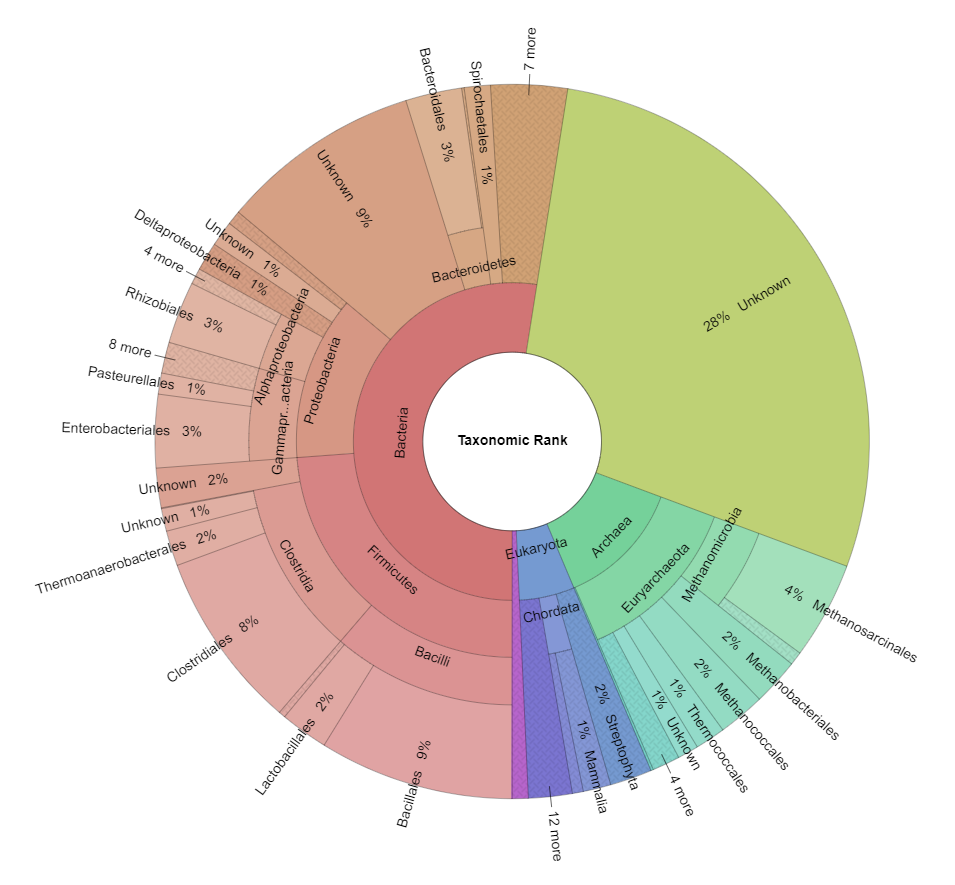


**Krona BGP_1 Old Workflow**


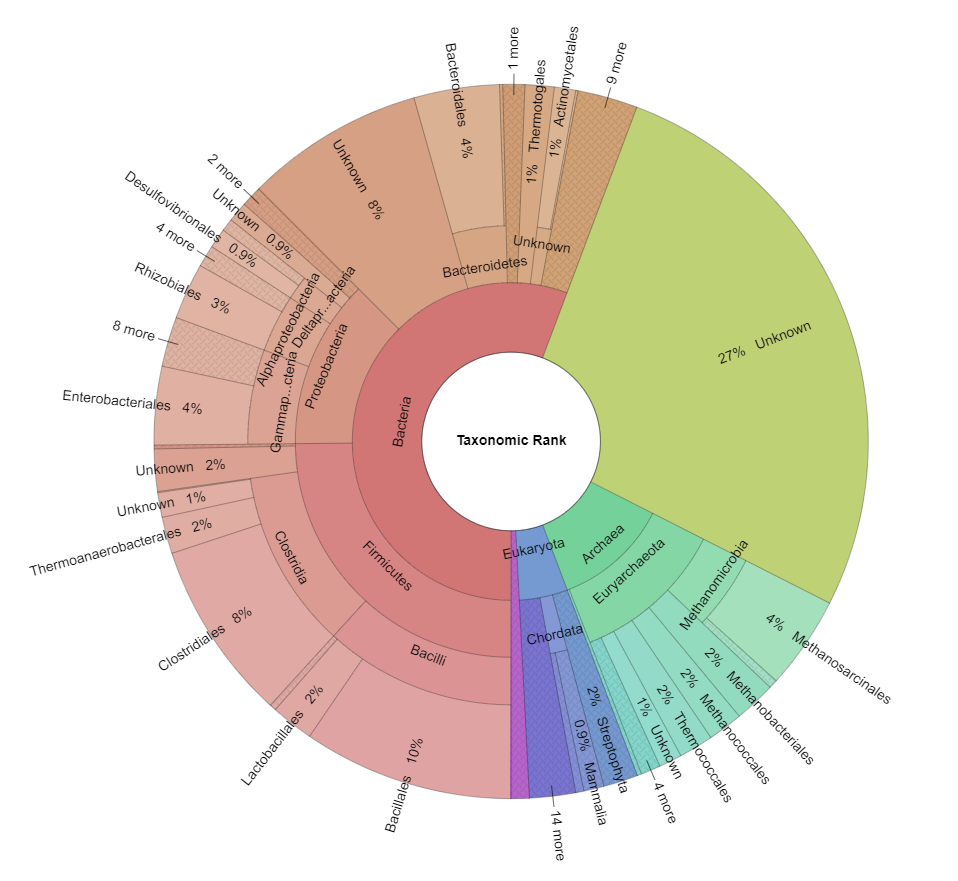


Seen are two snapshots of a comparative Krona Plot for sample BGP_1 (old vs. new workflow). The Krona plots highlight that taxonomies only change slightly between different old and new workflow, although the number of spectra used is drastically different. The interactive graph is hosted on: <http://www.mpa.ovgu.de/review/comparative_KronaPlot_BGP_1.html>

We also created two interactive chord diagrams for the samples Hgut_1 and BGP_1, available at the addresses below. The elements that are shown can be changed using the tables below the chart. Hovering over certain elements highlights relevant parts of the chart. Using the remote server, these interactive charts can be created directly in the MPA (which opens the browser with the created chart).

**Chord Hgut_1** (interactive diagram: <http://www.mpa.ovgu.de/human_gut.html>)


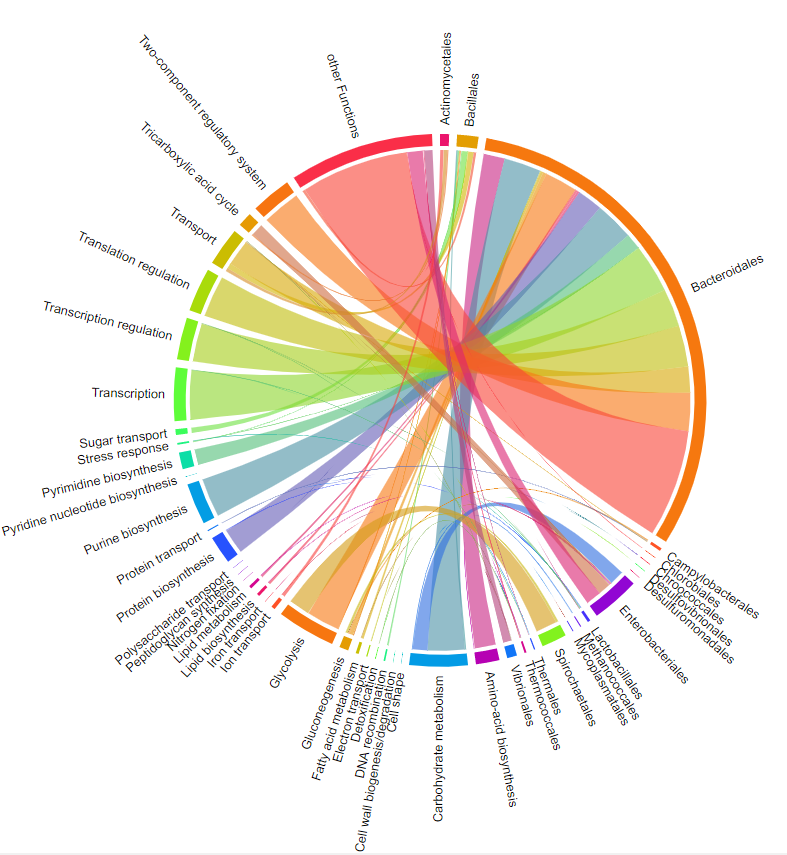


This is a snapshot chord diagram for Hgut_1, where an appropriate selection of taxonomies and functions was taken.

**Chord BGP_1** (interactive diagram: <http://www.mpa.ovgu.de/biogas_plant.html>)


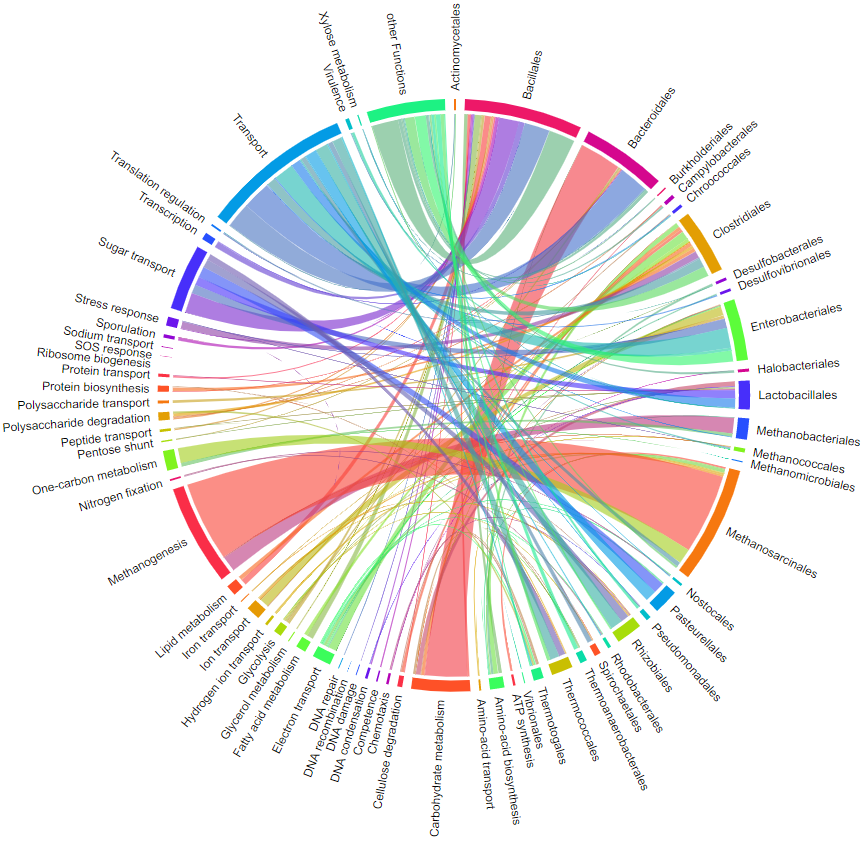


This is a snapshot chord diagram for BGP_1, where an appropriate selection of taxonomies and functions was taken.
