# Supplementary material for: A Robust and Universal Metaproteomics Workflow for Research Studies and Routine Diagnostics Within 24 h Using Phenol Extraction, FASP Digest, and the MetaProteomeAnalyzer
Source: Front Microbiol. 2019 Aug 16;10:1883. doi: 10.3389/fmicb.2019.01883 (PMC6707425; doi:10.3389/fmicb.2019.01883)
Supplement: DATA SHEET S1 — Collection SOPs. [file Data_Sheet_1.PDF]

**Date:** 10/5/2019		Otto-v.-Guericke-University Magdeburg
**Creator:** Robert Heyer		Bioprocess Engineering
**Person responsible:** Dr. Benndorf		Universitätsplatz 2
**Update:** ----		39106 Magdeburg
**Department:** BPT	**Phenol extraction**	**Date:**
**Version:** 1.0.0.		**Signature:**

SOP: Phenol extraction

Application:

This protocol enables the extraction of proteins from environmental samples. Extraction protocol is applicable also for samples containing high amounts of impurities, e.g. humic substances.

Remarks:

- Pay attention while working with phenol. It is toxic!
- Dispose contaminated materials and liquid waste into special containers. Check sucrose solution for any contaminations (flocculation). Urea buffer could crystallize during storage at 4°C. Hence, stir before usage.
- All incubation steps at -20 °C could be extended overnight in order to make a break.

Chemicals

Table 1: Used chemicals. Named manufacturer as well as order numbers are not mandatory and can be replaced by others with equal quality.

Chemicals	Manufacturer	Order Numbers	Storage place
Ammonium acetate	Roth Art.	7869.1	
1,4-Dithiothreitol (DTT)	Carl Roth	6908.2	
Methanol, ≥ 99.9% (**toxic** ☠☠☠)	Carl Roth	T909.1	
Phenol (**toxic** ☠☠☠**)**	Merck	8.22296.1000	
Succrose	Merck	1.07687.1000	
Thiourea (**toxic** ☠☠☠**)**	Sigma-Alderich	T8656	
Urea	AppliChem	A1049.0500	
0.5mm Zirconia/ Silica Beads	BioSpec Products Inc	1179105z	

Devices

Table 2: Used devices. Devices and manufacturers are not mandatory and can be replaced by others with equal functionality.

Device	Name	Manufacturer
Ball mill	FastPrep-96	MP Biomedicals (Eschwege, Deutschland)
Destilled water	Millipore Q-POD	Merck, Darmstadt, Germany
Centrifuge	Micro Star 17 R	VWR International, Darmstadt, Germany
Centrifuge	Avanti J	Beckman Coulter, Brea, USA

Buffer preparation

1,000% Phenol Solution:	10 g phenol (**toxic** ☠☠☠)<br>1 mL dest. water<br>(storage at 4 °C under the fume hood!)
0.1 M Ammonium Acetate in Methanol:	0.7708 g ammonium acetate<br>Fill up to 100 mL with methanol (storage at -20 °C)
2 M Sucrose:	68.46 g sucrose<br>Fill up to 100 mL with dest. water (storage at 4 °C)
1 M Sucrose / Saccharose Solution:	34.23 g Sucrose<br>Fill up to 100 mL with dest. water (storage at 4 °C)
Urea Buffer:	8.41 g urea (7 M)<br>3.04 g thiourea (2 M) (**toxic** ☠☠☠)<br>0.2 g DTT (0.01 g/mL)<br>Fill up to 20 mL with dest. water<br>(storage at 4 °C)

Execution:

FASTPREP

- Weight out 5 g silica-beads into a 50 ml reaction tube
- Add 2 g resp. 2 mL sample
- Add 2 mL 2 M sucrose solution
- Add 3.5 mL phenol
- Close reaction tube (e.g. you may add also wrap the cap with parafilm)
- Place reaction tubes into the FASTPREP
- Cell lysis at 1,800 rpm for 5 min
- 10 min centrifugation with 10,000 g and RT
- Take upper phenol phase and collect it in a new 50 mL reaction tube
- Add same volume 1 M sucrose solution
- Shake (10 min, 60- 120 rpm, RT)
- Centrifuge (10 min, RT, 10,000 xg)
- Transfer upper phenol phase into a new 50 mL reaction tube
- Add the four-fold volume of icecold (4v/v) ammoinium acetate in methanol
- Incubate for 20 min at –20 °C,

- Centrifuge (10 min, 10,000 xg, 4 °C)
- Add the four-fold volume of icecold (4v/v) ammoinium acetate in methanol
- Incubate for 20 min at –20 °C,
- Centrifuge (10 min, 10,000 xg, 4 °C)
- Discard supernatant

Ball mill (small 2 ml reaction tubes)

- Weight out 1 g silica-beads into a 2 ml reaction tube (**five times**)
- Add 400 mg/ µL sample
- Add 400 µL 2 M sucrose solution
- Add 700 µL phenol
- Place reaction tubes into the ball mill
- Cell lysis at 30 hertz for 10 min
- 10 min centrifugation with 10,000 g and RT
- Take upper phenol phase and pool them in a 15 mL reaction tube
- Add same volume 1 M sucrose solution
- Shake (10 min, 60- 120 rpm, RT)
- Centrifuge (10 min, RT, 10,000 xg)
- Transfer upper phenol phase into a new 15 mL reaction tube
- Add the four-fold volume of icecold (4v/v) ammonium acetate in methanol
- Incubate for 20 min at –20 °C,
- Centrifuge (10 min, 10,000 xg, 4 °C)
- Discard supernatant
- Add the four-fold volume of icecold (4v/v) ammonium acetate in methanol
- Incubate for 20 min at –20 °C,
- Centrifuge (10 min, 10,000 xg, 4 °C)
- Discard supernatant

Dissolve sample

- Dry pellet
- Dissolve pellet in e.g. 1 mL urea buffer and 0.1 g Silica Beads
- 2 min ball mill/ FASTPREP
- 5 min centrifugation with 10,000 xg and RT
- transfer supernatant into a new 2 mL reaction tube
- store at -20 °C

Encloser:

- not applicable

Abbreviations:

dest. water	deionized water
DTT	dithiothreitol
M	molare
RT	room temperature

6.9 SOP FASP Digest

Application:

Sample preparation of cell cultures for LC-MS/MS measurement, according to following references [1,2].

1. Wisniewski, J. R., Zougman, A., Nagaraj, N., and Mann, M. 2009. Universal sample preparation method for proteome analysis. Nature Methods, 6(5):359–362
2. Wiśniewski, J.R., Hein M.Y., Cox J., Mann M. Mol Cell Proteomics. 2014 Dec;13(12):3497-506. A "proteomic ruler" for protein copy number and concentration estimation without spike-in standards.

Remarks:

- The method provides protein digest that are free from nucleic acids and other cell components
- The method can be applied to samples containing high concentrations of detergents
- There is no precipitation and the concentration of sample is kept high
- In a single filter device 0.2-200 µg of total protein can be processed. Thus, FASP can be used upstream of separations of peptides such as OFFGEL and 2D- LC
- The yield and purity of peptides can be monitored by UV-spectrometry allowing QC of the digest
- Do not mix up the different urea buffers!

Chemicals

Table 1: Used chemicals. Named manufacturer as well as order numbers are not mandatory and can be replaced by others with equal quality.

Chemicals	Manufacturer	Order Numbers	Storage place
Acetic Acid≥ 96% (**corrosive!!!**)	Carl Roth	T179.2	
BSA (control)	Sigma-Alderich	A3912	
1,4-Dithiothreitol (DTT)	Carl Roth	6908.2	
Methanol, ≥ 99.9% (**toxic** ☠☠☠)	Carl Roth	T909.1	
Thiourea (**toxic** ☠☠☠**)**	Sigma-Alderich	T8656	
Urea	AppliChem	A1049.0500	
Centrifugal Filter Units (MWCO 10 kDa)	Pall Nanosep 10K Omega	OD010C35	
Acetonitrile (MS/MS grade)	Fluka	34967	
Iodacetamide	AppliChem,	A1666.0025	
Ammoniumbicarbonat ultra >99.5%	Fluka	09830	
Trypsin, Premium grade	Serva	111095	

Formic acid 97%	Fluka	5602	
Triflouressigsäure	Sigma Life Science	73645	
MilliQ	MilliQ, LC-MS Grade or MilliQ		
Ethanol 96% (not denaturedt)	Merck	1,009,832,511	

Devices

Table 2: Used devices. Devices and manufacturers are not mandatory and can be replaced by others with equal functionality.

Device	Name	Manufacturer
Speed Vac	Digital Series SpeedVac SPD121P	Thermo Scientific, Waltham, USA
Destilled water	Millipore Q-POD	Merck, Darmstadt, Germany
Centrifuge	Micro Star 17 R	VWR International, Darmstadt, Germany
Centrifuge	Avanti J	Beckman Coulter, Brea, USA

Buffer preparation

Urea Buffer:	8.41 g urea (7 M)<br>3.04 g thiourea (2 M) (**toxic** ☠☠☠)<br>0.2 g DTT (0.01 g/mL)<br>Fill up to 20 mL with dest. water<br>(storage at 4 °C)
Ammonium bicarbonate buffer 50 mM, pH 7.8	98.80 mg NH_4HCO_3 (ABC)<br>25 ml dest. water<br>Prepare the buffer immediately before use.<br>Keep it at room temperature until use.
5 M NaOH	20 g NaOH<br>100 ml dest. water
0.1 M Tris-HCl pH 8.5	15.7 g Tris-HCl<br>800 mL dest. water<br>Adjust ph to 8.5 by adding 5 M NaOH<br>Fill up to 1 L
8 M Urea buffer	4.8 g Urea

	10 ml 0.1 M Tris-HCl pH 8.5<br>Fill up to 10 ml dest. water
0.40 M DTT Stock solution	24.68 mg DTT (M=154.25 g/mol)<br>400 µl 50 mM NH_4HCO_3<br>Prepare immediately before use<br>(store in the dark)
40 mM DTT Working Solution	Dilute 0.4 M DTT Stock solution 10fold with 8 M urea buffer<br>Immediately before use<br>(store in the dark)
0.55 M IAA Stock Solution	40.7 mg IAA (M=184.96 g/mol)<br>400 µl 50 mM NH_4HCO_3<br>Prepare immediately before use<br>(store in the dark)
55 mM IAA Working Solution	Dilute 0.55 M IAA Stock solution 10fold with 8 M urea buffer<br>Prepare immediately before use<br>(store in the dark)
Trypsin (25 µg/25 µl)	resuspend lyophilized Trypsin (Serva) in 25 µl 50 mM acetic acid c = 1 µg/µl<br>optional: 1 mM $(CaCl_2)aq$
Digestion buffer	1 ml 50 mM ABC buffer + 5% Acetonitrile (50 µl)

Execution:

A. FASP-DIGEST

- Add sample on the filter in maximum 200 µL of **urea buffer** vortex and incubate for 5 min on a thermomixer (recommended 100 µg)
- Transfer the protein solution to the filter unit (min vol. 50 µL / max vol. 500 µL)
- Centrifuge, 10,000 xg, 10 min at RT (**in case of remaining liquid on top of the filter extend centrifugation time!**)
- Add 200 µL of **urea buffer**
- Centrifuge, 10,000 xg, 10 min at RT (**in case of remaining liquid on top of the filter extend centrifugation time!**)

- Add 100 µL **DTT solution** to the filter unit, mix for 1 min at 800 rpm using a thermomixer, incubate at 56°C for 20 min with gentle agitation (300 rpm)
- Centrifuge, 10,000 xg, 5 min at RT,
- Add 100 µL **IAA solution** to the filter unit, mix for 1 min at 800 rpm using a thermomixer, Incubate for 20 min in the dark at RT
- Centrifuge, 10,000 xg, 5 min at RT
- Wash 3x with 100 µL **urea buffer**: incubate for 2 min at RT on a thermomixer, centrifuge for 5 min
- Wash 3x with 100 µL 50 mM **ABC buffer**: incubate for 2 min at RT on a thermomixer, centrifuge for 5 min
- Discard flow-through

Tryptic Digest

- Prepare enzyme solution (Trypsin in 50mM ABC buffer; 200 µL/sample recommended)
    - **Recommended enzyme:substrate ratio (E:S):** 1:100 for Trypsin
- Add 200 µl enzyme solution
- Incubate at 37 °C for 2h with gentle agitation (350 rpm)

Peptide Extraction

- Centrifuge, 10,000 xg, 5 min at RT, **DO NOT discard the flow-through**
- Add 50 µL **digestion buffer** (50mM ABC buffer + 5% Acetonitrile) to the filter unit
- Centrifuge, 10,000 xg, 5 min at RT, **DO NOT discard the flow-through**
- Add 50 µL **LC-MS grade dest. water** to the filter unit
- Centrifuge, 10,000 xg, 5 min at RT, **DO NOT discard the flow-through**
- Discard Filter Unit (Protein is in flow-through!)
- a.) acidify samples with TFA to same concentration as in the loading buffer of the MS and transfer the sample into an HPLC vial
- b.) as alternative you may dry the digest in a SpeedVac and store it at -20°C

For further preparation see protocols for MS measurements.

Encloser:

- NONE

Abbreviations:

BSA	bovine serum albumin
dest. water	deionized water
DMEM	Dulbecco`s modified eagle medium
DTT	dithiothreitol
FCS	fetal calf serum

M	molar
IAA	iodoacetamide
RT	room temperature

**Date:** 11/4/2017<br>**Creator:** Robert Heyer<br>**Person responsible:** Dr. Benndorf<br>**Update:** **17/10/2018**		OTTO VON GUERICKE UNIVERSITÄT MAGDEBURG	Otto-v.-Guericke-University Magdeburg<br>Bioprocess Engineering<br>Universitätsplatz 2<br>39106 Magdeburg
**Department:** BPT<br><br>**Version:** 7.0.0.		**Protein quantification with amido black**	**Date:**<br><br>**Signature:**

SOP: Protein quantification with amido black

Application:

The amido black assay enables the protein quantification in environmental samples. The protocol is compatible with urea buffer, other detergents and contaminants (see also the publications [1,2]).

1. Popov, N., Schmitt, M., Schulzeck, S., and Matthies, H. 1975. Eine störungsfreie Mikromethode zur Bestimmung des Proteingehaltes in Gewebehomogenaten. *Acta Biologica et Medica Germanica*, 34:1441–1446.
2. Schweikl, H., Klein, U., Schindlbeck, M., and Wieczorek, H. 1989. A vacuolar-type atpase, partially purified from potassium transporting plasma membranes of tobacco hornworm midgut. *Journal of Biological Chemistry*, 264(19):11136–11142.

Remarks:

- Amido black crosslinks proteins and dye molecules, causing their precipitation.
- Pay attention to your pellet during washing. Then you dissolve your pellet check whether the complete pellet is dissolved.
- Protein for the standard curve has to be dissolve into the same buffer as the sample.
- As blank use 0.1 M sodium hydroxide.
- For absorption above >2.0 or outside the calibration curve the sample has to be diluted with sodium hydroxide.

Chemicals

Table 1: Used chemicals. Named manufacturer as well as order numbers are not mandatory and can be replaced by others with equal quality.

Chemicals	Manufacturer	Order Numbers	Storage place
Acetic Acid≥ 96% (**corrosive!!!**)	Carl Roth	T179.2	
BSA	Sigma-Alderich	A3912	
1,4-Dithiothreitol (DTT)	Carl Roth	6908.2	
Methanol, ≥ 99,9% (**toxic** ☠☠☠)	Carl Roth	T909.1	
Naphtol Blue Black 80 % (m/v) Dye Content (Amido Black)	Sigma-Alderich	195243	
Sodium hydroxide, NaOH ≥ 98% (**corrosive!!!**)	Carl Roth	P031.1	
Thiourea (**toxic** ☠☠☠)	Sigma-Alderich	T8656	
Urea	AppliChem	A1049.0500	

Devices

Table 2: Used devices. Devices and manufacturers are not mandatory and can be replaced by others with equal functionality.

Device	Name	Manufacturer
Photometer	Spectrophotometer Genesys 10S UV Vis	VWR International, Darmstadt, Germany
Destilled water	Millipore Q-POD	Merck, Darmstadt, Germany
Centrifuge	Micro Star 17 R	VWR International, Darmstadt, Germany
Centrifuge	Avanti J	Beckman Coulter, Brea, USA

Buffer preparation

Urea Buffer:
8.41 g urea (7 M)
3.04 g thiourea (2 M) (**toxic** ☠☠☠)
0.2 g DTT (0.01 g/mL)
Fill up to 20 mL with destilled water
(storage at 4 °C)

Wash solution:
180 mL methanol (**toxic** ☠☠☠)
20 mL acetic acid
(storage at RT)

Dye solution:
26 mg amido black
Add 100 mL wash solution
(storage at RT)

0.1 M Sodium hydroxid
0.4 g sodium hydroxyden
Fill to 100 mL with destilled water

Execution:

Measure the standard curve for BSA as triplicates:

- 0.0 µg/µL (just sample buffer)
- 0.2 µg/µL
- 0.4 µg/µL
- 0.6 µg/µL
- 0.8 µg/µL
- 1.0 µg/µL

Prepare and measure the sample at least as triplicates. For samples with an expected high concentration use 1:5 dilutions (4 parts buffer and 1 part sample). For example biogas plant samples and waste water treatment plant samples.

- put 50 µL sample/ standard into a 1.5 ml reaction tube
- add 300 µl dye solution, vortex and centrifuge (16,400 g, 5 min, RT)
- Remove supernatant
- Add 500 µL Washing solution, vortex and centrifuge (16,400 g, 5 min, RT)
- Remove supernatant
- Add 500 µL Washing solution, vortex and centrifuge (16,400 g, 5 min, RT)
- Remove supernatant
- Dissolve pellet in 1 mL 0.1 M sodium hydroxide
- Place into a cuvette
- Measure at 615 nm wavelength
- Calculate sample concentration from standard curve

Encloser:

- Excel-Sheet

Abbreviations:

BSA	bovine serum albumin
dest. water	deionized water
DTT	dithiothreitol
M	molare
RT	room temperature

SOP: SDS-PAGE

Application:

SDS-PAGE enables the protein separation for quality control. Protocol is according to *Laemmli et al. [1].*

1. Laemmli, U. K. (1970). "Cleavage of structural proteins during the assembly of the head of bacteriophage T4." Nature **227**(5259): 680-685.

Remarks:

- Acrylamide and mercaptoethanol are toxic. Do all steps under a fume hood and wear glasses.
- For pH adjustment of Tris-HCl-solutions different HCl concentrations could be used. However, to low concentrations result in high volumes, which may exceed the maximum volume.
- Check whether you want to work with 1 mm or 1.5 mm thick gels and use corresponding glass plates, combs and gel volumes. The current during electrophoresis could be set below the maximum current. Also duration of fixation or dyeing could be extended.
- Note down LOT number of the respective protein standard used since LOT specific molecular weights may vary compared to the LOT independent molecular weights (see figure below
- The maximum/optimum loading volume per pocket for 1 mm gels is 30 µL/25 µL, 45 µL/40 µL for 1.5 mm gels, respectively.

Chemicals

Table 1: Used chemicals. Named manufacturer as well as order numbers are not mandatory and can be replaced by others with equal quality.

Chemicals	Manufacturer	Order Numbers	Storage place
Acetic Acid ≥ 96%	Carl Roth	T179.2	
Acetone, ≥ 99%.,	VWR International	20063.296	
Acrylamide solution 30% (toxic ☠☠☠)	Serva	10688.01	
Ammonium sulfate	Carl Roth	3746.3	
Ammonium persulfate (APS)	Merck	1.01201.0500	
Bromophenol blue	GE Healthcare	17-1329-01	
Coomassie BB G 250	AppliChem	A3484.0100	
Ethanol (70%)	Carl Roth	T913.3	
Glycerol	Carl Roth	3783.1	
Glycine	VWR International	444495D	
Hydrochloric acid (37%)	VWR International	20252.420	
Isobutanol	AppliChem	A1150.1000	
Mercaptoethanol (toxic ☠☠☠)	Sigma	M3148	
Methanol ≥ 99.9% (toxic ☠☠☠)	Carl Roth	T909.1	
Protein standard	Thermo Scientific PageRuler Prestained	Protein Ladder #26616	
Phosphoric acid ≥ 85%	Carl Roth	6366.2	

SDS (sodium dodecyl sulfate,(**toxic** ☠☠☠)	AppliChem	A2572.0250	
TEMED	Carl Roth	2367.1	
Tris	AppliChem	A2264.1000	

Devices

Table 2: Used devices. Devices and manufacturers are not mandatory and can be replaced by others with equal functionality.

Device	Name	Manufacturer
Electrophoresis chamber	Mini-Protean Tetra System	BioRad (Hercules, USA)
Power supply	PowerPac Basic	BioRad (Hercules, USA)
Destilled water	Millipore Q-POD	Merck, Darmstadt, Germany
Centrifuge	Micro Star 17 R	VWR International, Darmstadt, Germany
Centrifuge	Avanti J	Beckman Coulter, Brea, USA

Buffer preparation

SDS-running buffer (5X):	15 g tris<br>72 g glycine<br>5 g SDS<br>Fill to 1 L with dest. water<br>(Store at 4 °C)<br>Dilute with dest. water before usage
SDS-solution (0.1 g/mL):	1 g SDS (**toxic** ☠☠☠)<br>Fill to 10 mL with dest. water<br>(Store at RT)
1.5 M Tris-HCl, pH 8.8:	100 mL dest. water<br>90.75 g Tris<br>Adjust pH 8,8 with 4 M HCl (with 6M HCl about 41 ml for 500 mL buffer)<br>Fill to 500 mL with dest water Note final pH value on the bottle<br>(Store at 4 °C)
0.5 M Tris-HCl, pH 6.8:	100 mL dest. water<br>15 g Tris<br>Adjust pH 6.8 with 1 M HCl

	Fill to 250 mL with dest. water<br>Note final pH value on the bottle<br>(store at 4 °C)
Bromophenol blue-solution (5 mg/ml):	100 mL dest. water<br>0.5 g bromophenol blue<br>(Store at RT)
SDS-sample buffer:	50 mL dest. water<br>12.5 mL 0.5 M Tris-HCl, pH 6.8<br>10 mL glycerol<br>20 mL SDS-solution (0.1 g/mL)<br>5 mL mercaptoethanol (**toxic** ☠☠☠, fume hood)<br>1 mL bromophenol blue-solution (5 mg/ml)<br>(Store at 4 °C)
APS-solution (0.1 g/mL):	10 mL dest. water<br>1 g APS<br>Prepare aliquoits à 1 mL<br>(Store at -20 °C)
Polyacrylamide solution (30%):	Buy ready to use (**toxic** ☠☠☠, fume hood)<br>(Store at 4 °C)
Water-saturated butanol:	100 mL dest. water<br>100 mL butanol<br>(Top phase is butanol)
Fixation solution (1 L):	329 mL dest. water<br>571 mL ethanol (70%)<br>100 mL acetic acid (100%)
Coomassie Brilliant Blue Stock Solution:	5 g Coomassie Brilliant Blue G-250<br>100 mL dest. water<br>(store at RT)
Colloidale Coomassie Stock Solution:	50 g ammonium sulfate<br>484 mL dest. water<br>6 mL ortho-phosphoric acid (85%)<br>10 mL Coomassie Brilliant Blue Stock solution

(Store at RT, acid cupboard)

Colloidale Coomassie Dye Solution: 200 mL Colloidale Coomassie Stock Solution
50 mL methanol
(Store at RT, solvent cupboard)

Execution:

- Clean glass plates and spacer with ethanol and a fuzz free wipe
- Insert both into the clips. Proof whether the plates terminate flat on the ground
- Assemble gel casting device
- Prepare **12% separation gel**

1 mm Separation Gel [ml]	1 Gel	2 Gels	4 Gels	6 Gels	8 Gels
VE-Water	2.23	4.47	8.93	13.4	17.87
1.5 M Tris-HCl pH 8.8	1.67	3.33	6.67	10	13.33
SDS (10%)	0.07	0.13	0.27	0.4	0.53
Polyacrylamide	2.67	5.33	10.67	16	21.33
APS	0.05	0.10	0.20	0.3	0.40
TEMED	0.005	0.01	0.02	0.03	0.04
Total volume	**6.69**	**13.38**	**26.75**	**40.13**	**53.51**
**1.5 mm Separation Gel [ml]**	**1 Gel**	**2 Gels**	**4 Gels**	**6 Gels**	**8 Gels**
VE-Water	3.35	6.7	13.4	20.1	26.8
1.5 M Tris-HCl pH 8.8	2.5	5	10	15	20
SDS (10%)	0.1	0.2	0.4	0.6	0.8
Polyacrylamide	4	8	16	24	32
APS	0.075	0.15	0.3	0.45	0.6
TEMED	0.0075	0.015	0.03	0.045	0.06
Total volume	**10.04**	**20.07**	**40.13**	**60.20**	**80.26**

- Overlay liquid gel with water-saturated butanol and let it polymerize for 30 min
- Remove butanol and wash it with dest. water, remove remaining water with a fuzzy free wipe
- Prepare **4% stacking gel**

1 mm Stacking Gel [ml]	1 Gel	2 Gels	4 Gels	6 Gels	8 Gels
VE-Water	2.03	4.07	8.13	12.2	16.27
0.5 M Tris-HCl pH 6.8	0.83	1.67	3.33	5	6.67
SDS (10%)	0.03	0.07	0.13	0.2	0.27
Polyacrylamide	0.43	0.87	1.73	2.6	3.47
Bromophenol blue	0.013	0.03	0.05	0.08	0.11
APS	0.025	0.05	0.10	0.15	0.20
TEMED	0.005	0.01	0.02	0.03	0.04

**Total volume**	**3.38**	**6.75**	**13.51**	**20.26**	**27.01**
**1.5 mm Stacking Gel [ml]**	**1 Gel**	**2 Gels**	**4 Gels**	**6 Gels**	**8 Gels**
**VE-Water**	3.05	6.1	12.2	18.3	24.4
**0.5 M Tris-HCl pH 6.8**	1.25	2.5	5	7.5	10
**SDS (10%)**	0.05	0.1	0.2	0.3	0.4
**Polyacrylamide**	0.65	1.3	2.6	3.9	5.2
**Bromophenol blue**	0.02	0.04	0.08	0.12	0.16
**APS**	0.0375	0.75	0.15	0.025	0.3
**TEMED**	0.0075	0.15	0.03	0.045	0.06
**Total volume**	**50.58**	**10.13**	**20.23**	**30.345**	**40.46**

- Add comb airbubble free on the stacking gel **(Pay attention to splashing solution, wear glasses)**
- Remove comb after 30 min polymerization time and wash pockets with dest. water
- Assemble each 2 gels with the spacer outside into the electrophoresis chamber.
- Fill chamber with SDS-running buffer (1x). Remove airbubbles below the gel by pipetting
- Fill space into the chambers also with SDS-running buffer **(1x)**
- Fill 10-20 ml sample into each pocket (30 µl into 1.5 mm gels) and add 2 µL of the protein standard to one pocket (note name of the standard)
- Close the chamber and connect to power supply
- Adjust for each gel 10 mA current until the migration front entered the separation gel and then 20 mA current. Stop short before the migration front has left the gel.
- After disconnecting the power supply remove the gels, as well as fixate and dye them (see also SOP 5.1 Coomassie staining)

Sample preparation:

- Dilute your sample in at least the same amount of SDS-sample buffer
- Shake 5 min at 60 °C and 1.400 rpm on the
- Centrifuge at 16,400 xg and RT for 10 min
- Load 20 µL sample into each pocket

or

- Precipitate 110% of required sample amount
- Add same volume of dest. water to your sample
- Precipitate with fivefold of icecold acetone (100%)
- Incubate at least 1 h or overnight
- Centrifuge at 16,400 xg and RT for 10 min
- Discard supernatant and dry pellet

- Add 22 µL SDS-sample buffer to the dried protein pellet and shake 5 min at 60 °C and 1,400 rpm on the thermomixer → adapt buffer volume and amount of precipitated protein to get 100%
- Centrifuge at 16,400 xg and RT for 10 min
- Load 20 µL sample into each pocket

Coomassie Staining

- Place gels after electrophoresis into marked bowls
- Add fixation solution for at least 1 h on a shaker with 20 rpm.
- Wash twice with dest. Water
- Add Colloidale Coomassie Dye Solution overnight at RT on a shaker with 20 rpm.
- Wash with dest. Water

Encloser:

- not applicable

Abbreviations:

APS	ammonium persulfate
dest. water	deionized water
DTT	dithiothreitol
M	molare
RT	room temperature
TEMED	tetramethylethyldiamin
SDS	sodium dodecyl sulfate

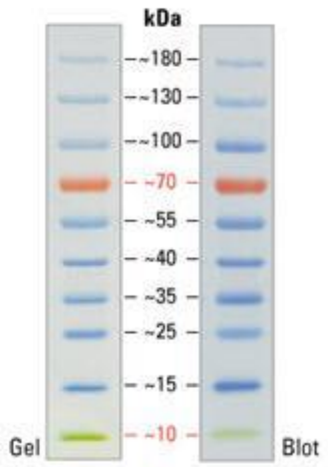

Figure 1: PageRuler Prestained Protein Ladder #26616, LOT independent molecular weights.

SOP: Peptide Electrophoresis

Application:

The peptide electrophoresis enables the quality control for the tryptic digestion. See also the publication of *Shägger et al. (2006)* [1].

1. Schägger H,. 2006. Tricine-SDS-PAGE. Nat Protoc,1(1):16-22.

Remarks:

Acrylamide, mercaptoethanol and methanol are toxic! It is recommended to execute all steps under a fume hood. It is also advised to wear a pair of safety glasses.

For pH adjustement of the Tris/HCl-solutions different concentrations of HCl can be used. However, it has to be taken into account that a bigger volume is needed, when acids with smaller concentration are used. Do not exceed the maximum volume stated.

Before assembling the plates check the size of the gels plates (1 mm or 1.5 mm), combs and gel volumes.

The voltage of the electrophoresis can be set to values lower than the ones described. Keep in mind that this will lead to an increase in running time.

Important for the success of the peptide gel electrophoresis is a low concentration of SDS in the sample buffer. Therefore, do not forget to dilute the buffer 1:4 before use. Also do not exceed the recommended sample volume of 10 µl. To avoid diffusion of peptides after the run, use a fixation-solution containing methanol.

Gel preparation takes approx. 2h. The electrophoresis runs for about 3h!

Chemicals

Table 1: Used chemicals. Named manufacturer as well as order numbers are not mandatory and can be replaced by others with equal quality.

Chemicals	Manufacturer	Order Numbers	Storage place
Acetic Acid ≥ 96%	Carl Roth	T179.2	
Acetone, ≥ 99%.,	VWR International	20063.296	
Acrylamide solution 30% (**toxic** ☠☠☠)	Serva	10688.01	
Ammonium acetate	Carl Roth	7869.1	
Ammonium sulfate	Carl Roth	3746.3	
Ammonium persulfate (APS)	Merck	1.01201.0500	
Bromophenol blue	GE Healthcare	17-1329-01	
Coomassie BB G 250	AppliChem	A3484.0100	
Ethanol (70%)	Carl Roth	T913.3	
Glycerol	Carl Roth	3783.1	
Glycine	VWR International	444495D	

Hydrochloric acid (37%)	VWR International	20252.420	
Isobutanol	AppliChem	A1150.1000	
Mercaptoethanol (**toxic** ☠☠☠)	Sigma	M3148	
Methanol ≥ 99.9% (**toxic** ☠☠☠)	Carl Roth	T909.1	
Protein standard	Thermo Scientific PageRuler Prestained	Protein Ladder #26616	
Phosphoric acid ≥ 85%	Carl Roth	6366.2	
SDS (sodium dodecyl sulfate,(**toxic** ☠☠☠)	AppliChem	A2572.0250	
TEMED	Carl Roth	2367.1	
Tris	AppliChem	A2264.1000	
Tricine	Sigma	T0377	

Devices

Table 2: Used devices. Devices and manufacturers are not mandatory and can be replaced by others with equal functionality.

Device	Name	Manufacturer
Electrophoresis chamber	Mini-Protean Tetra System	BioRad (Hercules, USA)
Power supply	PowerPac Basic	BioRad (Hercules, USA)
Destilled water	Millipore Q-POD	Merck, Darmstadt, Germany
Centrifuge	Micro Star 17 R	VWR International, Darmstadt, Germany
Centrifuge	Avanti J	Beckman Coulter, Brea, USA

Buffer preparation

Anode Buffer (pH 8.9)
dissolve 12.11 g Tris in 500 mL MilliQ-Water
adjust pH value with 6M HCl (approx. 3 mL)
fill up to 1 L with MilliQ-Water
(store at 4 °C)

APS-Solution (10%)
10 mL MilliQ-Water
1 g APS
prepare aliquots of 1 mL
(store at -20 °C)

Cathode Buffer
12.11 g Tris
17.92 g Tricine
1 g SDS
fill up to 1 L with MilliQ-Water
(store at 4 °C)

Polyacrylamide-Solution (30%) buy ready to use (fume hood, toxic ☠☠☠)

	(store at 4 °C)
Sample Buffer (4x)	6 g SDS (fume hood, toxic ☠☠☠)<br>3 mL Mercaptoethanol (fume hood, toxic ☠☠☠)<br>11.90 mL Glycerol<br>25 mg Coomassie BB G-250<br>fill up to 50 mL with 150 mM Tris/HCl<br>(Store at 4 °C)
150 mM Tris/HCl (pH 7.0)	dissolve 1.28 g Tris in 50 mL MilliQ-Water<br>adjust pH value with 6 M HCl<br>fill up to 100 mL with MilliQ-Water
3 M Tris-Gel Buffer (pH 8.45)	90.86 g Tris<br>0.75 g SDS<br>dissolve in 200 mL MilliQ-Water<br>adjust pH value with 6 M HCl (> 40 mL)<br>fill up to 250 mL with MilliQ-Water<br>(Store at 4 °C)
Water-saturated Isobutanol:	100 mL MilliQ-Water<br>100 mL Isobutanol

Solution Preparation for Coomassie-staining:

Coomassie-Dye-Solution	270 mL MilliQ-Water<br>30 mL Acetic Acid (100%)<br>75 mg Coomassie BB G250
Destaining-Solution	450 mL MilliQ-Water<br>50 mL Acetic Acid (100%)
Fixation-Solution	200 mL MilliQ-Water<br>250 mL Methanol (100%) (fume hood, toxic ☠☠☠)<br>50 mL Acetc Acid (100%)<br>3.854 g Ammonium Acetate<br>(prepare fresh!, store at RT)<br>(Methanol **cannot** be replaced by Ethanol, because Ethanol do not precipitate the peptides)

Execution:

- Clean glass plates and spacer with ethanol and fuzz-free wipes and clamp them into the casting frames. Make sure the bottom of both plates lines up evenly.
- Assemble gel casting device.
- Prepare gels using the following table (sufficient for 4 gels):

1 mm Separation Gel [ml]	Separating gel (16%)	Spacer gel (10%)	Stacking gel (4%)
MilliQ-Water [mL]	1.51	9.84	7.30
3 M Tris-Gel Buffer [mL]	10	10	3
Glycerol [mL]	2.38	–	–
Acrylamide-Solution [mL]	16	10	1.6
APS-Solution [mL]	0.1	0.15	0.09
TEMED [mL]	0.01	0.015	0.009
Total volume [mL]	30	30	12

- Prepare 16% separating gel. Leave room (1-2 cm) for 10% gel and stacking gel!
- Cast 10% spacer gel on top of the still liquid 16% gel and overlay with water-saturated butanol.
- After polymerization (approx. 30 min) discard butanol and rinse gel surface with MilliQ-Water.
- Remove water with fuzz-free wipe.
- Pour 4% stacking gel on top of the 10% gel.
- Insert comb airbubble-free into the liquid stacking gel **(Beware of splashs: wear safety goggles!)**
- After polymerization (approx. 30 min) remove comb carefully and rinse pockets with MilliQ-Water.
- Assemble each two gels with the bigger plate to the outer side and place everything into the electrophoresis chamber. Fill inner chamber with Cathode Buffer and outer chamber up to the mark with Anode Buffer. Remove airbubbles under the gel with a 10 mL pipette.
- Load 10 µl of prepared sample per pocket and add 2 µl of a prestained protein or peptide standard to an empty pocket (note the name of the standard).
- Close the chamber and connect to power supply.
- Choose a constant voltage of 30 V until the migration front reached the 10% spacer gel. Then switch to 90 V (note the current [mA] at the beginning and at the end). Stop power, when the migration front reached the end of the gel. Avoid leakage.
- After disconnecting the power supply remove the gels. They can now be fixed and dyed (see below) or blotted (see SOP 7.1 for Western Blot).

<u>**Sample preparation:**</u>

- Use digested samples (see SOP 6.3 for in-gel digest or SOP 6.4 for FASP digest)
- Add 10 µL of the 1:4-diluted sample buffer to the protein pellet and incubate for 60 min at 37°C and 1,400 rpm on a thermomixer.

- Then, centrifuge at 16,400 xg and RT for 10 min
- Load 10 µl of sample per gel pocket.

Coomassie-staining for peptide gels:

- **Exclusively use the fixation-, staining-, and destaining-solutions described in this protocol!**
- Place gels after electrophoresis into marked bowls
- Add fixation solution for at least 1 h on a shaker with 20 rpm.
- Wash twice with dest. Water
- Add Colloidale Coomassie Dye Solution overnight at RT on a shaker with 20 rpm.
- For the destaining process incubate gels for 2x 15-60 min in destaining solution whilst shaking (30 rpm). Wash further in MilliQ-Water until desired contrast is attained.

Encloser:

- not applicable

Abbreviations:

APS	ammonium persulfate
BSA	bovine serum albumin
dest. water	deionized water
DTT	dithiothreitol
M	molare
RT	room temperature
SDS	sodium dodecyl sulfate
TEMED	tetramethylethyldiamine
