# Supplementary figures and images for: A Robust and Universal Metaproteomics Workflow for Research Studies and Routine Diagnostics Within 24 h Using Phenol Extraction, FASP Digest, and the MetaProteomeAnalyzer
Source: Front Microbiol. 2019 Aug 16;10:1883. doi: 10.3389/fmicb.2019.01883 (PMC6707425; doi:10.3389/fmicb.2019.01883)

## Hgut 1A: In-gel digestion

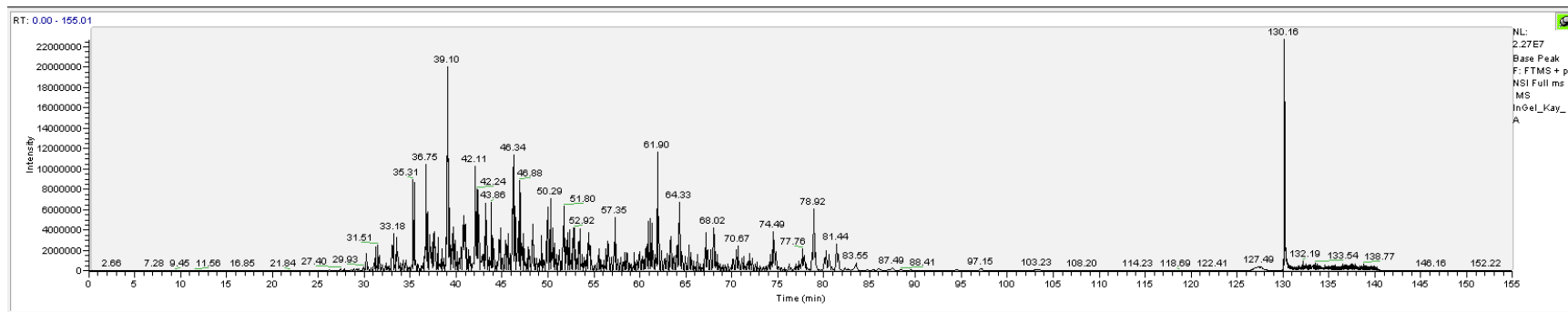

## Hgut 1A: FASP digestion, acidified and directly injected

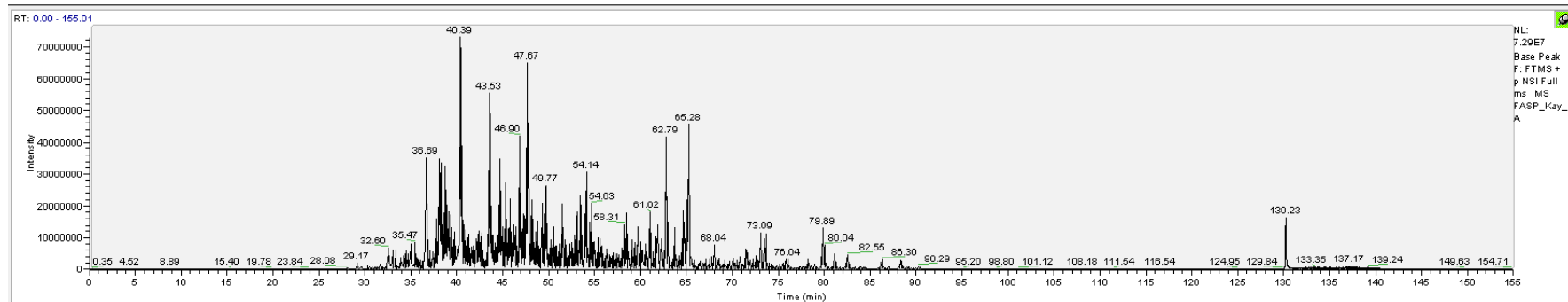

Supplement: DATA SHEET S2 — Chromatograms. [file Data_Sheet_2.PDF]
