# Supplementary material for: A Robust and Universal Metaproteomics Workflow for Research Studies and Routine Diagnostics Within 24 h Using Phenol Extraction, FASP Digest, and the MetaProteomeAnalyzer
Source: Front Microbiol. 2019 Aug 16;10:1883. doi: 10.3389/fmicb.2019.01883 (PMC6707425; doi:10.3389/fmicb.2019.01883)
Supplement: PRESENTATION S1 — Quality control gels. [file Presentation_1.PPTX]

## Slide 1
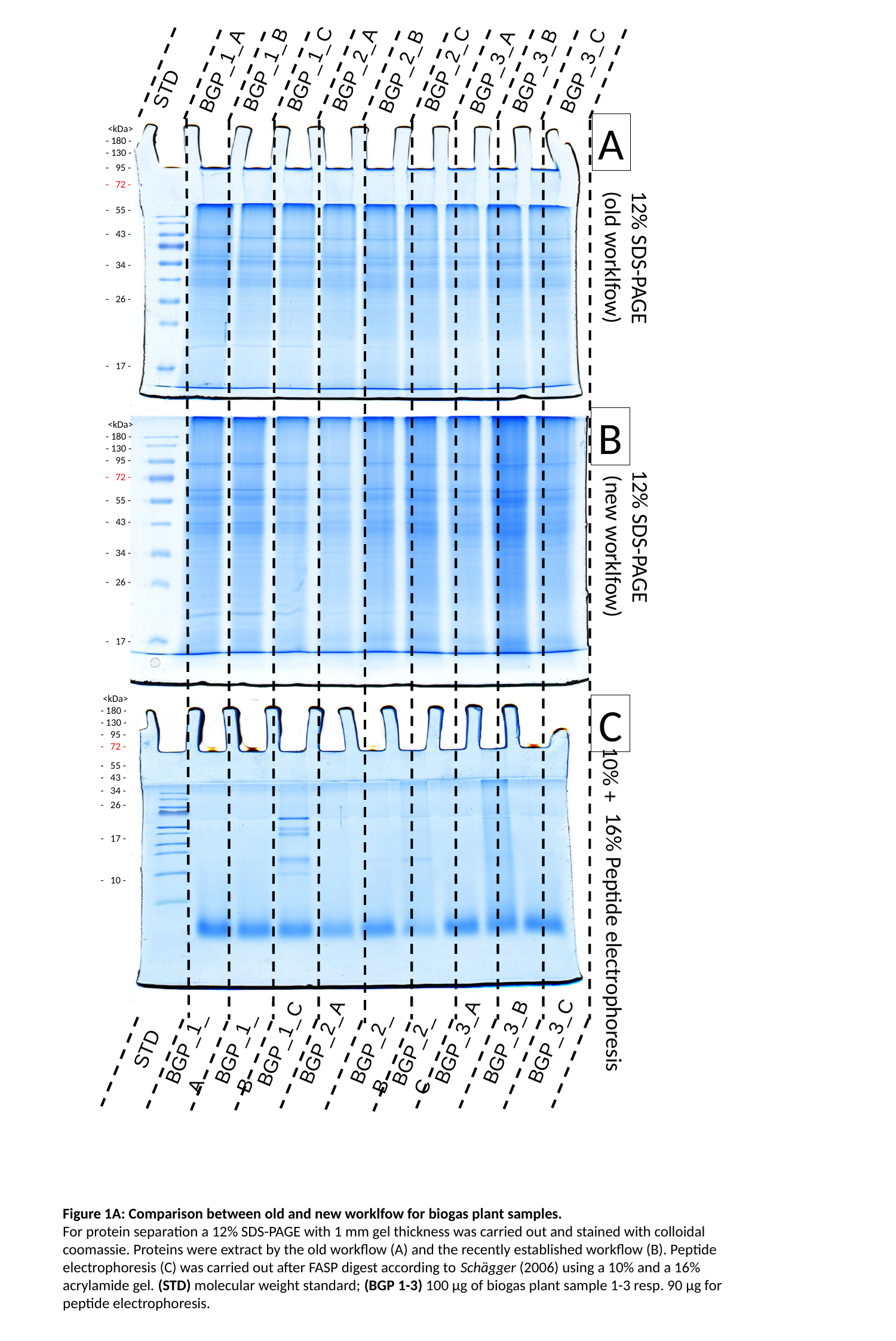

BGP_2_B
BGP_3_B
BGP_2_C
BGP_3_A
BGP_1_C
BGP_1_A
BGP_2_A
BGP_1_B
BGP_3_C
STD
 <kDa>
- 180 -
- 130 -
- 95 -
- 72 -
- 55 -
- 43 -
- 34 -
- 26 -
- 17 -
A
12% SDS-PAGE
(old worklfow)
B
 <kDa>
- 180 -
- 130 -
- 95 -
- 72 -
- 55 -
- 43 -
- 34 -
- 26 -
- 17 -
12% SDS-PAGE
 (new worklfow)
 <kDa>
- 180 -
- 130 -
- 95 -
- 72 -
- 55 -
- 43 -
- 34 -
- 26 -
- 17 -
- 10 -
C
10% +
16% Peptide electrophoresis
BGP_1_C
BGP_3_A
BGP_2_A
BGP_3_B
BGP_3_C
BGP_1_A
BGP_1_B
BGP_2_C
BGP_2_B
STD
Figure 1A: Comparison between old and new worklfow for biogas plant samples.
For protein separation a 12% SDS-PAGE with 1 mm gel thickness was carried out and stained with colloidal coomassie. Proteins were extract by the old workflow (A) and the recently established workflow (B). Peptide electrophoresis (C) was carried out after FASP digest according to Schägger (2006) using a 10% and a 16% acrylamide gel. (STD) molecular weight standard; (BGP 1-3) 100 µg of biogas plant sample 1-3 resp. 90 µg for peptide electrophoresis.

## Slide 2
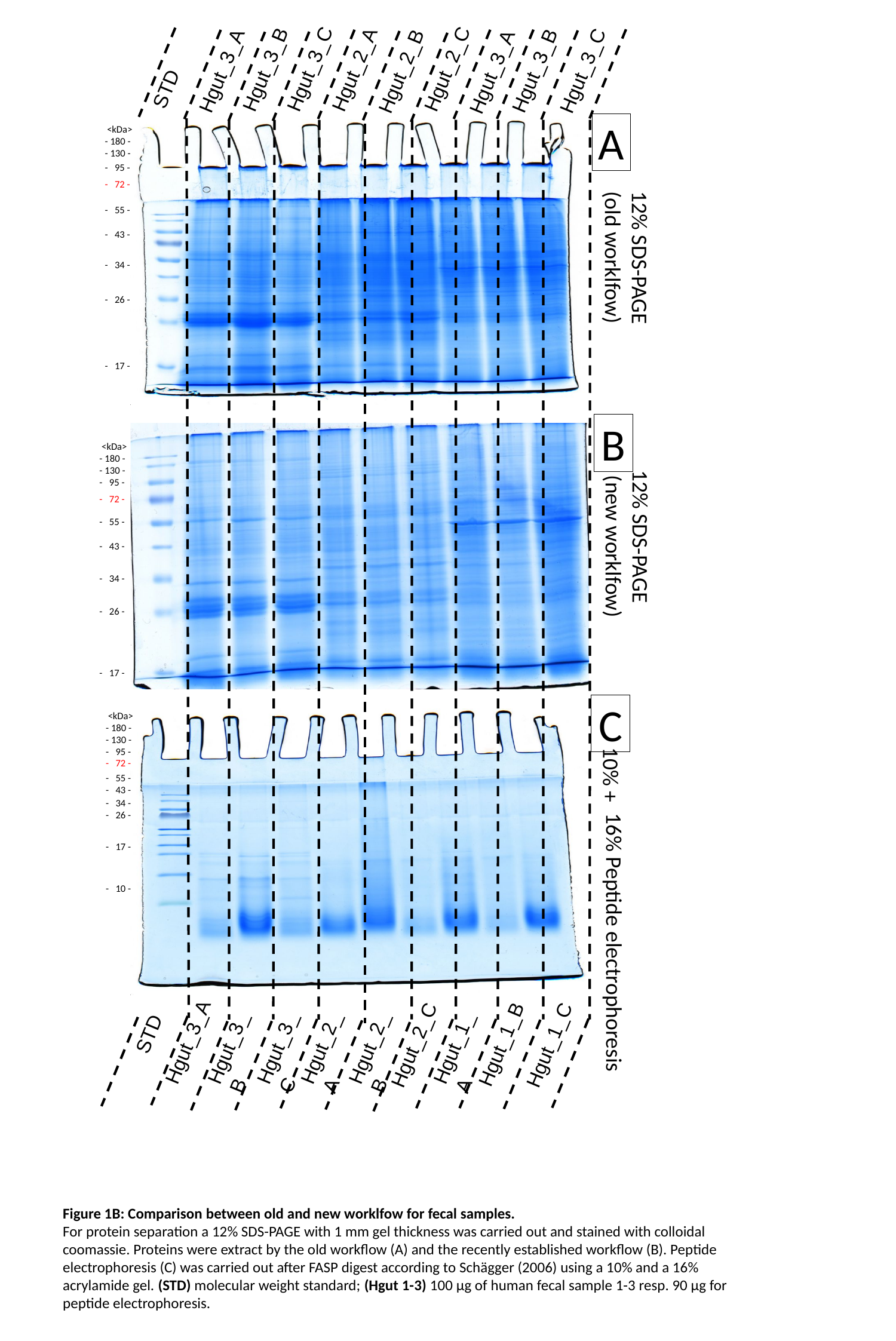

Hgut_3_B
Hgut_2_C
Hgut_2_B
Hgut_3_A
Hgut_3_C
Hgut_3_B
Hgut_3_A
Hgut_2_A
Hgut_3_C
STD
A
 <kDa>
- 180 -
- 130 -
- 95 -
- 72 -
- 55 -
- 43 -
- 34 -
- 26 -
- 17 -
12% SDS-PAGE
(old worklfow)
B
 <kDa>
- 180 -
- 130 -
- 95 -
- 72 -
- 55 -
- 43 -
- 34 -
- 26 -
- 17 -
12% SDS-PAGE
 (new worklfow)
C
 <kDa>
- 180 -
- 130 -
- 95 -
- 72 -
- 55 -
- 43 -
- 34 -
- 26 -
- 17 -
- 10 -
10% +
16% Peptide electrophoresis
Hgut_1_B
Hgut_3_A
Hgut_1_C
Hgut_2_C
Hgut_3_B
Hgut_3_C
Hgut_2_B
Hgut_1_A
Hgut_2_A
STD
Figure 1B: Comparison between old and new worklfow for fecal samples.
For protein separation a 12% SDS-PAGE with 1 mm gel thickness was carried out and stained with colloidal coomassie. Proteins were extract by the old workflow (A) and the recently established workflow (B). Peptide electrophoresis (C) was carried out after FASP digest according to Schägger (2006) using a 10% and a 16% acrylamide gel. (STD) molecular weight standard; (Hgut 1-3) 100 µg of human fecal sample 1-3 resp. 90 µg for peptide electrophoresis.

## Slide 3
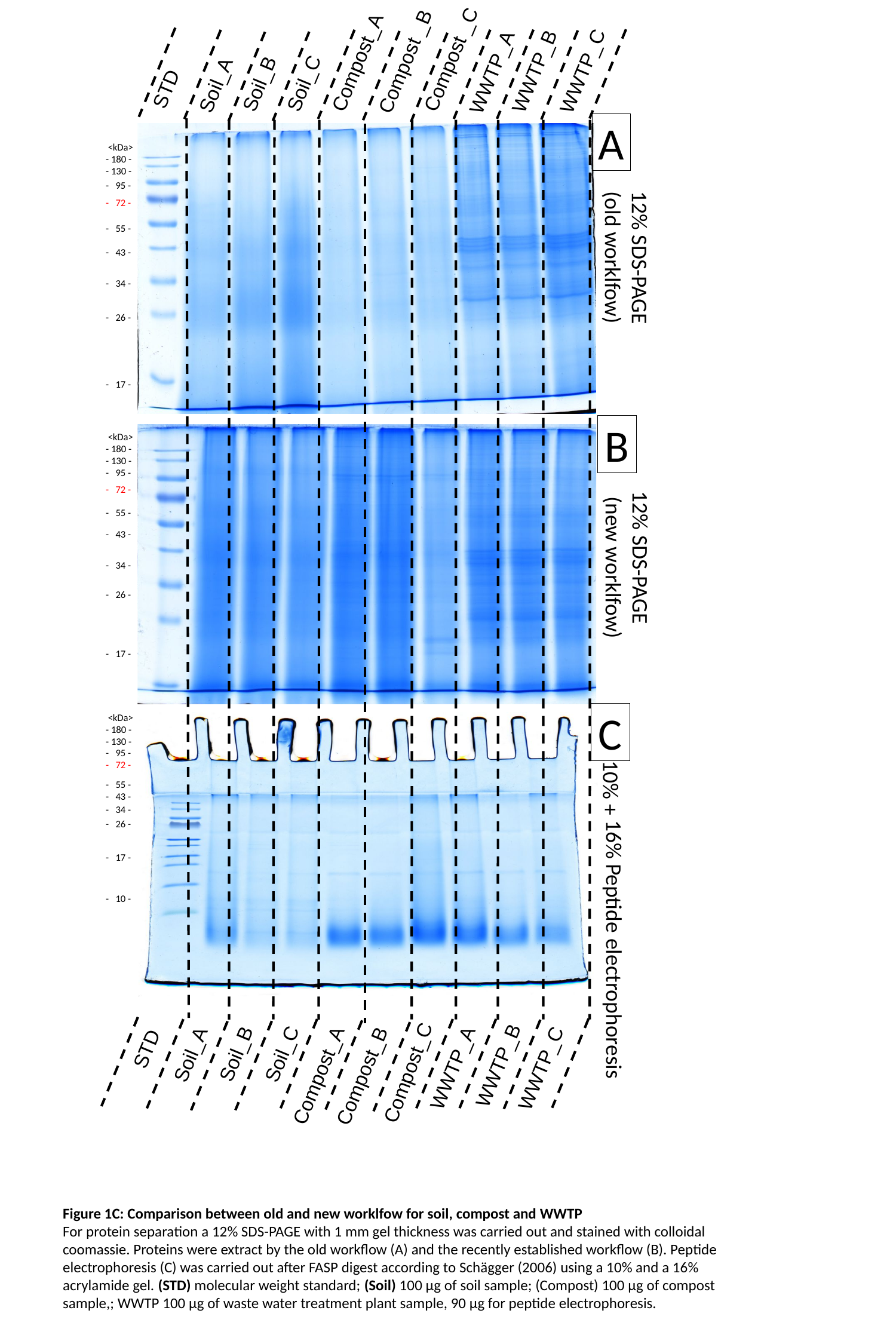

WWTP_A
WWTP_B
STD
Soil_B
Soil_C
Compost_A
Compost _C
Soil_A
Compost _B
WWTP_C
A
 <kDa>
- 180 -
- 130 -
- 95 -
- 72 -
- 55 -
- 43 -
- 34 -
- 26 -
- 17 -
12% SDS-PAGE
(old worklfow)
B
 <kDa>
- 180 -
- 130 -
- 95 -
- 72 -
- 55 -
- 43 -
- 34 -
- 26 -
- 17 -
12% SDS-PAGE
 (new worklfow)
 <kDa>
- 180 -
- 130 -
- 95 -
- 72 -
- 55 -
- 43 -
- 34 -
- 26 -
- 17 -
- 10 -
C
10% +
16% Peptide electrophoresis
Compost_C
WWTP_B
Soil_C
WWTP_C
Soil_B
Compost_A
WWTP_A
Compost_B
Soil_A
STD
Figure 1C: Comparison between old and new worklfow for soil, compost and WWTP
For protein separation a 12% SDS-PAGE with 1 mm gel thickness was carried out and stained with colloidal coomassie. Proteins were extract by the old workflow (A) and the recently established workflow (B). Peptide electrophoresis (C) was carried out after FASP digest according to Schägger (2006) using a 10% and a 16% acrylamide gel. (STD) molecular weight standard; (Soil) 100 µg of soil sample; (Compost) 100 µg of compost sample,; WWTP 100 µg of waste water treatment plant sample, 90 µg for peptide electrophoresis.
